# Global, regional, and national burden of type 2 diabetes-related diabetic kidney disease attributable to low physical activity from 1990 to 2021: a systematic analysis of the Global Burden of Disease Study 2021 with predictions to 2050

**DOI:** 10.3389/fendo.2025.1625973

**Published:** 2025-09-17

**Authors:** Mei Wang, Ruihua Yan, Wenbo Xia, Yongcai Gao, Yonghua Liu, Li Bao, Hongyan Luo, Jing E, Hui Wang, Bo Li, Yali Zheng

**Affiliations:** ^1^ Department of Nephrology, People’s Hospital of Ningxia Hui Autonomous Region, Yinchuan, Ningxia, China; ^2^ Department of Obstetrics and Gynecology, People’s Hospital of Ningxia Hui Autonomous Region, Yinchuan, Ningxia, China; ^3^ The Third Clinical Medical College, Ningxia Medical University, Yinchuan, Ningxia, China

**Keywords:** low physical activity, diabetic kidney disease, Global Burden of Disease, deaths, DALYs

## Abstract

**Background:**

Low physical activity (LPA) significantly heightens the susceptibility of both type 2 diabetes mellitus (T2DM) and chronic renal disease. Nearly half of population diagnosed with T2DM globally worsen into diabetic kidney disease (DKD). Focusing on physically inactive populations, we aimed to comprehensively evaluate the trends over time and regional changes in T2DM-associated DKD attributable to LPA burden.

**Methods:**

We utilized data of the 2021 Global Burden of Disease (GBD) Study to initially assess the worldwide effects of T2DM-associated DKD attributable to LPA by computing the numbers and age-standardized rates (ASRs) of death, disability-adjusted life years (DALYs), years of life lost (YLLs), and years lived with disability (YLDs), categorized by subtypes in 2021. Linear regression model was applied to analyze the illness burden from 1990 to 2021. Furthermore, cluster analysis was performed to assess the regional differences in disease burden across GBD regions. Lastly, to forecast the illness burden for the next 25 years, we utilized the autoregressive Integrated Moving Average (ARIMA) and Excess Risk (ER) models.

**Results:**

In 2021, the fatalities attributed to T2DM-related DKD attributable to LPA amounted to 30835 (95%UI: 12346-51646) cases, with 698484 (95%UI: 275039-1158032) DALYs. The ASRs of death and DALYs were 0.38 (95%UI: 0.15-0.63) and 8.19 (95%UI: 3.21-13.6) per 100000 individuals, respectively. Between 1990 and 2021, there was a notable escalation in deaths, DALYs, YLDs, and YLLs, as well as their ASRs. The highest burden was observed among males, older adults (aged 70 years and above), and middle Socio-demographic Index (SDI). Significant differences were noted in the disease burden among various regions and countries as defined by the GBD study. Predictive analyses indicate a continued escalation of this burden by the year 2050.

**Conclusions:**

The global impact of DKD attributable to LPA remains considerable, with significant disparities noted across different genders, ages, and regions. To mitigate this burden, it is crucial to implement effective interventions aimed at addressing physical inactivity, specifically designed for targeted demographic groups.

## 1 Introduction

Type 2 diabetes mellitus (T2DM) persists as the principal contributor to chronic kidney disease (CKD) development worldwide, with roughly 50% of those affected progressing to diabetic kidney disease (DKD) (1, 2). DKD now represents a key element in fastest-growing chronic non-communicable disorders. The latest study on Global Burden of Disease (GBD) (3) reveals that between 1990 and 2021, the global prevalence of DKD linked to T2DM increased by 85.5%. In 2019, DKD mediated 0.5 million deaths and resulted in 13 million disability-adjusted life years (DALYs) worldwide. All age-standardized rates (ASRs) for incidence and death from T2DM-related CKD increased over 30 years (4). The clinical presentation of DKD includes albuminuria and progressive renal function loss, leading to end-stage renal disease, which is tied to a markedly elevated risk of cardiovascular complications and mortality (5). The considerable health burdens and economic impacts of DKD present a serious threat to global public health (6). Therefore, enhancing the prevention and treatment of DKD globally is essential to alleviate its detrimental impact on human health.

Multiple studies have demonstrated that physical activity is conducive to health (7). The association between low physical activity (LPA) and DKD has been widely established. LPA lifestyle is intimately linked to the development of DKD (8, 9). Prolonged sedentary behavior and a lack of exercise can contribute to obesity and bone and muscle wasting of kidney disease (10). Recent data indicate that patients at risk of DKD or with existing DKD who engage in regular moderate-to-vigorous physical activity experience a decreased incidence and progression of DKD, along with a diminished risk of mortality (11). These beneficial effects are achieved through improving endothelial function, insulin sensitivity, inflammatory response, and other aspects (12–15). A population-based cohort study included 475,376 adults with normal kidneys at baseline from China and suggested that PA was inversely correlated with the incidence of DKD during a median follow-up of 12.1 years, with PA measured in metabolic equivalent of task hours per day (MET-h/day) (9). One study reported that PA could prevent the development of sarcopenia and maintain muscle mass in CKD patients after 12 months of exercise training (16). Higher PA is associated with better renal outcomes in advanced DKD in a 5-year prospective cohort study in Japan (17).

Despite these compelling findings, extensive worldwide epidemiological research investigating the influence of LPA on the burden of DKD continues to be limited. In 2019, worldwide fatalities attributed to LPA reached 0.83 million, alongside 15.75 million DALYs. That year, T2DM was identified as the second prominent cause of LPA-related DALYs on a worldwide scale (18). The established causal link between T2DM and DKD indicates that the growing burden of LPA-related T2DM correlates with a heightened impact of LPA-related DKD (19). Therefore, it is essential to comprehensively grasp the burden of DKD owing to LPA within this context.

This study aimed to conduct a systematic assessment of the disease burden for LPA-related DKD in T2DM from 1990 to 2021, utilizing data from GBD 2021. Furthermore, we will forecast these trends to 2050, offering important insights for future public health planning and the allocation of resources pertaining to DKD due to LPA.

## 2 Methods

### 2.1 Data source

The data in this investigation was extracted from GBD 2021 study database. This study conducted a thorough and systematic assessment of 371 diseases and injuries, 81 risk factors across 204 nations and territories, spanning the years 1990 to 2021 (20). Based on the geographical distribution, 204 nations and territories were grouped into 21 super regions and 54 regions following GBD taxonomy, which also incorporates World Bank classifications (21). Furthermore, these nations and territories were classified into five socio-demographic index (SDI) levels (low, low-middle, middle, high-middle, and high SDI scores) according to SDI index that ranges of 0 to 1. This index indicates a nation’s or region’s progress in relation to birth rates, educational achievement, and per capita income.

### 2.2 Study data

In this study, we gathered data on the numbers of cases and the corresponding ASRs of T2DM-related DKD attributable to LPA collected from 1990 through 2021, including death, DALYs, years of life lost (YLLs), and years lived with disability (YLDs). The calculation of DALYs involves summing YLDs and YLLs. We utilized these metrics to characterize the disease burden. Each rate is reported per 100000 individuals, with 95% uncertainty interval (UI) (22) at gender, age, SDI, GBD regions, and countries. The dynamics of DKD due to LPA were analyzed by calculating Estimated Annual Percentage Change (EAPC) to identify temporal changes in illness burden with 95% Confidence Intervals (CIs) of EAPCs by linear modeling from 1990 to 2021. In the GBD study, the assessment of disease burden attributable to LPA was aimed at adults aged 25 years and older; the population was further segmented into 15 age groups, each spanning 5 years: 25-29, 30-34, 35-39, 40-44, 45-49, 50-54, 55-59, 60-64, 65-69, 70-74, 75-79, 80-84, 85-89, 90-94, and 95+ years. The ethical review by the Ethical Board of People’s Hospital of Ningxia Hui Autonomous Region was not required for this study protocol, since GBD data were anonymized and presented in an aggregated format.

### 2.3 Definition of T2DM-related DKD and low physical activity

In the GBD 2021 study, DKD due to T2DM was defined as ICD-10 code E11.2-E11.29 (23). Specifically, the definition of T2DM indicates a fasting plasma glucose value of ≥7 mmol/L (≥126 mg/dL) or administration of diabetes medications or insulin (24).DKD is characterized by persistent kidney dysfunction lasting over three months, identified through either an increased urinary albumin-to-creatinine ratio (UACR ≥30 mg/g) and/or lower estimated glomerular filtration rate (eGFR <60 mL/min/1.73 m²) (25).

Data on physical activity (PA) were collected using standardized surveys: the International Physical Activity Questionnaire (IPAQ) and the Global Physical Activity Questionnaire (GPAQ). More specifically, PA was quantified in total metabolic equivalent (MET) minutes weekly, integrating the frequency and duration of various activities coupled with associated MET values based on each activity’s intensity (26). PA levels were classified into four categories according to the quartiles of total global METs: inactive (<600 MET-minutes/week), low-active (600–3999 MET-minutes/week), moderately active (4000–7999 MET-minutes/week), and very active (>=8000 MET-minutes/week) (27). According to GBD 2021 study, the theoretical minimum risk exposure level of physical inactivity ranged from 3600 to 4400 MET-minutes/week, therefore, LPA was defined as <3600 MET-minutes/week (28).

### 2.4 Statistical analysis

Initially, the cases of deaths, DALYs, YLLs, and YLDs of DKD due to LPA and their corresponding ASRs were documented for the year 2021 at the global level and across various subcategories, including age, sex, SDI, GBD regions, and nations. Second, we assessed the worldwide evolution of disease burden across distinct areas and subtypes between 1990 and 2021. The EAPC was derived from ASRs calculations to assess the change over time with linear regression model. A hierarchical cluster analysis was conducted utilizing the EAPC data to elucidate the evolving patterns in the DKD attributable to LPA pressure across each GBD region. This analysis aimed to pinpoint regions exhibiting analogous trends in disease burden over time. All 54 GBD regions were grouped into four distinct categories on associated EAPC values: (1) significant increase, (2) minor increase, (3) remained stable or minor decrease, and (4) significant decrease. We further used the Slope Index of Inequality and Concentration Index—standard metrics for absolute/relative gradient inequality—to measure the distribution inequality across the globe and five SDI regions. Health inequality monitoring underpins evidence-based health planning and refines initiatives to narrow health outcome disparities.

At last, the future burden of DKD attributable to LPA from 2021 to 2050 was projected using two advanced modeling approaches: AutoRegressive Integrated Moving Average (ARIMA) and Exponential Smoothing (ES) models. The ARIMA excels at capturing complex patterns, including trends and seasonality, while the ES model prioritizes recent observations, providing a robust and comprehensive framework for forecasting future disease burden across multiple dimensions. Statistical analyses were performed using R software (v4.4.2) for database construction, management, and analytical procedures.

## 3 Results

### 3.1 Global Burden of DKD attributable to LPA

The world burden of DKD associated with LPA in 1990 and 2021 is shown in **Tables 1**, **2** and **Supplementary Table S1**, **Supplementary Table S2**. In 1990, the DKD cases linked to LPA resulted in 11253 (95%UI: 4651-18635) deaths globally, with an age-standardized deaths rate of 0.33 (95%UI: 0.14-0.54) per 100000 people. Overall, there were 297698 (95%UI: 114900-493524) DALYs, consisting of 66127 (95%UI: 24806-116887) YLDs and 231571 (95%UI: 91970-388024) YLLs. By 2021, DKD due to LPA accounted for 30835 (95%UI: 12346-51646) deaths and 698484 (95%UI: 275039-1158032) DALYs, with 128134 (95%UI: 49280-226865) YLDs and 570350 (95%UI: 226001-965286) YLLs. The corresponding ASRs of death and DALYs were 0.38 (95%UI: 0.15-0.63) and 8.19 (95%UI: 3.21-13.6) per 100000 individuals, respectively.

**Table 1:** The number of deaths and age-standardized death rate of DKD attributable to low physical activity in 1990 and 2021 with EAPC from 1990 to 2021 globally.

Items	1990		2021		1990-2021
	Number of deaths (95% UI)	The age-standardized deaths rate/100000(95% UI)	Number of deaths(95% UI)	The age-standardized deaths rate/100000(95% UI)	EAPC(95%CI)
Global	11253 (4651-18635)	0.33 (0.14-0.54)	30835 (12346-51646)	0.38 (0.15-0.63)	1.22(0.83-1.61)
Sex					
Female	6261 (2611-10468)	0.32 (0.13-0.53)	17053 (6929-28269)	0.36 (0.15-0.6)	0.4(0.32-0.48)
Male	4992 (1968-8448)	0.36 (0.14-0.59)	13783 (5524-23345)	0.39 (0.16-0.66)	0.37(0.33-0.42)
Age groups					
25-29	7 (2-20)	0 (0-0)	8 (2-21)	0 (0-0)	-0.60(-0.73–0.48)
30-34	25 (6-59)	0.01 (0-0.02)	37 (10-87)	0.01 (0-0.01)	-0.21(-0.33–0.09)
35-39	70 (17-164)	0.02 (0-0.05)	107 (30-243)	0.02 (0.01-0.04)	-0.07(-0.21-0.07)
40-44	126 (37-278)	0.04 (0.01-0.1)	243 (76-538)	0.05 (0.02-0.11)	0.30(0.20-0.41)
45-49	219 (64-456)	0.09 (0.03-0.2)	494 (157-997)	0.1 (0.03-0.21)	0.40(0.33-0.47)
50-54	422 (139-836)	0.2 (0.07-0.39)	983 (348-1901)	0.22 (0.08-0.43)	0.36(0.32-0.40)
55-59	738 (260-1423)	0.4 (0.14-0.77)	1718 (638-3283)	0.43 (0.16-0.83)	0.27(0.18-0.35)
60-64	1130 (412-2087)	0.7 (0.26-1.3)	2350 (952-4445)	0.73 (0.3-1.39)	0.04(-0.07-0.16)
65-69	1441 (541-2650)	1.17 (0.44-2.14)	3269 (1237-5860)	1.19 (0.45-2.12)	0.02(-0.12-0.16)
70-74	1712 (631-3075)	2.02 (0.75-3.63)	4128 (1527-7190)	2.01 (0.74-3.49)	-0.01(-0.09-0.07)
75-79	1880 (773-3242)	3.05 (1.26-5.27)	4313 (1744-7624)	3.27 (1.32-5.78)	0.05(-0.02-0.11)
80-84	1632 (632-2876)	4.61 (1.79-8.13)	4508 (1824-7544)	5.15 (2.08-8.61)	0.34(0.29-0.40)
85-89	1146 (428-2070)	7.59 (2.83-13.7)	4224 (1647-7527)	9.24 (3.6-16.46)	0.74(0.63-0.86)
90-94	525 (192-962)	12.26 (4.47-22.46)	2948 (1164-5291)	16.48 (6.51-29.57)	1.19(1.07-1.32)
95+	179 (61-365)	17.57 (5.98-35.81)	1505 (541-2831)	27.61 (9.93-51.94)	1.72(1.60-1.85)
SDI regions					
Low SDI	739 (296-1345)	0.42 (0.16-0.74)	1379 (536-2402)	0.35 (0.14-0.61)	-0.55(-0.68–0.43)
Low-middle SDI	1843 (750-3157)	0.38 (0.15-0.64)	4360 (1714-7507)	0.35 (0.14-0.59)	-0.27(-0.33–0.2)
Middle SDI	3865 (1564-6424)	0.49 (0.2-0.81)	11476 (4552-19421)	0.49 (0.19-0.83)	-0.06(-0.12–0.01)
High-middle SDI	2151 (872-3609)	0.26 (0.1-0.43)	5431 (2257-9189)	0.28 (0.12-0.48)	0.2(0.06-0.33)
High SDI	2644 (1065-4515)	0.24 (0.1-0.41)	8165 (3232-13763)	0.33 (0.13-0.55)	1.19(1.09-1.29)

**Table 2:** The number of DALYs and age-standardized DALYs rate of DKD attributable to low physical activity in 1990 and 2021 with EAPC from 1990 to 2021 globally.

Items	1990		2021		1990-2021
	Number of DALYs(95% UI)	The age-standardized DALYs rate/100000(95% UI)	Number of DALYs(95% UI)	The age-standardized DALYs rate/100000(95% UI)	EAPC(95%CI)
Global	297698 (114900-493524)	7.88 (3.07-13.01)	698484 (275039-1158032)	8.19 (3.21-13.6)	0.83(0.55-1.12)
Sex					
Female	162841 (64316-274367)	7.83 (3.11-13.17)	378876 (152287-624534)	8.19 (3.29-13.53)	0.14(0.06-0.21)
Male	134857 (53014-224795)	8.05 (3.12-13.3)	319608 (126803-542012)	8.25 (3.27-13.95)	0.12(0.08-0.17)
Age groups					
25-29	1014 (288-2278)	0.23 (0.07-0.51)	1013 (299-2320)	0.17 (0.05-0.39)	-0.98(-1.04–0.91)
30-34	2401 (688-5035)	0.62 (0.18-1.31)	3358 (970-6873)	0.56 (0.16-1.14)	-0.32(-0.39–0.25)
35-39	5328 (1624-11374)	1.51 (0.46-3.23)	8146 (2650-17243)	1.45 (0.47-3.07)	-0.05(-0.15-0.05)
40-44	8437 (2571-17261)	2.95 (0.9-6.03)	15943 (5489-33307)	3.19 (1.1-6.66)	0.27(0.19-0.36)
45-49	12811 (3857-24874)	5.52 (1.66-10.71)	28270 (9554-53638)	5.97 (2.02-11.33)	0.34(0.28-0.40)
50-54	21028 (7006-39609)	9.89 (3.3-18.63)	48325 (17135-90547)	10.86 (3.85-20.35)	0.35(0.31-0.39)
55-59	31779 (11416-60055)	17.16 (6.16-32.43)	72547 (27425-133097)	18.33 (6.93-33.63)	0.24(0.17-0.32)
60-64	41571 (15901-74882)	25.88 (9.9-46.62)	83759 (33886-154522)	26.17 (10.59-48.28)	0.00(-0.11-0.11)
65-69	44310 (16927-77604)	35.85 (13.69-62.78)	97035 (38429-167471)	35.18 (13.93-60.71)	-0.06(-0.19-0.07)
70-74	42778 (15796-75876)	50.53 (18.66-89.62)	99970 (37010-170762)	48.57 (17.98-82.96)	-0.09(-0.18–0.01)
75-79	38261 (15787-65130)	62.16 (25.65-105.81)	83280 (33639-146486)	63.15 (25.51-111.07)	-0.08(-0.14–0.02)
80-84	26537 (10233-44842)	75.01 (28.93-126.76)	67816 (27278-112035)	77.43 (31.14-127.92)	0.10(0.05-0.14)
85-89	14400 (5564-24984)	95.29 (36.82-165.33)	48357 (18881-83812)	105.76 (41.29-183.31)	0.42(0.33-0.51)
90-94	5540 (2061-9995)	129.29 (48.11-233.24)	28370 (11152-50627)	158.59 (62.34-283)	0.86(0.76-0.96)
95+	1503 (510-3041)	147.61 (50.09-298.68)	12293 (4427-23094)	225.55 (81.22-423.72)	1.62(1.50-1.73)
SDI regions					
Low SDI	19948 (7951-35256)	9.49 (3.79-16.72)	36469 (14099-64593)	7.7 (2.9-13.47)	-0.75(-0.84–0.67)
Low-middle SDI	51335 (19925-87502)	8.97 (3.51-15.11)	116430 (46365-201068)	8.28 (3.25-14.36)	-0.29(-0.36–0.22)
Middle SDI	103094 (40246-173167)	10.84 (4.28-18.05)	269337 (104002-454534)	10.35 (4.04-17.29)	-0.15(-0.22–0.08)
High-middle SDI	57980 (23111-96224)	6.19 (2.48-10.2)	121333 (49711-202985)	6.16 (2.52-10.28)	-0.03(-0.17-0.1)
High SDI	65055 (25196-106565)	5.88 (2.28-9.62)	154325 (64479-252862)	7.17 (2.96-11.76)	0.77(0.68-0.86)

**Figure 1:**
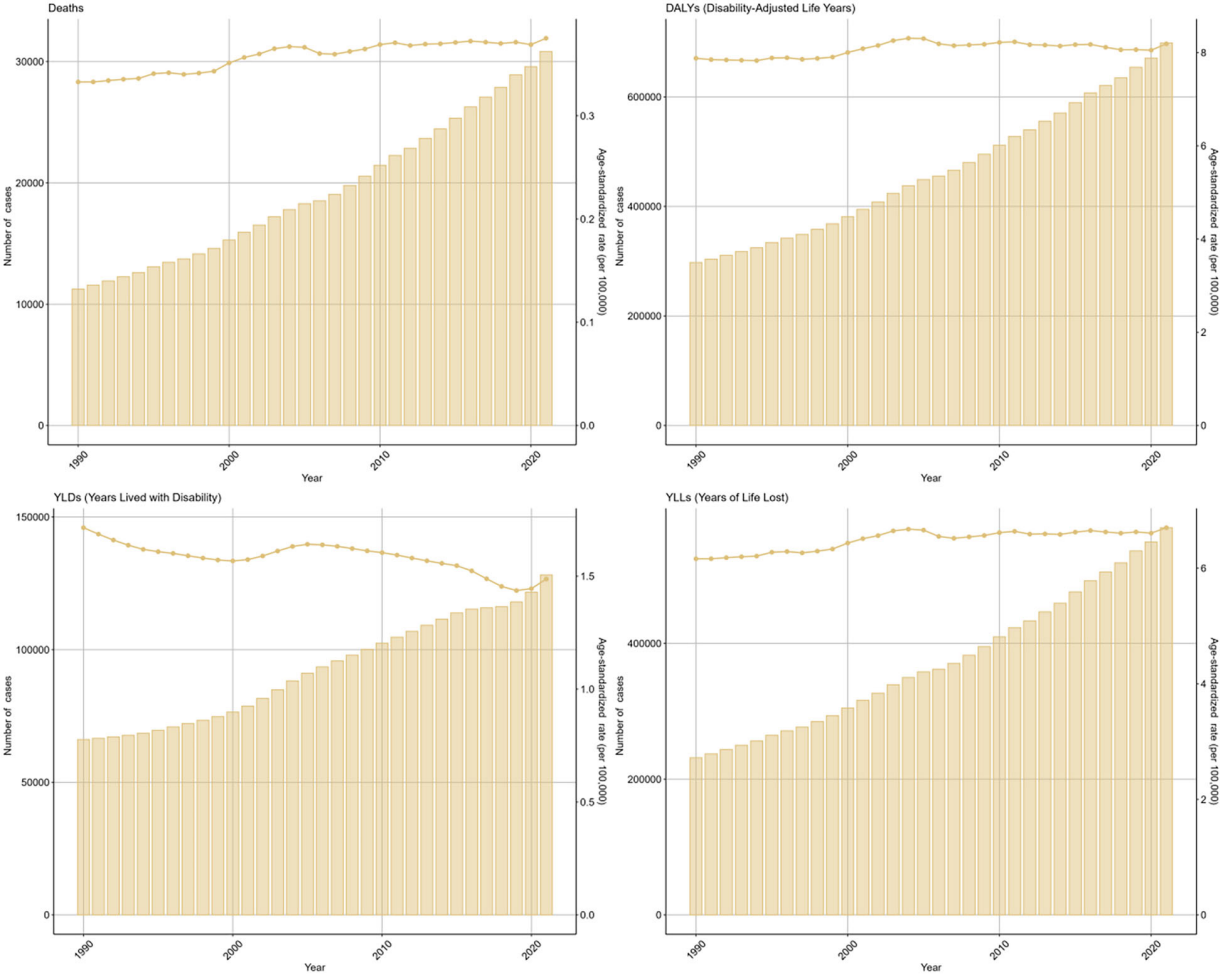
Global trends in numbers (column bar graphs) and age-standardized rates (line charts) of deaths, DALYs, YLDs, and YLLs for DKD attributable to LPA from 1990 to 2019.

As depicted in the bars of **Figure 1**, during 1990 to 2021, there has been a rise in the cases of deaths, DALYs, and their components (YLDs, YLLs) related to the disease. The ASRs of these metrics demonstrated a general upward trend. The EAPC was 1.22 (95%CI: 0.83-1.61) for death, 0.83 (95%CI: 0.55-1.12) for DALYs, 0.33 (95%CI: 0.06-0.60) for YLDs, and 0.96 (95%CI: 0.67-1.25) for YLLs.

### 3.2 Sex disparity of DKD attributable to LPA burden

**Figure 2:**
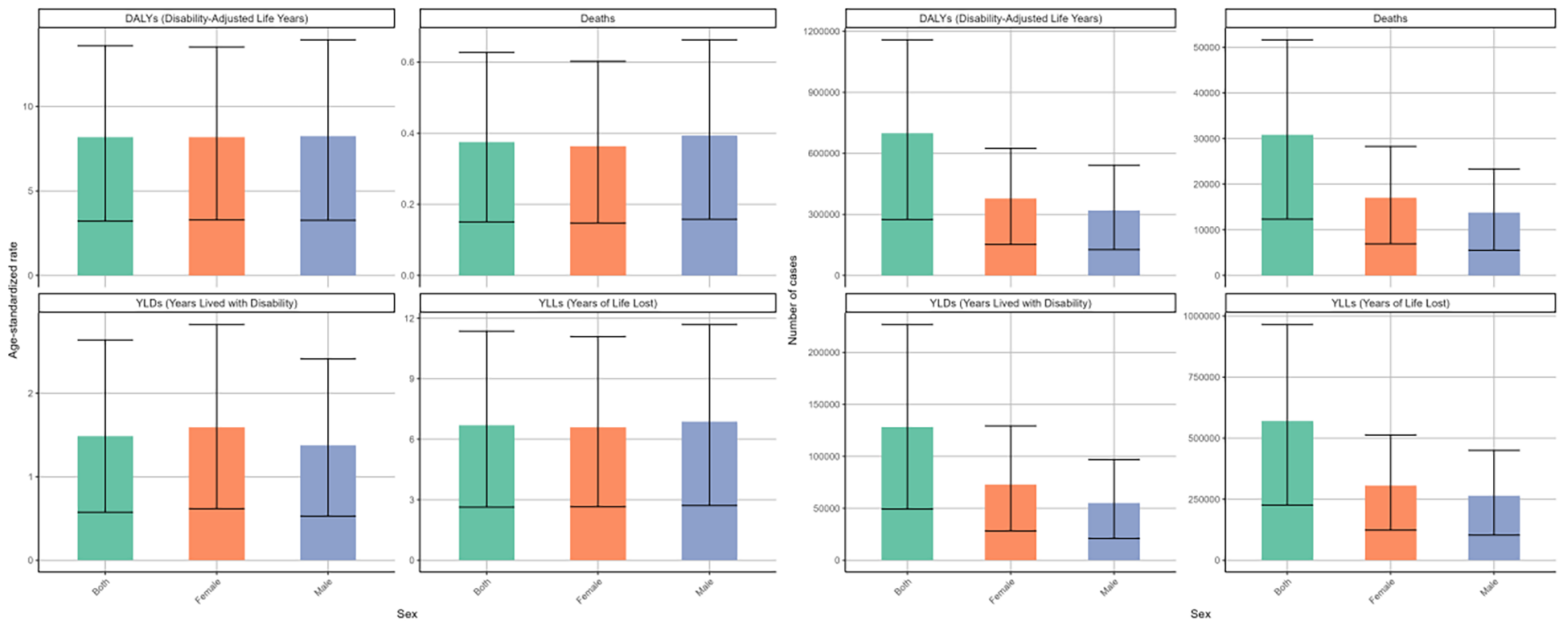
Numbers and age-standardized rates of DKD attributable to LPA DALYs, deaths, YLDs, and YLLs for different genders in 2021.

There were some sex disparities in the DKD attributable to LPA in terms of deaths and disability outcomes (DALYs, YLDs, YLLs), as illustrated in the bar charts for 2021 (**Figure 2**). After factoring in the effects of population growth and demographic aging, it was found that males generally face a greater disease burden than females based on regarding ASRs. Specifically, the age-standardized deaths rate for males was 0.39 (95%UI: 0.16-0.66) per 100000 males and for females at 0.36 (95%UI: 0.15-0.6) per 100000 females. A similar difference was observed in the ASRs of DALYs and YLLs (**Table 2**, **Supplementary Table S2**). However, when considering the raw numbers of these four measures, females had slightly higher counts than males. In 2021, there were 17053 (95%UI: 6929-28269) deaths among females compared to 13783 (95%UI: 5524-23345) among males (**Table 1**, **Figure 2**).

**Figure 3:**
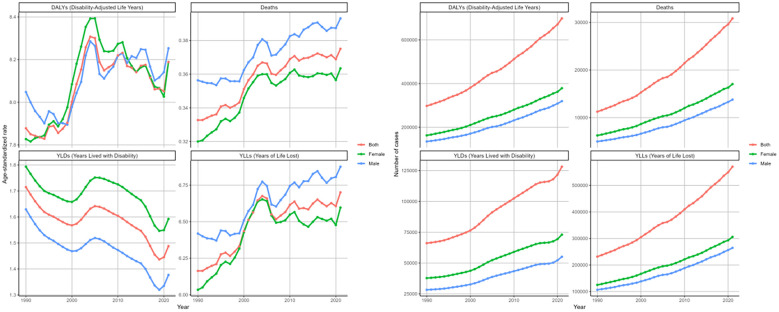
Changing trends in the burden of age-standardized rates and numbers of DKD attributable to LPA DALYs, deaths, YLDs, and YLLs stratified by genders from 1990 to 2021.

From 1990 to 2021, the deaths and disability outcomes numbers demonstrated a steady increase for both sexes, with females consistently reporting higher figures than males. Regarding the DALYs rate, both genders exhibited a fluctuating pattern. Initially, there were fluctuations, with a significant spike around 2000, followed by a decline until around 2015-2020, after which an upward trend emerged again (**Figure 3**). The EPAC was 0.14 (95%CI: 0.06-0.21) for females and 0.12 (95%CI: 0.08-0.17) for males (**Table 2**). The ASR of deaths and YLLs exhibited a generally upward trend with minor variations, while the age-standardized YLDs rate presented a downward trend. For females, the EPAC was -0.25 (95%CI: -0.36–0.14) and for males was -0.47 (95%CI: -0.56–0.38). (**Supplementary Table S1**; **Figure 3**). This evidence underscores a significant worsening of the overall disease burden for both sexes.

### 3.3 DKD attributable to LPA burden in age groups

**Figure 4:**
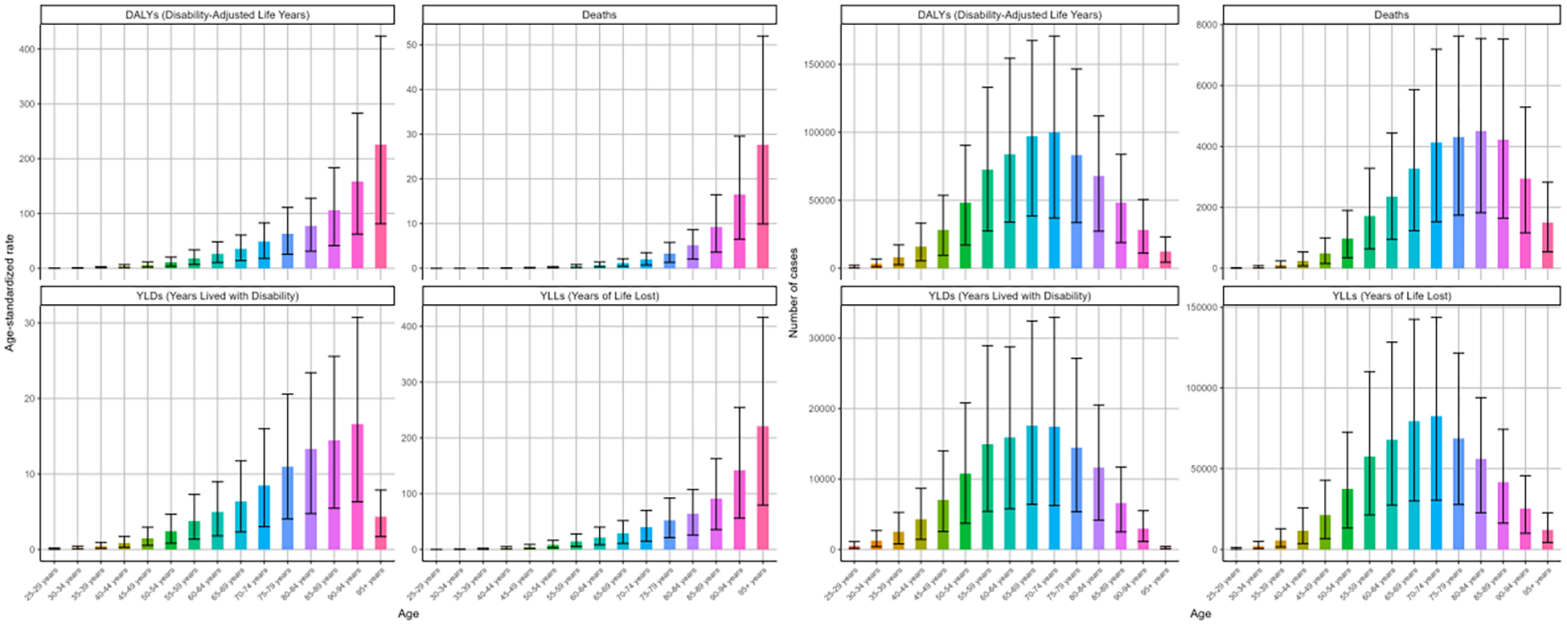
Age-standardized rates and numbers of DKD attributable to LPA DALYs, deaths, YLDs, and YLLs stratified by age groups in 2021.

In 2021, the cases and ASRs of deaths and DALYs and its components across different age groups were analyzed (**Figure 4**.). The ASRs of four metrics rose steadily with age, except for YLDs in the 95+ group. Here, the ASR of YLDs dropped to 4.36 (95%UI: 1.7-7.88) per 100,000 population, showing a decline relative to other age groups(**Supplementary Table S1**). The numbers of these measures initially rose with age, peaking in the 70–74 category, then decreased. For the number of deaths, however, a more prominent peak was noted in 80–84 age stratum with 4508 (95%UI: 1824-7544) death cases (**Table 1**).

**Figure 5:**
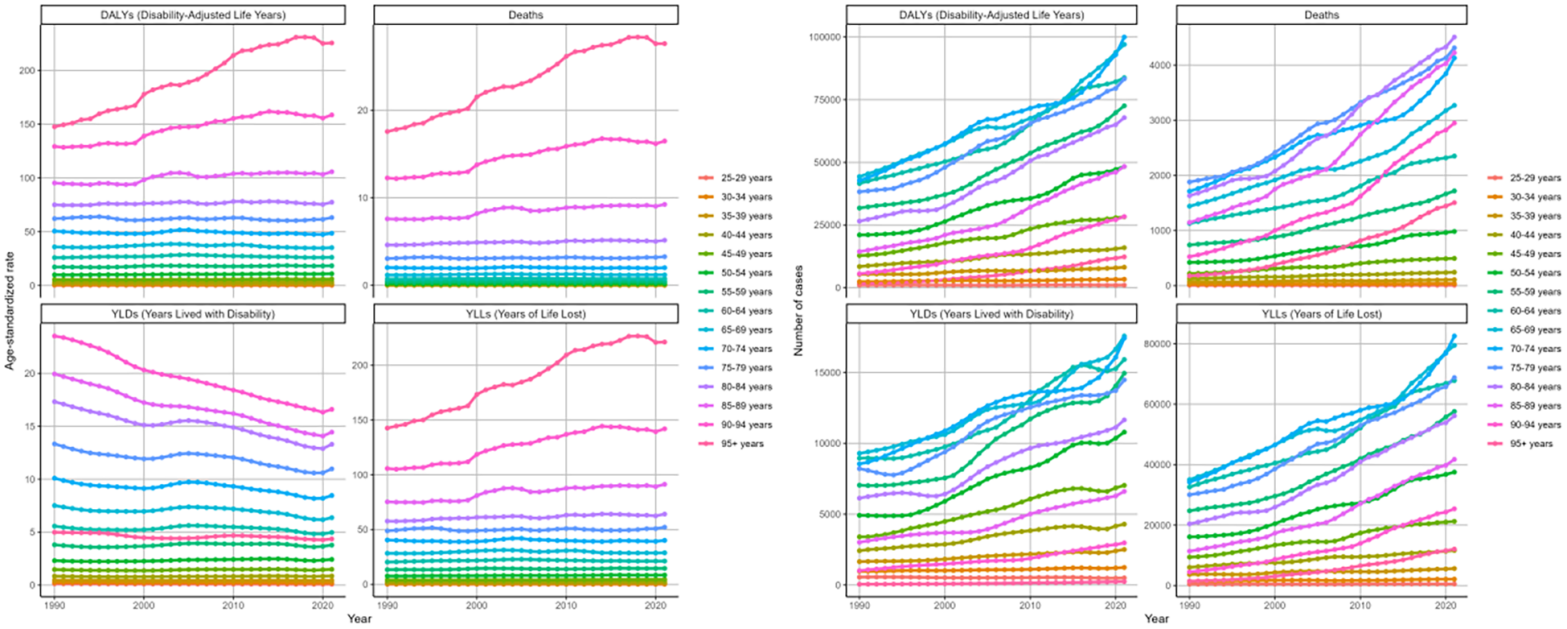
Changing trends in the burden of age-standardized rates and numbers of DKD attributable to LPA DALYs, deaths, YLDs, and YLLs stratified by age groups from 1990 to 2021.

Based on the analysis of changing trends across age cohorts over three decades. The ASRs of deaths, DALYs, and its components for age groups above 40 years showed an increasing trend (**Figure 5**). We observed the greatest rise in the group aged 95 and above, which showed an EPAC of 1.72 (95%CI: 1.60-1.85) for death, 1.62 (95%CI: 1.50-1.73) for DALYs, with 1.67 (95%CI: 1.55-1.79) for YLLs (**Tables 1**, **2**, **Supplementary Tables S1**, **S2**). Conversely, YLD age-standardized rates consistently decreased in the 60+ demographic cohort. The most significant decrease was in the 90–94 years group, with an EAPC of -1.20 (95%CI: -1.24–1.15). The numbers of the four indicators showed increasing trends in most age groups (**Tables 1**, **2**, **Supplementary Tables S1**, **S2**, **Figure 5**).

### 3.4 DKD attributable to LPA burden stratified by SDI quintiles

**Figure 6:**
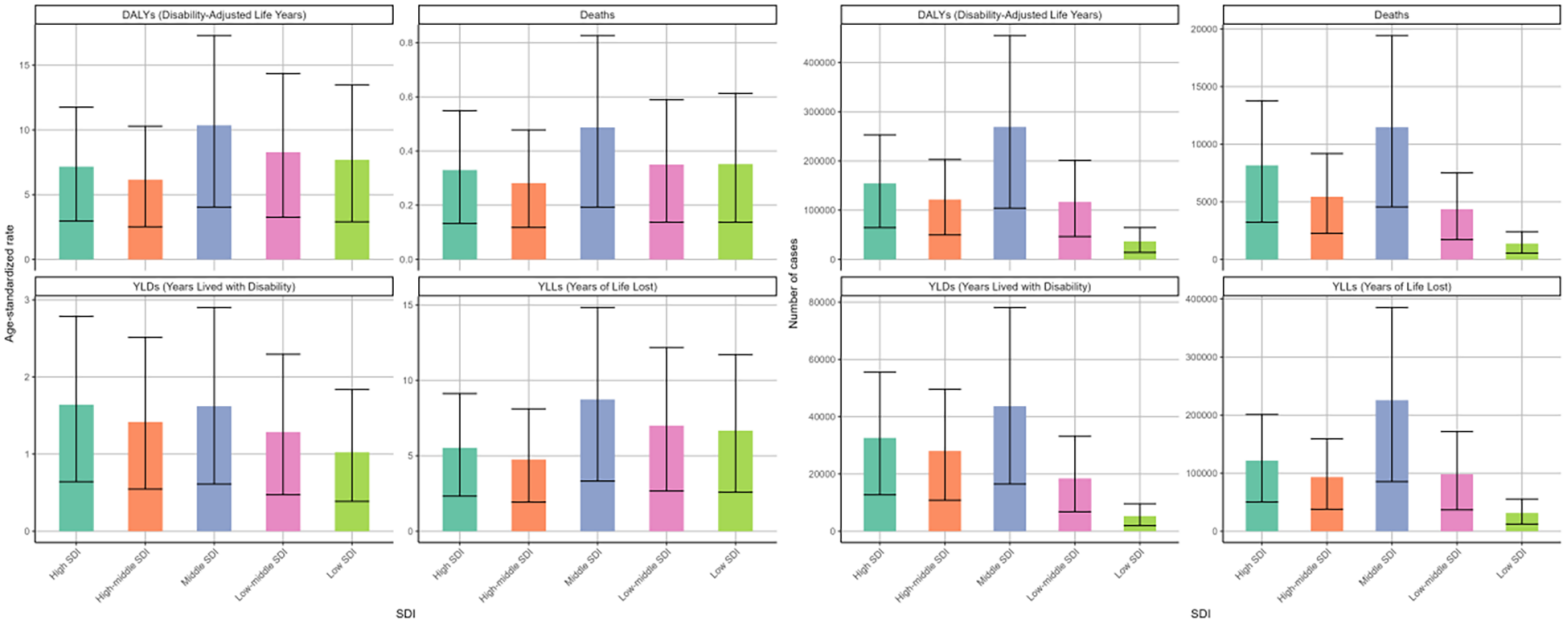
Age-standardized rates and numbers of DKD attributable to LPA DALYs, deaths, YLDs, and YLLs in different SDI quintiles in 2021.

In 2021, the burden of DKD due to LPA stratified by SDI quintiles showed distinct differences. The middle SDI region exhibited the highest disease burden. Specifically, the figures for deaths, DALYs, and corresponding age-standardized rates were 11476 (95%UI: 4552-19421), 269337 (95%UI: 104002-454534), 0.49 (95%UI: 0.19-0.83) per 100000 people, and 10.35 (95%UI: 4.04-17.29) per 100000 people, respectively (**Figure 6**, **Tables 1**, **2**). In the cases of four measures, high-middle and high SDI regions followed closely behind. However, these two regions showed relatively lower ASRs of deaths, DAYLs, and YLLs compared to others (**Figure 6**).

**Figure 7:**
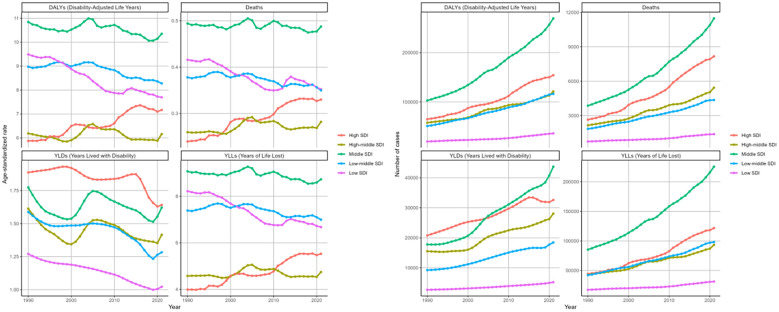
Changing trends in the burden of age-standardized rates and numbers of DKD attributable to LPA DALYs, deaths, YLDs, and YLLs in different SDI regions from 1990 to 2021.

Between 1990 and 2021, the high SDI region experienced the most notable surge in age-standardized death rates among the five SDI regions, displaying an EAPC of 1.19 (95%CI: 1.09-1.29), followed by high-middle SDI with an EAPC of 0.2 (95%CI: 0.06-0.33) (**Table 1**, **Figure 7**). In contrast, the other SDI regions exhibited declining trends in the age-standardized mortality rate. The ASRs of DALYs and YLLs showed a consistent distribution pattern (**Table 2**, **Supplementary Table S2**). However, analysis of age-standardized YLDs rates revealed consistent increases across all SDI quintiles over three decades, most dramatically in middle SDI populations (**Figure 7**, **Supplementary Table S1**).

### 3.5 DKD attributable to LPA burden stratified by geographical location

The disease burden of DKD attributable to LPA in 2021, along with its trend, was compared across 54 GBD regions and 204 nations and territories.

**Figure 8:**
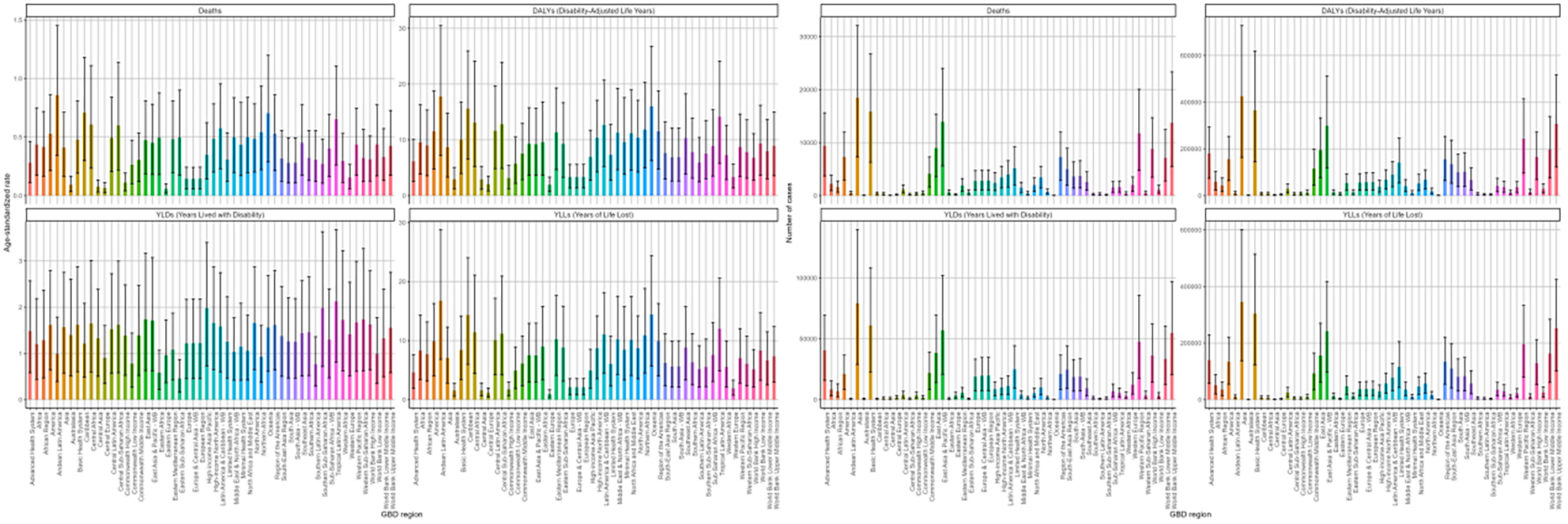
Age-standardized rates and numbers of DKD attributable to LPA deaths Deaths, DALYs, YLDs, and YLLs in 2021 stratified by 54 GBD regions.

In 2021, Andean Latin America possessed the largest ASRs for deaths, DALYs, and YLLs, reaching 0.86/100000 (95%UI: 0.34-1.45), 17.77/100000 (95%UI: 7.19-30.48), and 16.78/100000 (95%UI: 6.85-28.78), respectively, across 54 GBD regions. Conversely, Eastern Europe had the lowest health burden, with ASRs of 0.06/100000 (95%UI: 0.02-0.1) for deaths, 1.93/100000 (95%UI: 0.71-3.31) for DALYs, and 0.97/100000 (95%UI: 0.36-1.66) for YLLs (**Supplementary Tables S3**–**S6**). Asia reported the highest figures for deaths and DALYs, with 18,459 (95% UI: 7,271-32,091) and 425,214 (95% UI: 163,523-727,831), respectively. followed by Basic Health System. Similar disparities were also evident in the number of YLDs and YLLs for the disease (**Supplementary Tables S3**–**S6**, **Figure 8**).

**Figure 9:**
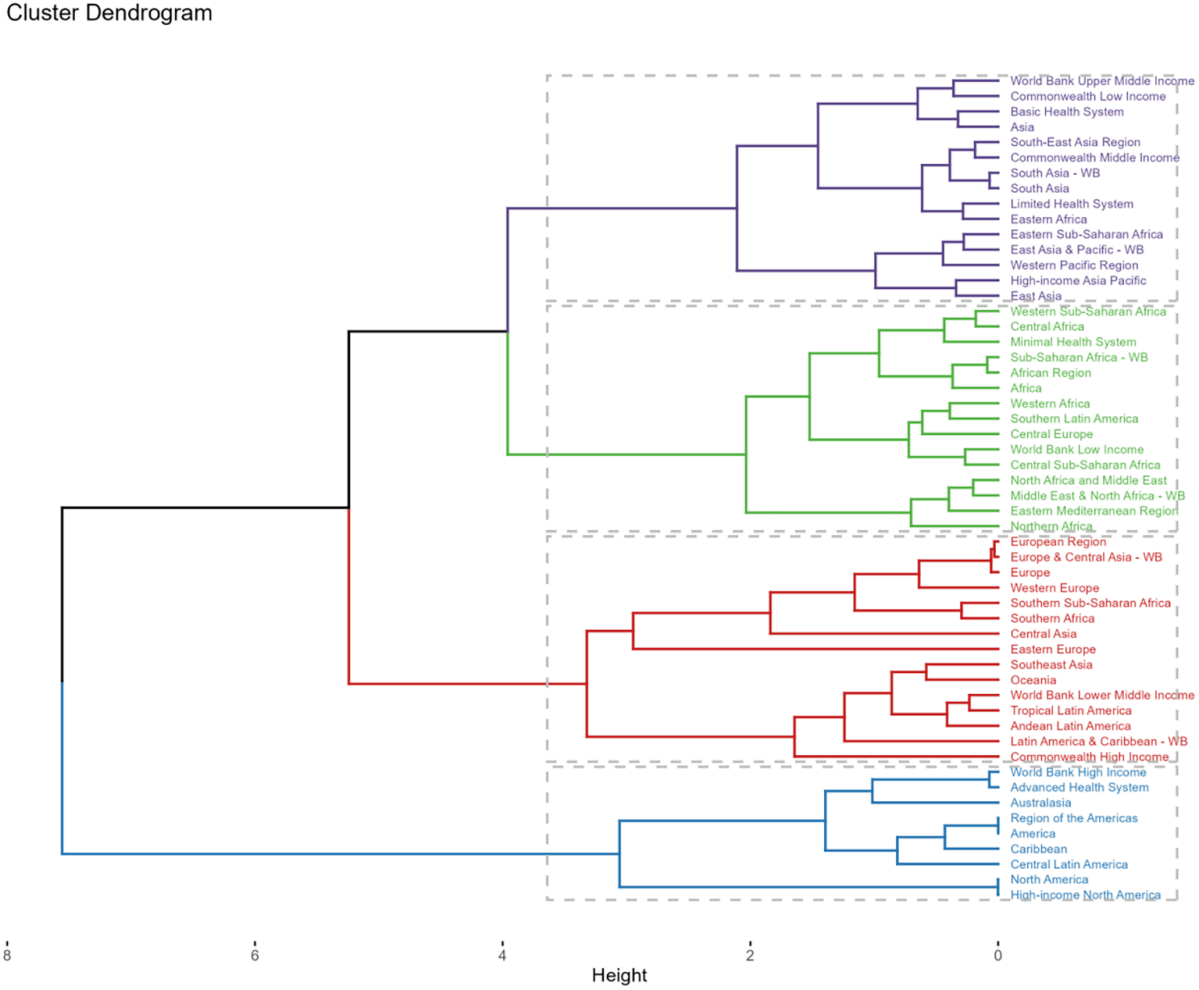
Subalternative findings of cluster analysis derived from the EAPC values of the DKD attributable to LPA age-standardized rates for deaths and DALYs stratified by 54 GBD regions during 1990 - 2021.

Between 1990 and 2021, the majority of 54 GBD regions exhibited a rising pattern in ASRs of deaths and DALYs. High-income North America demonstrated the pronounced escalations in both metrics with EAPCs of 3.16 (95%CI: 2.51-3.81) and 2.53 (95%CI: 1.97-3.09) per 100,000 individuals, respectively. Conversely, Southern Sub-Saharan Africa experienced a decline in the ASRs of deaths and DALYs (**Supplementary Tables S3**, **S4**). After a comprehensive analysis of the EAPC of all indicators and cluster analysis, it was determined that DKD caused by LPA burden increased slightly in 15 areas like World Bank Upper Middle Income regions. In 15 regions, including Africa, the burden remained stable or decreased slightly. A significant decrease was observed in 15 regions, notably Southern Sub-Saharan Africa, while a significant increase occurred in 9 regions, including certain North American regions (**Figure 9**).

**Figure 10:**
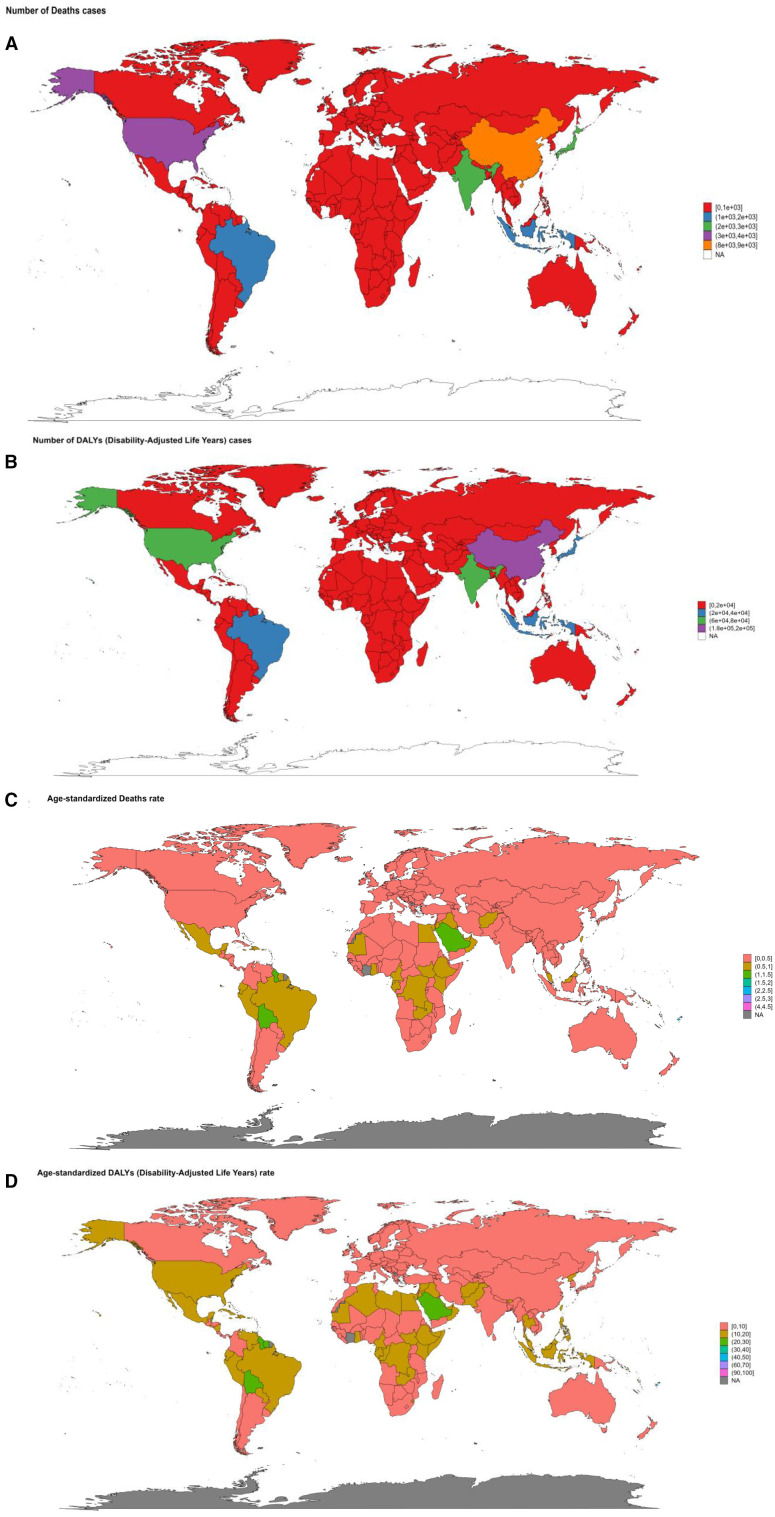
The summary map of DKD attributable to LPA burden in 2021 stratified by 204 countries and territories. **(A)** number of death cases. **(B)** number of DALYs cases. **(C)** age-standardized deaths rate. **(D)** age-standardized DALYs rate.

In 2021, China reported the greatest death cases from DKD attributable to LPA, with 8453 (95%UI: 3279-14480) cases, followed by the United States of America with 3323 (95%UI: 1393-5372) cases and India (2844 (95%UI: 1088-5036)) cases in 204 countries (**Supplementary Tables S3**). A large number of countries, predominantly covering most of Africa, extensive parts of Europe, Australia, and various other dispersed regions worldwide, had a relatively low number of death cases, ranging from 0 to 1000 (**Figure 10A**). The distribution of DALYs, YLDs, YLLs across countries showed a consistent pattern (**Supplementary Tables S3**, **S6**, **Figure 10B**, **Supplementary Figure S1**). American Samoa (4.36/100000 (95%UI: 1.78-7.46)) and Micronesia (Federated States of) (2.93/100000 (95%UI: 1.04-5.15)) had the highest ASR of death in 2021. Similar results can also be found in the ASR of DALYs and its components (**Supplementary Tables S3**–**S6**, **Figures 10C, D**, **Supplementary Figure S1**).

**Figure 11:**
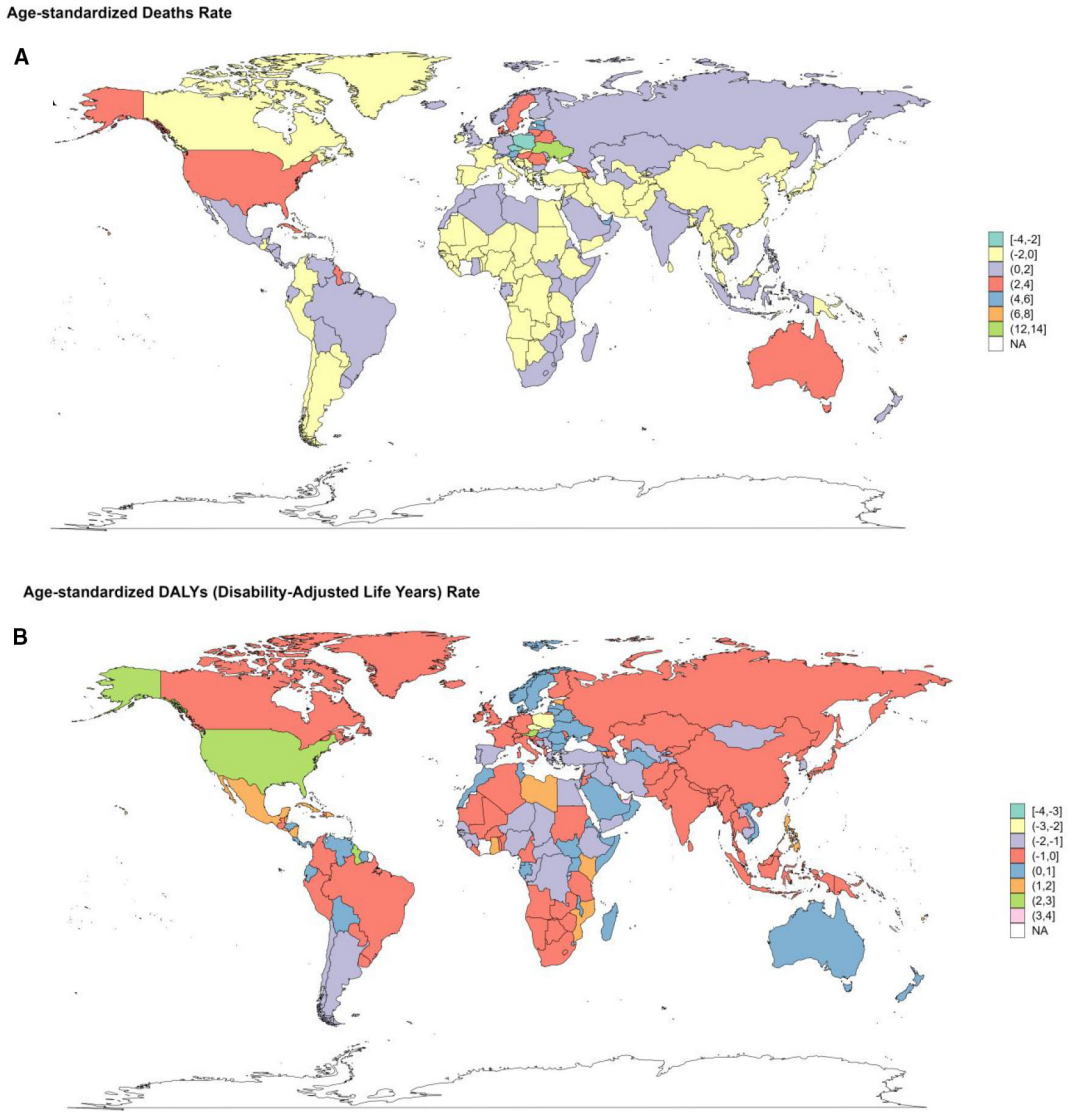
Changes of EAPC in the burden of DKD attributable to LPA stratified by countries from 1990 to 2021. **(A)** changes in death. **(B)** changes in DALYs.

From 1990 to 2021, concerning ASRs, Poland and Czechia exhibited a downward trend of deaths and YLLs. Poland experienced the most pronounced decrease in the death rate, with an EAPC of -2.28 (95%CI: -3.01–1.54) and -2.68 (95%CI: -3.4–1.95) in the YLLs rate. Luxembourg also showed a remarkable decline, with substantial drops in the EAPC of multiple metrics. The EAPC for the age-standardized deaths rate was -2.37 (95%CI: -3.09–1.65), for DALYs rate was -2.78 (95%CI: 3.4–2.16), for YLDs rate was -3.08 (95%CI: -3.61–2.54), and for YLLs rate (-2.59 (95%CI: -3.28–1.9) (**Supplementary Tables S3**–**S6**. **Figure 11**, **Supplementary Figure S2**). Conversely, Ukraine and Armenia witnessed significant increases. Ukraine had the most considerable growth in the death rate with an EPAC of 13.24 (95%CI: 11.74-14.75) and 13.72 (95%CI: 12.16-15.3) for YLLs rate. Armenia followed, with increases in the death rate (EAPC: 95%CI: 8.22 (7.4-9.05)) and YLLs rate (EAPC: 95%CI: 7.86 (6.99-8.74)). American Samoa and Mauritius showed a relatively significant rise in the age-standardized DALYs rate, with EPACs of 4.58 (95%CI: 3.82-5.35) and 4.22 (95%CI: 3.73-4.71), respectively. Additionally, Mauritius had an upward trend in the YLDs rate (EAPC: 2.59 (95%CI: 2.23-2.95)), trailed by the Republic of Korea, Singapore, and Japan (**Supplementary Tables S3**–**S6**. **Figure 11**, **Supplementary Figure S2**).

### 3.6 Health inequality analysis by socioeconomic development level

**Figure 12:**
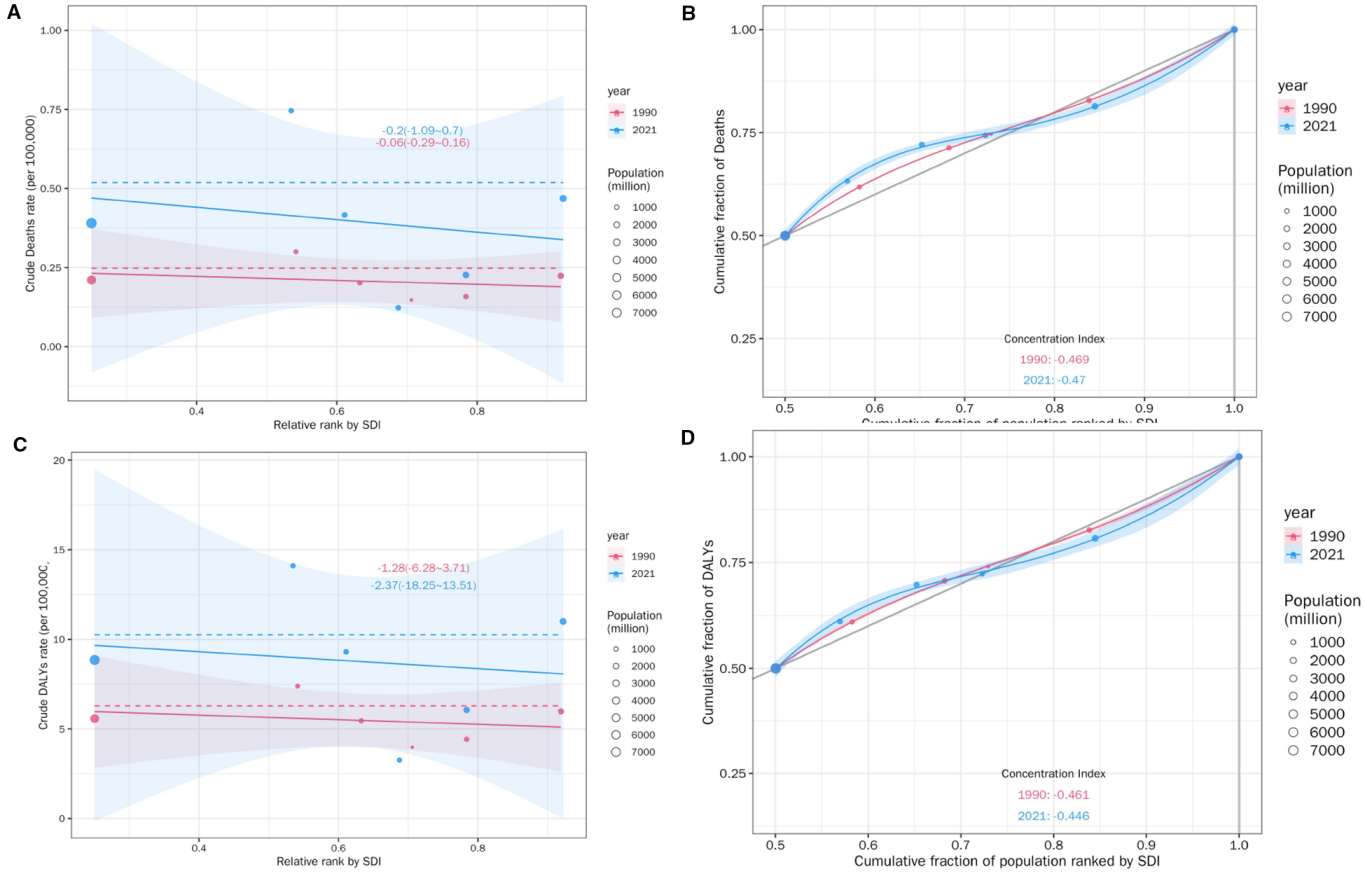
SDI-related health inequality regression **(A)** and concentration **(B)** curves for the Deaths of DKD attributable to LPA, 1990 and 2021. SDI-related health inequality regression **(C)** and concentration **(D)** curves for the DALYs of DKD attributable to LPA, 1990 and 2021.

Significant absolute and relative inequalities in DKD attributable to LPA.

burden were observed across socioeconomic development level (**Figure 12**). As demonstrated by the slope index of inequality, the crude death rate exhibited a negative correlation with the relative SDI rank; there was a decrease of -0.06 (95% CI: -0.29-0.16) deaths per 100,000 across globe and five SDI regions in 1990, which showed a downward trend of -0.2 (95% CI: -1.09-0.7) in 2021 as the SDI rank increased (**Figure 12A**), though the absolute death rates in 2021 were generally higher.

than in 1990 for most SDI ranks. The cumulative fraction of deaths by SDI-ranked population further provided relative gradient inequality in disease mortality burden (**Figure 12B**), In 2021, the cumulative distribution of deaths showed a steeper increase at lower SDI ranks compared to 1990, indicating a greater concentration of mortality burden in populations with relatively lower sociodemographic development over time.

In summary, the concentration index, a measure of relative inequality, reinforced this trend, with values of -0.469 in 1990 and -0.47 in 2021. These findings indicated that the mortality burden of DKD attributable to LPA was disproportionately concentrated in populations with lower SDI. More importantly, there was a marginal worsening of mortality burden inequality by SDI from 1990 to 2021. Similar health disparities were observed for DALYs (**Figures 12C, D**),YLDs and YLLs (**Supplementary Figure S3**).

### 3.7 Predictions of the disease burden of DKD attributable to LPA

**Figure 13:**
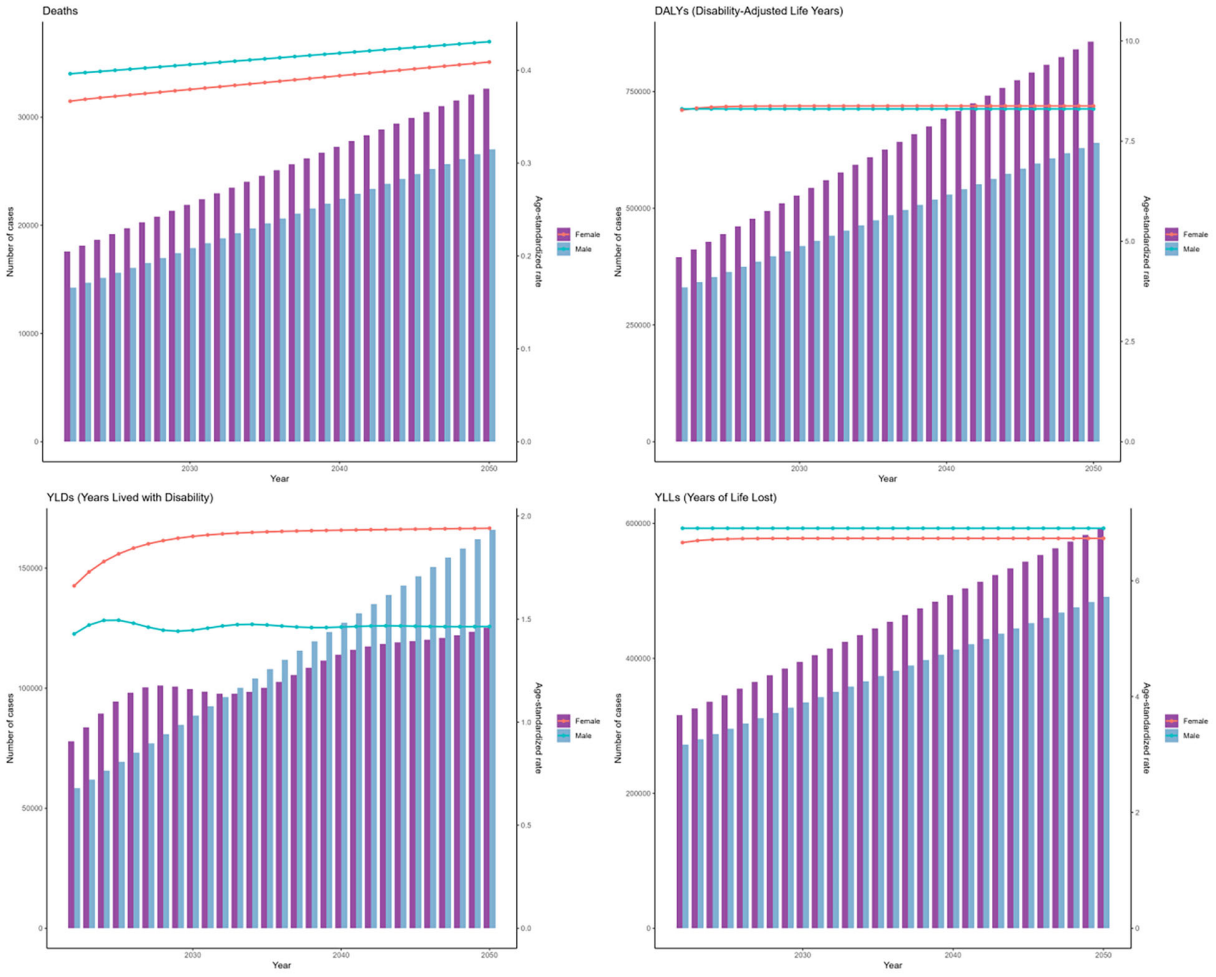
The prediction in numbers (column bar graphs) and age-standardized rates (line charts) of deaths, DALYs, YLDs, and YLLs for DKD attributable to LPA burden for females and males from 2021 to 2050 by the ARIMA model.

**Figure 14:**
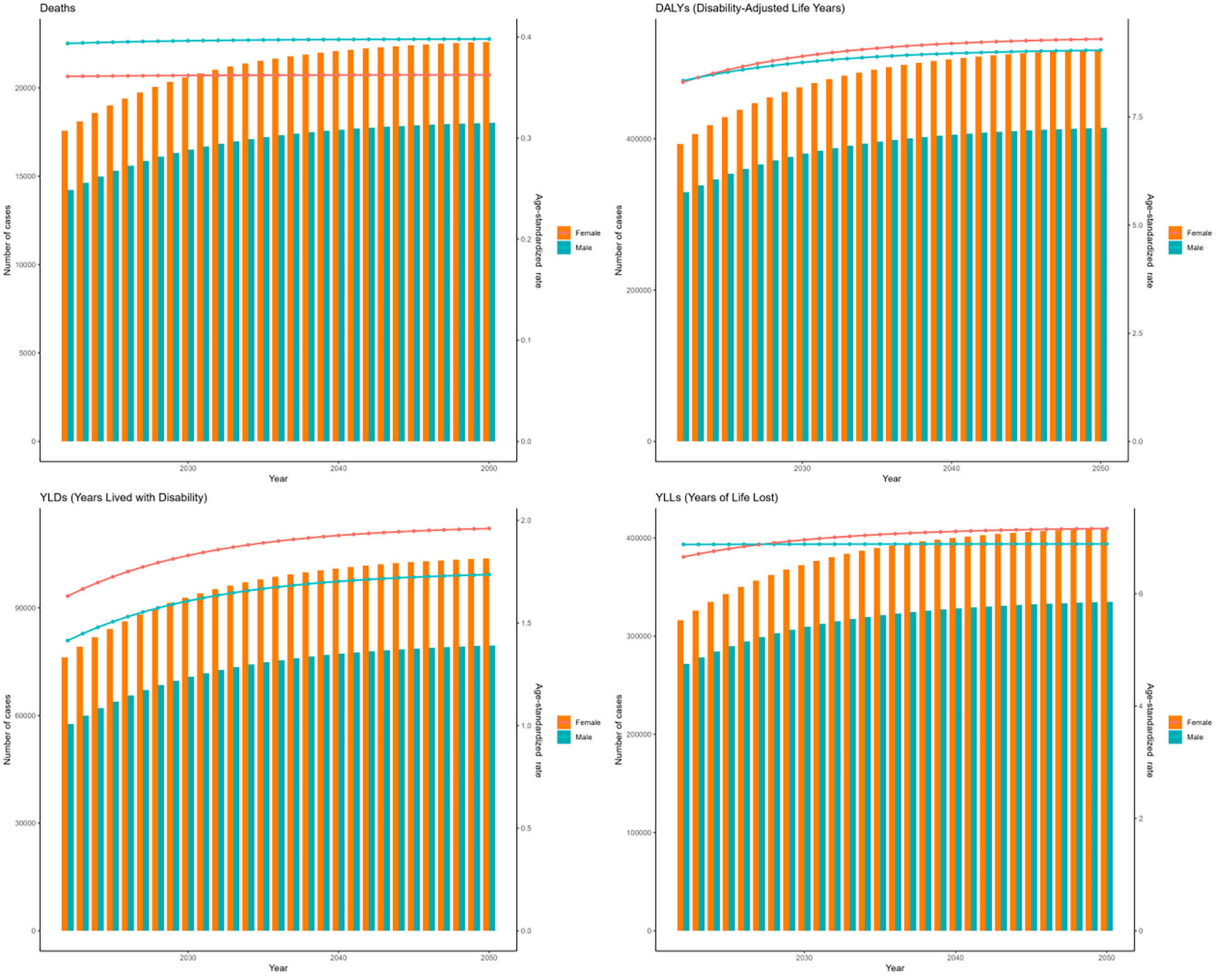
The prediction in numbers (column bar graphs) and age-standardized rates (line charts) of deaths, DALYs, YLDs, and YLLs for DKD attributable to LPA burden for females and males from 2021 to 2050 by the ES model.

The forecasts derived from the ARIMA and ES models suggest an upward trend in the cases of deaths, DALYs, YLDs, and YLLs associated with DKD due to LPA for both genders globally from 2021 to 2050. (**Figures 13**, **14**). Moreover, both models forecast a slight increase or stable trends in the ASRs of these four metrics for both males and females. Specifically, the ARIMA model forecasts a rise in death cases to 32623 for females and 27035 for males, with DALYs projected to reach 856848 for females and 640159 for males (**Figure 13**). Meanwhile, the ES model forecasts an increase in mortality cases to 22598 among females and 18029 among males, and the number of DALYs will increase to 517922 in females and 414402 in males (**Figure 14**).

## 4 Discussion

Our study is the first to evaluate and quantify the global burden of T2DM-related DKD caused by LPA across regions and time. And further provides a systematic evaluation integrating trend, health inequality, and predictive analyses. The substantial increase in deaths related to DKD attributable to LPA is alarming, with a staggering rise of 174.02% to reach a total of 30.84 thousand cases over 30 years in the study. Moreover, the number and all ASRs of the disease remained extremely grave, which showed an upward trend. Additionally, our projected outcomes indicated that the disease impact will persist in escalating over the next 25 years; this indicates that the impact of LPA on DKD is becoming more prominent, and the disease burden is escalating, presenting a severe challenge to public health.

The WHO aims to reduce physical inactivity levels by 10% by 2025, thereby contributing to the decrease of non-communicable disease burden (29). However, DKD due to LPA significantly influences the global disease burden. Physical inactivity is claimed to account for 6.9% of T2DM burden (30). A large population-based study of 2,381 adults with DM found that meeting WHO physical activity guidelines was associated with a reduced risk of CKD (OR = 0.55), an effect driven primarily by leisure-time activity (>600 METs/week), while work-related physical activity showed no significant association (31). This finding is further supported by a large-scale meta-analysis (32). LPA is also linked to negative clinical outcomes and diminished quality of life in kidney disease (33). CKD patients have double the risk of sarcopenia than those without CKD, and appropriate physical activity remains the primary treatment of sarcopenia (34). This highlights the adverse impacts of LPA on the onset and progression of DKD (35).

Our study revealed gender disparities in the DKD burden attributable to LPA. Males had a higher disease burden. In GBD 2019 study, the total disease burden caused by LPA showed 205.53/100000 (95%UI: 102.86-388.82) for males vs 190.92/100000 (95%UI: 109.35-324.61) for females in the ASRs of DALYs in 2019, and the same trend in death (30). The higher burden in males may be ascribed to heightened exposure to T2DM risk factors, including diminished exercise, higher cigarette and alcohol intake, and augmented daily calorie intake (36). Men might have limited time and lower motivation to engage in exercise at rest. In comparison, females may stay active through household chores and childcare and also have more leisure time for dedicated exercise (37). Conversely, females who had higher absolute case numbers might result from their extended longevity with progressive susceptibility to age-dependent morbidity factors (38). Besides, Sergi. et al. identified and revealed that under high glucose conditions in kidney, males exhibited greater mitochondrial dysfunction, higher apoptosis and injury compared with females on gender-based discrepancies in the metabolism and blood metabolome patterns (39).

Age-stratified analysis showed that the disease burden increased in age groups above 40 years, with the 70 + years population bearing the highest burden. This could be due to several elements, such as the following: In China, the diabetes prevalence is 11.1% in 40-49-year-olds and rises to 27.3% in those aged 70 years or older (40). Notably, the prevalence of diabetic kidney complications increased with aging (41). The elderly are more vulnerable to LPA, which is consistent with the global aging population and the cumulative effects of LPA on renal function over time (42). Older individuals suffer from significant musculoskeletal disorders (43). This could negatively impact physical activity levels and possibly elevate sedentary behavior (44). LPA further exacerbates metabolic disorders and inflammatory responses, promoting the development of DKD (45).

Striking differences in the DKD attributable to LPA burden also existed across regions and countries (5, 46). In the LPA related-overall deaths investigation, it found that the middle countries also had great age-standardized DALYs and death rate burdens (30), which is consistent with our research. However, the high SDI region had the most significant upward trend in the age-standardized death rate of DKD due to LPA. This may be in high SDI regions; although medical resources are abundant, the severe aging of the population and changes in lifestyle (such as increased sedentary time) may lead to an increase in the age-standardized death rate in various diseases (47–49). Earlier research suggests that inactivity is more prevalent in low-income and less-educated groups (50, 51). However, our study showed declining ASRs in low SDI regions, possibly due to lower baseline prevalence and reduced access to healthcare leading to underdiagnosis (52). Less-developed regions may lack effective disease prevention and early screening systems; limited access to quality healthcare exacerbates disease burden in underserved populations.

Geographically, the Andean Latin America region had the highest ASRs, while the Eastern Europe region had the lowest, which is in accordance with our SDI subtype findings. Countries like China, the United States, and India reported disproportionately high mortality rates. These three countries exhibited substantial populations affected by physical inactivity, likely attributable to their demographic scale (53). These differences are closely related to variations in economic development levels, lifestyles, medical and health conditions, and disease prevention and control measures in each region (54). The 1980s marked a pivotal nutritional transition in the United States, characterized by simultaneous deterioration in dietary quality, declines in physical activity, and accelerating obesity rates, which subsequently emerged across European populations (47). Future initiatives must prioritize geotailored intervention frameworks for these high-risk regions, optimizing both primary diabetes prevention and secondary DN management to mitigate disproportionate nephropathic burden.

The evaluation of health inequalities of DKD due to LPA across SDI gradients displayed a greater concentration of mortality burden in populations with relatively lower sociodemographic development, which also could guide where DKD due to LPA preventive interventions and control efforts should be enhanced. According to a Brazil survey in 2009, a total of 85.3% of adults did not achieve the recommended standards for leisure-time physical activity (55). A study of a low socioeconomic region found that 68.8% of adults did not engage in even 10 minutes of weekly leisure-time physical activity (56). Conventionally, it is presumed that high-SDI nations afford their residents better access to healthcare and more robust healthcare system performance, which in turn would be anticipated to reduce the disease burden associated with DKD due to LPA. However, the disease burden remains high even in high SDI regions. Population aging, along with the disregard for physical activity, accounts for the substantial disease burden observed in high-SDI regions. Higher-SDI countries should develop evidence-based strategies for early diagnosis and management of DKD attributable to LPA while addressing aging populations. Lower-SDI countries require international support to combat healthcare constraints and population growth. The significant rise in inequalities highlights the urgent need for increased investment in prevention and treatment of DKD attributable to LPA alongside sociodemographic development.

This study is the first to combine two advanced models to predict the trend of the DKD due to LPA burden between 2021 and 2050. The results indicate a persistent upward trend in the global disease burden of DKD associated LPA. This prediction emphasizes the urgency of implementing effective intervention measures to curb the further disease burden. As LPA is a modifiable determinant of disease risk, targeted strategies to promote physical activity could significantly reduce DKD incidence and progression, particularly in high-risk groups such as older adults and males. Integrating structured exercise programs into DKD care, alongside pharmacological treatments, could significantly improve outcomes through tailored regimens and community initiatives. Changes like sedentary lifestyles and workplace interventions are needed to meet WHO activity targets, especially in aging high-SDI populations and genders. At the same time, mobile health solutions could address disparities in low-resource settings. These multidisciplinary efforts in clinical care, guidelines, and community engagement are vital to curb the rising DKD burden and align with global goals for active living and health equity.

These findings should be interpreted within study constraints. The reliance on GBD data may introduce biases due to variations in data quality and reporting across regions. The standardized classification of GBD regions fails to capture critical differences in social determinants of health. Additionally, the definition of LPA based on GBD 2021 criteria was technically constrained from conducting specific analyses, like varying activity type and different METs cutoff values, which could better capture PA and DKD correlation. In future research, we will incorporate these details into our analysis. Consistent with the GBD study’s framework, our analysis focused on adults aged ≥25 years, which may limit the generalizability of our findings to the younger population. Despite these limitations, the study offers distinct merits. Rigorous, multi-dimensional investigations (including descriptive, trend, health inequality, and predictive analyses) have enhanced our insights into the epidemiological characteristics of DKD due to LPA. Our findings provide a foundation for policymakers and healthcare providers to develop evidence-based strategies to reduce LPA and improve outcomes for individuals at risk of DKD.

## 5 Conclusion

The global burden of DKD attributable to LPA is substantial and increasing, with significant disparities across populations and regions. Future studies should prioritize three key areas: (1) geographic disparities in LPA-associated DKD burden, (2) intervention efficacy for the disease reduction, and (3) incorporating physical activity initiatives into renal health policies.

## Data Availability

The datasets presented in this study can be found in online repositories. The names of the repository/repositories and accession number(s) can be found below: http://ghdx.healthdata.org/gbd-results-tool.

## References

[1] Doshi SM Friedman AN. Diagnosis and management of type 2 diabetic kidney disease. Clin J Am Soc Nephrol. (2017) 12:1366–73. doi: 10.2215/CJN.11111016, PMID: 28280116 PMC5544517

[2] Thomas MC Brownlee M Susztak K Sharma K Jandeleit-Dahm KA Zoungas S. Diabetic kidney disease. Nat Rev Dis Primers. (2015) 1:15018. doi: 10.1038/nrdp.2015.18, PMID: 27188921 PMC7724636

[3] Li J Guo K Qiu J Xue S Pi L Li X. Epidemiological status, development trends, and risk factors of disability-adjusted life years due to diabetic kidney disease: A systematic analysis of Global Burden of Disease Study 2021. Chin Med J (Engl). (2025) 138:568–78. doi: 10.1097/CM9.0000000000003428, PMID: 39863522 PMC11882292

[4] Deng Y Li N Wu Y Wang M Yang S Zheng Y. Global, regional, and national burden of diabetes-related chronic kidney disease from 1990 to 2019. Front Endocrinol (Lausanne). (2021) 12:672350. doi: 10.3389/fendo.2021.672350, PMID: 34276558 PMC8281340

[5] Alicic RZ Rooney MT Tuttle KR. Diabetic kidney disease: challenges, progress, and possibilities. Clin J Am Soc Nephrol. (2017) 12:2032–45. doi: 10.2215/CJN.11491116, PMID: 28522654 PMC5718284

[6] Thomas B. The global burden of diabetic kidney disease: time trends and gender gaps. Curr Diabetes Rep. (2019) 19:18. doi: 10.1007/s11892-019-1133-6, PMID: 30826889

[7] Foster C Shilton T Westerman L Varney J Bull F. World Health Organisation to develop global action plan to promote physical activity: time for action. Br J Sports Med. (2018) 52:484–85. doi: 10.1136/bjsports-2017-098070, PMID: 28724712

[8] Kaze AD Santhanam P Ahima RS Bertoni AG Echouffo-Tcheugui JB. Association between microvascular disease and cardiorespiratory fitness among adults with type 2 diabetes. Diabetes Care. (2024) 47:1408–14. doi: 10.2337/dc24-0294, PMID: 38837904 PMC11272972

[9] Shi K Zhu Y Lv J Sun D Pei P Du H. Association of physical activity with risk of chronic kidney disease in China: A population-based cohort study. J Sport Health Sci. (2024) 13:204–11. doi: 10.1016/j.jshs.2023.07.004, PMID: 37532222 PMC10980896

[10] Roshanravan B Gamboa J Wilund K. Exercise and CKD: skeletal muscle dysfunction and practical application of exercise to prevent and treat physical impairments in CKD. Am J Kidney Dis. (2017) 69:837–52. doi: 10.1053/j.ajkd.2017.01.051, PMID: 28427790 PMC5441955

[11] Pongrac Barlovic D Tikkanen-Dolenc H Groop PH. Physical activity in the prevention of development and progression of kidney disease in type 1 diabetes. Curr Diabetes Rep. (2019) 19:41. doi: 10.1007/s11892-019-1157-y, PMID: 31152254 PMC6544601

[12] Nosarev AV Smagliy LV Anfinogenova Y Popov SV Kapilevich LV. Exercise and NO production: relevance and implications in the cardiopulmonary system. Front Cell Dev Biol. (2014) 2:73. doi: 10.3389/fcell.2014.00073, PMID: 25610830 PMC4285794

[13] Bird SR Hawley JA. Update on the effects of physical activity on insulin sensitivity in humans. BMJ Open Sport Exerc Med. (2016) 2:e000143. doi: 10.1136/bmjsem-2016-000143, PMID: 28879026 PMC5569266

[14] Gupta J Mitra N Kanetsky PA Devaney J Wing MR Reilly M. Association between albuminuria, kidney function, and inflammatory biomarker profile in CKD in CRIC. Clin J Am Soc Nephrol. (2012) 7:1938–46. doi: 10.2215/CJN.03500412, PMID: 23024164 PMC3513744

[15] Straznicky NE Grima MT Lambert EA Eikelis N Dawood T Lambert GW. Exercise augments weight loss induced improvement in renal function in obese metabolic syndrome individuals. J Hypertens. (2011) 29:553–64. doi: 10.1097/HJH.0b013e3283418875, PMID: 21119532

[16] Zhou Y Hellberg M Hellmark T Hoglund P Clyne N. Muscle mass and plasma myostatin after exercise training: a substudy of Renal Exercise (RENEXC)-a randomized controlled trial. Nephrol Dial Transplant. (2021) 36:95–103. doi: 10.1093/ndt/gfz210, PMID: 31848626 PMC7771980

[17] Hoshino J Ohigashi T Tsunoda R Ito Y Kai H Saito C. Physical activity and renal outcome in diabetic and non-diabetic patients with chronic kidney disease stage G3b to G5. Sci Rep. (2024) 14:26378. doi: 10.1038/s41598-024-77497-1, PMID: 39487292 PMC11530613

[18] Ammar A Trabelsi K Hermassi S Kolahi AA Mansournia MA Jahrami H. Global disease burden attributed to low physical activity in 204 countries and territories from 1990 to 2019: Insights from the Global Burden of Disease 2019 Study. Biol Sport. (2023) 40:835–55. doi: 10.5114/biolsport.2023.121322, PMID: 37398951 PMC10286621

[19] Limonte CP Pilla SJ Boer IH. Diabetes and chronic kidney disease. JAMA. (2025) 333:343–44. doi: 10.1001/jama.2024.24671, PMID: 39724102

[20] Ferrari AJ Santomauro DF Aali A Abate YH Abbafati C Abbastabar H. Global incidence, prevalence, years lived with disability (YLDs), disability-adjusted life-years (DALYs), and healthy life expectancy (HALE) for 371 diseases and injuries in 204 countries and territories and 811 subnational locations, 1990-2021: a systematic analysis for the Global Burden of Disease Study 2021. Lancet. (2024) 403:2133–61. doi: 10.1016/S0140-6736(24)00757-8, PMID: 38642570 PMC11122111

[21] Huang H Li P Jiang H Hong J Lu Y. Global trends and projections of occupational trichloroethylene (TCE) exposure-associated kidney cancer: Insights of the Global Burden of Disease (GBD) Study 2021 from 1990 to 2021 and prediction to 2050. Ecotoxicol Environ Saf. (2024) 287:117252. doi: 10.1016/j.ecoenv.2024.117252, PMID: 39504875

[22] Naghavi M Wang H Lozano R Davis A Liang X Zhou M. Global, regional, and national age-sex specific all-cause and cause-specific mortality for 240 causes of death, 1990-2013: a systematic analysis for the Global Burden of Disease Study 2013. Lancet. (2015) 385:117–71. doi: 10.1016/S0140-6736(14)61682-2, PMID: 25530442 PMC4340604

[23] Institute for Health Metrics and Evaluation (IHME). Global Burden of Disease Study 2021 (GBD 2021) data resources (2021). Available online at: https://ghdx.healthdata.org/gbd-2021 (Accessed February 5, 2025).

[24] Ong KL Stafford LK McLaughlin SA Boyko EJ Vollset SE Smith AE. Global, regional, and national burden of diabetes from 1990 to 2021, with projections of prevalence to 2050: a systematic analysis for the Global Burden of Disease Study 2021. Lancet. (2023) 402:203–34. doi: 10.1016/S0140-6736(23)01301-6, PMID: 37356446 PMC10364581

[25] Stevens PE Ahmed SB Carrero JJ Foster B Francis A Hall RK . KDIGO 2024 clinical practice guideline for the evaluation and management of chronic kidney disease. Kidney Int. (2024) 105:S117–314. doi: 10.1016/j.kint.2023.10.018, PMID: 38490803

[26] Murray CJL Aravkin AY Zheng P Abbafati C Abbas KM Abbasi-Kangevari M. Global burden of 87 risk factors in 204 countries and territories, 1990-2019: a systematic analysis for the Global Burden of Disease Study 2019. Lancet. (2020) 396:1223–49. doi: 10.1016/S0140-6736(20)30752-2, PMID: 33069327 PMC7566194

[27] Yin X Zhang T Zhang Y Man J Yang X Lu M. The global, regional, and national disease burden of breast cancer attributa ble to low physical activity from 1990 to 2019: an analysis of the Global Burden of Disease Study 2019. Int J Behav Nutr Phys Act. (2022) 19:42. doi: 10.1186/s12966-022-01283-3, PMID: 35366913 PMC8977046

[28] Brauer M Roth GA Aravkin AY Zheng P Abate KH Abate YH. Global burden and strength of evidence for 88 risk factors in 204 countries and 811 subnational locations, 1990-2021: a systematic analysis for the Global Burden of Disease Study 2021. Lancet. (2024) 403:2162–203. doi: 10.1016/S0140-6736(24)00933-4, PMID: 38762324 PMC11120204

[29] The International Society for Physical Activity and Health (ISPAH). The bangkok declaration on physical activity for global health and sustainable development. Br J Sports Med. (2017) 51:1389–91. doi: 10.1136/bjsports-2017-098063, PMID: 28642224

[30] Xu YY Xie J Yin H Yang FF Ma CM Yang BY. The Global Burden of Disease attributa ble to low physical activity and its trends from 1990 to 2019: An analysis of the Global Burden of Disease study. Front Public Health. (2022) 10:1018866. doi: 10.3389/fpubh.2022.1018866, PMID: 36590002 PMC9798308

[31] Lee YJ Park DH Kim C Lee DH Lee YH Lee BW. Meeting physical activity guidelines: impact on chronic kidney disease prevalence in diabetic individuals. Yonsei Med J. (2025) 66:519–28. doi: 10.3349/ymj.2024.0245, PMID: 40709682 PMC12303670

[32] Seidu S Abdool M Almaqhawi A Wilkinson TJ Kunutsor SK Khunti K. Physical activity and risk of chronic kidney disease: systematic review and meta-analysis of 12 cohort studies involving 1,281,727 participants. Eur J Epidemiol. (2023) 38:267–80. doi: 10.1007/s10654-022-00961-7, PMID: 36626101 PMC10033580

[33] Wilund KR Thompson S Viana JL Wang AY. Physical activity and health in chronic kidney disease. Contrib Nephrol. (2021) 199:43–55. doi: 10.1159/000517696, PMID: 34343989

[34] Wilkinson TJ Miksza J Yates T Lightfoot CJ Baker LA Watson EL. Association of sarcopenia with mortality and end-stage renal disease in those with chronic kidney disease: a UK Biobank study. J Cachexia Sarcopenia Muscle. (2021) 12:586–98. doi: 10.1002/jcsm.12705, PMID: 33949807 PMC8200422

[35] Katzmarzyk PT Friedenreich C Shiroma EJ Lee IM. Physical inactivity and non-communicable disease burden in low-income, middle-income and high-income countries. Br J Sports Med. (2022) 56:101–06. doi: 10.1136/bjsports-2020-103640, PMID: 33782046 PMC8478970

[36] Aregbesola A Voutilainen S Virtanen JK Mursu J Tuomainen TP. Gender difference in type 2 diabetes and the role of body iron stores. Ann Clin Biochem. (2017) 54:113–20. doi: 10.1177/0004563216646397, PMID: 27166309

[37] Liu B Widener MJ Smith LG Farber S Gesink D Minaker LM. Who’s cooking tonight? A time-use study of coupled adults in Toronto, Canada. Time Soc. (2022) 31:480–507. doi: 10.1177/0961463X221100696, PMID: 36339032 PMC9630964

[38] Seifarth JE McGowan CL Milne KJ. Sex and life expectancy. Gend Med. (2012) 9:390–401. doi: 10.1016/j.genm.2012.10.001, PMID: 23164528

[39] Clotet-Freixas S Zaslaver O Kotlyar M Pastrello C Quaile AT McEvoy CM. Sex differences in kidney metabolism may reflect sex-dependent outcomes in human diabetic kidney disease. Sci Transl Med. (2024) 16:eabm2090. doi: 10.1126/scitranslmed.abm2090, PMID: 38446901

[40] Xu Y Lu J Li M Wang T Wang K Cao Q. Diabetes in China part 1: epidemiology and risk factors. Lancet Public Health. (2024) 9:e1089–e97. doi: 10.1016/S2468-2667(24)00250-0, PMID: 39579774

[41] Russo GT De Cosmo S Viazzi F Mirijello A Ceriello A Guida P. Diabetic kidney disease in the elderly: prevalence and clinical correlates. BMC Geriatr. (2018) 18:38. doi: 10.1186/s12877-018-0732-4, PMID: 29394888 PMC5797340

[42] Zhang L Wang Y Xiong L Luo Y Huang Z Yi B. Exercise therapy improves eGFR, and reduces blood pressure and BMI in non-dialysis CKD patients: evidence from a meta-analysis. BMC Nephrol. (2019) 20:398. doi: 10.1186/s12882-019-1586-5, PMID: 31664943 PMC6821004

[43] Safiri S Kolahi AA Cross M Hill C Smith E Carson-Chahhoud K. Prevalence, deaths, and disability-adjusted life years due to musculoskeletal disorders for 195 countries and territories 1990-2017. Arthritis Rheumatol. (2021) 73:702–14. doi: 10.1002/art.41571, PMID: 33150702

[44] Johnson LR. Physical activity differs with sex and age. BMJ. (2019) 366:l5694. doi: 10.1136/bmj.l5694, PMID: 31570410

[45] Bishop NC Burton JO Graham-Brown MPM Stensel DJ Viana JL Watson EL. Exercise and chronic kidney disease: potential mechanisms underlying the physiological benefits. Nat Rev Nephrol. (2023) 19:244–56. doi: 10.1038/s41581-022-00675-9, PMID: 36650232

[46] Guthold R Stevens GA Riley LM Bull FC. Global trends in insufficient physical activity among adolescents: a pooled analysis of 298 population-based surveys with 1.6 million participants. Lancet Child Adolesc Health. (2020) 4:23–35. doi: 10.1016/S2352-4642(19)30323-2, PMID: 31761562 PMC6919336

[47] Li S Lear SA Rangarajan S Hu B Yin L Bangdiwala SI. Association of sitting time with mortality and cardiovascular events in high-income, middle-income, and low-income countries. JAMA Cardiol. (2022) 7:796–807. doi: 10.1001/jamacardio.2022.1581, PMID: 35704349 PMC9201743

[48] Frenk J Chen L Bhutta ZA Cohen J Crisp N Evans T. Health professionals for a new century: transforming education to strengthen health systems in an interdependent world. Lancet. (2010) 376:1923–58. doi: 10.1016/S0140-6736(10)61854-5, PMID: 21112623

[49] Rudnicka E Napierala P Podfigurna A Meczekalski B Smolarczyk R Grymowicz M. The World Health Organization (WHO) approach to healthy ageing. Maturitas. (2020) 139:6–11. doi: 10.1016/j.maturitas.2020.05.018, PMID: 32747042 PMC7250103

[50] Al Ali R Rastam S Fouad FM Mzayek F Maziak W. Modifiable cardiovascular risk factors among adults in Aleppo, Syria. Int J Public Health. (2011) 56:653–62. doi: 10.1007/s00038-011-0278-0, PMID: 21814848

[51] Gupta R Deedwania PC Sharma K Gupta A Guptha S Achari V. Association of educational, occupational and socioeconomic status with cardiovascular risk factors in Asian Indians: a cross-sectional study. PloS One. (2012) 7:e44098. doi: 10.1371/journal.pone.0044098, PMID: 22952886 PMC3430674

[52] Peters DH Garg A Bloom G Walker DG Brieger WR Rahman MH. Poverty and access to health care in developing countries. Ann N Y Acad Sci. (2008) 1136:161–71. doi: 10.1196/annals.1425.011, PMID: 17954679

[53] Ma X Liu R Xi X Zhuo H Gu Y. Global burden of chronic kidney disease due to diabetes mellitus, 1990-2021, and projections to 2050. Front Endocrinol (Lausanne). (2025) 16:1513008. doi: 10.3389/fendo.2025.1513008, PMID: 40060381 PMC11885120

[54] Tobias DK Merino J Ahmad A Aiken C Benham JL Bodhini D. Second international consensus report on gaps and opportunities for the clinical translation of precision diabetes medicine. Nat Med. (2023) 29:2438–57. doi: 10.1038/s41591-023-02502-5, PMID: 37794253 PMC10735053

[55] Tremblay MS Warburton DE Janssen I Paterson DH Latimer AE Rhodes RE. New Canadian physical activity guidelines. Appl Physiol Nutr Metab. (2011) 36:36–46. doi: 10.1139/H11-009, PMID: 21326376

[56] Florindo AA Salvador EP Reis RS Guimaraes VV. Perception of the environment and practice of physical activity by adults in a low socioeconomic area. Rev Saude Publica. (2011) 45:302–10. doi: 10.1590/s0034-89102011000200009, PMID: 21412570

