# Supplementary material for: Global, regional, and national burden of type 2 diabetes-related diabetic kidney disease attributable to low physical activity from 1990 to 2021: a systematic analysis of the Global Burden of Disease Study 2021 with predictions to 2050
Source: Front Endocrinol (Lausanne). 2025 Sep 17;16:1625973. doi: 10.3389/fendo.2025.1625973 (PMC12483903; doi:10.3389/fendo.2025.1625973)
Supplement: Supplementary file 1 [file Supplementaryfile1.docx]

**Supplementary Figure S1** The summary map of DKD attributable to LPA burden in 2021 stratified by 204 countries and territories. (A) number of YLDs cases. (B) number of YLLs cases. (C) age-standardized YLDs rate. (D) age-standardized YLLs rate

**Supplementary Figure S2** Changes of EAPC in the burden of DKD attributable to LPA stratified by countries from 1990 to 2021. (A) changes in YLDs. (B) changes in YLLs.

**Supplementary Figure S2** Changes of EAPC in the burden of DKD attributable to LPA stratified by countries from 1990 to 2021. (A) changes in YLDs. (B) changes in YLLs.

**Supplementary Figure S3** SDI-related health inequality regression (A) and concentration (B) curves for the YLDs of DKD attributable to LPA, 1990 and 2019. SDI-related health inequality regression (C) and concentration (D) curves for the YLLs of DKD attributable to LPA, 1990 and 2019

**Table S1** The number of YLDs and age-standardized YLDs rate of DKD attributable to low physical activity in 1990 and 2021 with EAPC from 1990 to 2021 globally.

**Table S2** The number of YLLs and age-standardized YLLs rate of DKD attributable to low physical activity in 1990 and 2021 with EAPC from 1990 to 2021 globally

**Table S3** The number of deaths and age-standardized deaths rate of DKD attributable to low physical activity stratified by regions and countries in 1990 and 2021 with EAPC from 1990 to 2021 globally.

**Table S4** The number of deaths and age-standardized DALYs rate of DKD attributable to low physical activity stratified by regions and countries in 1990 and 2021 with EAPC from 1990 to 2021 globally.

**Table S5** The number of deaths and age-standardized YLDs rate of DKD attributable to low physical activity stratified by regions and countries in 1990 and 2021 with EAPC from 1990 to 2021 globally.

**Table S6** The number of deaths and age-standardized YLLs rate of DKD attributable to low physical activity stratified by regions and countries in 1990 and 2021 with EAPC from 1990 to 2021 globally.


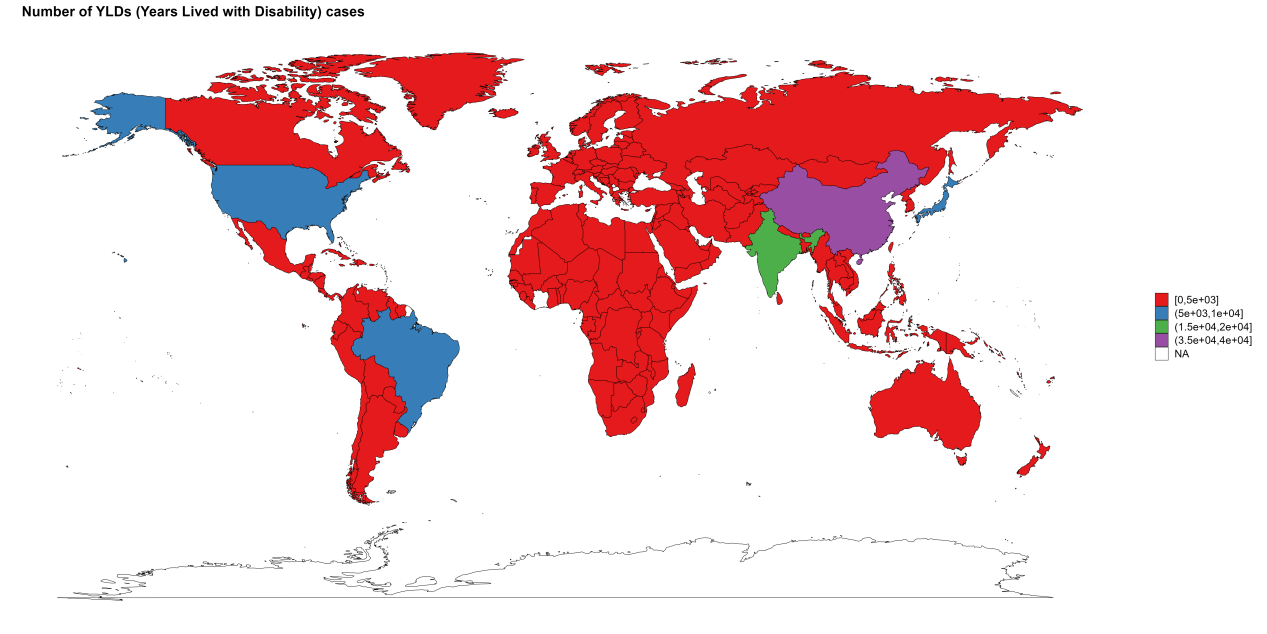


A


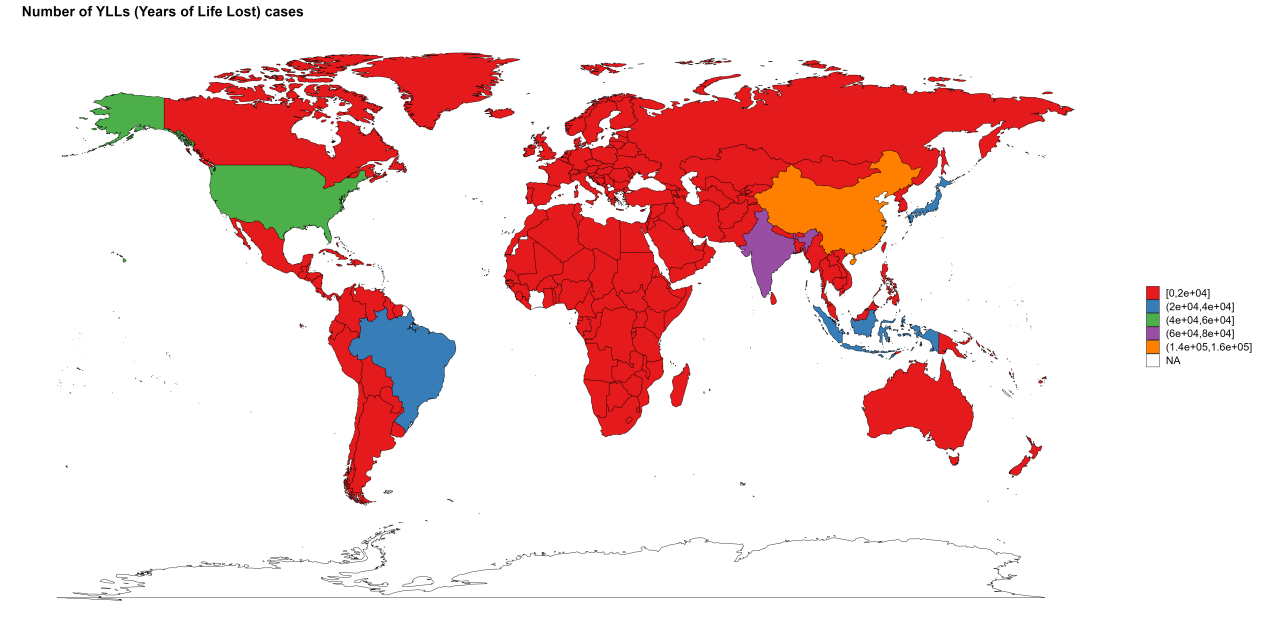


B


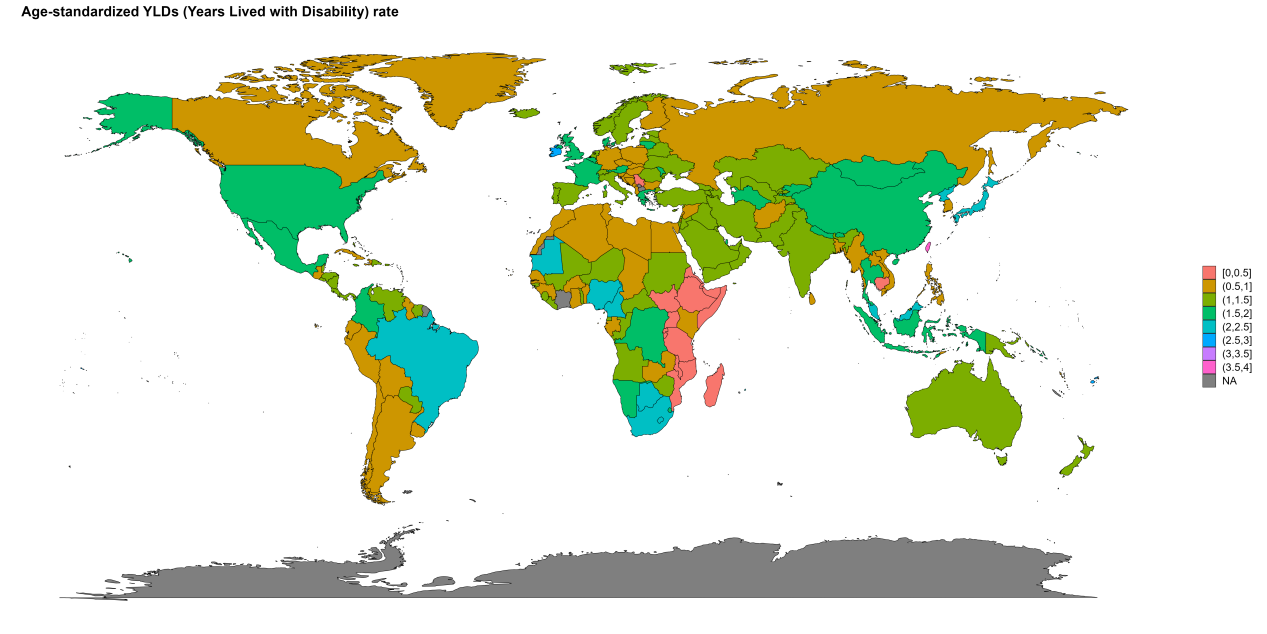


C


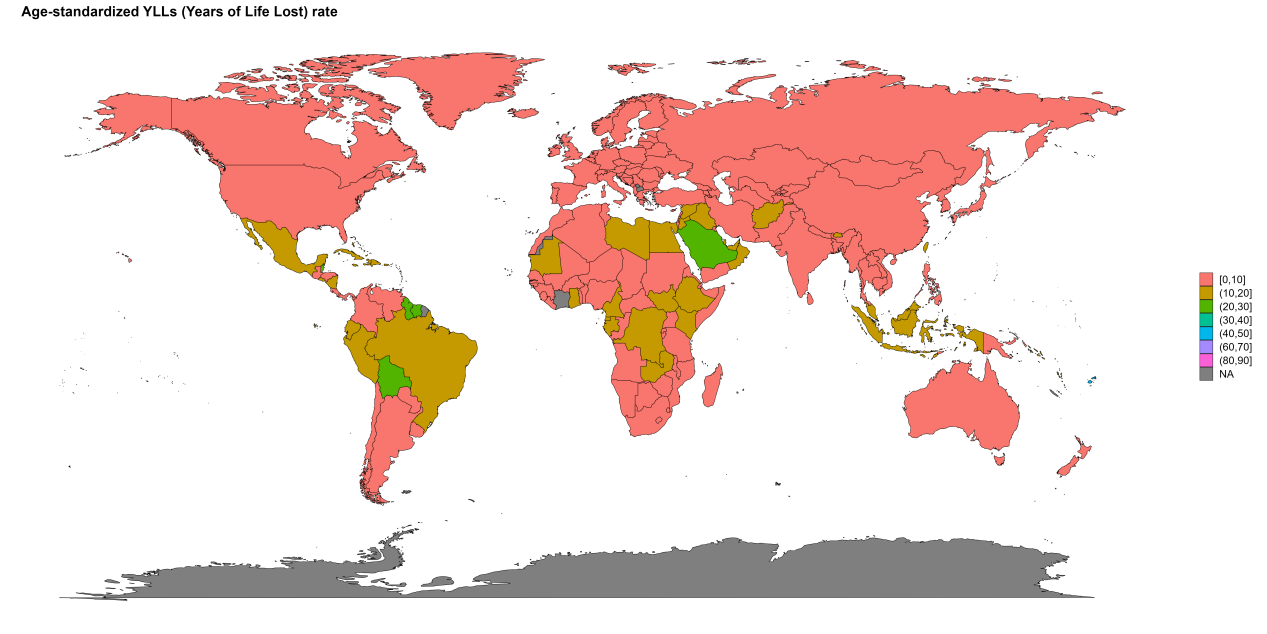


D

**Fig. S1** The summary map of DKD attributable to LPA burden in 2021 stratified by 204 countries and territories. (A) number of YLDs cases. (B) number of YLLs cases. (C) age-standardized YLDs rate. (D) age-standardized YLLs rate


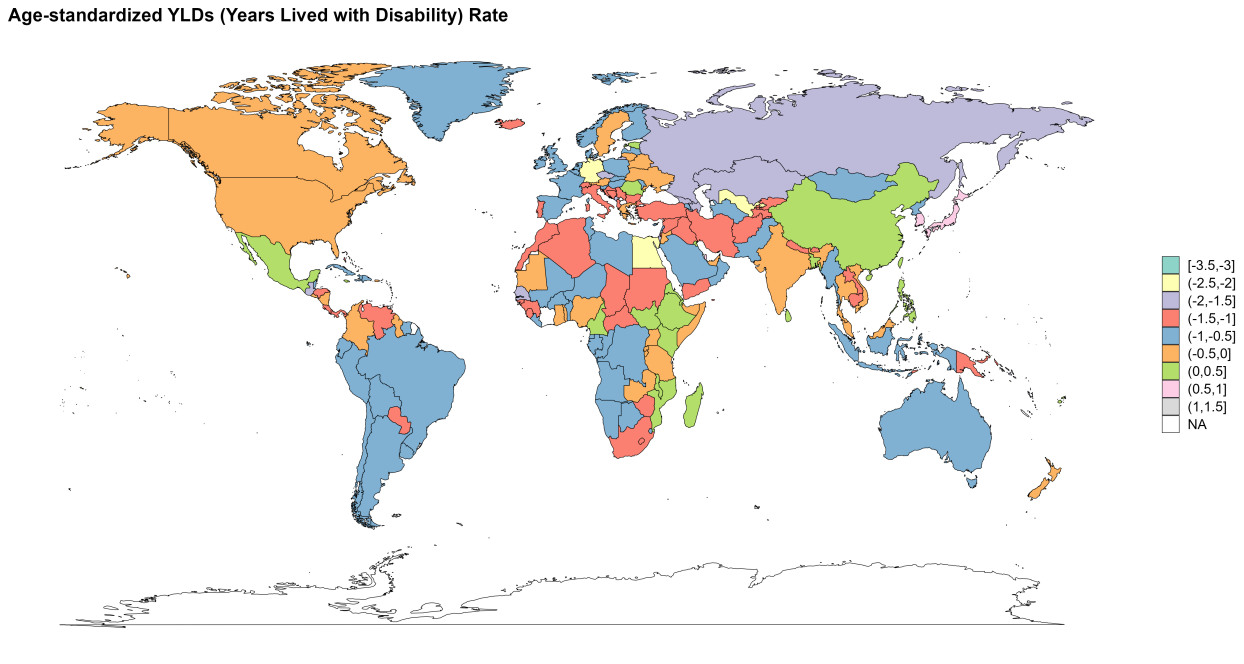


A


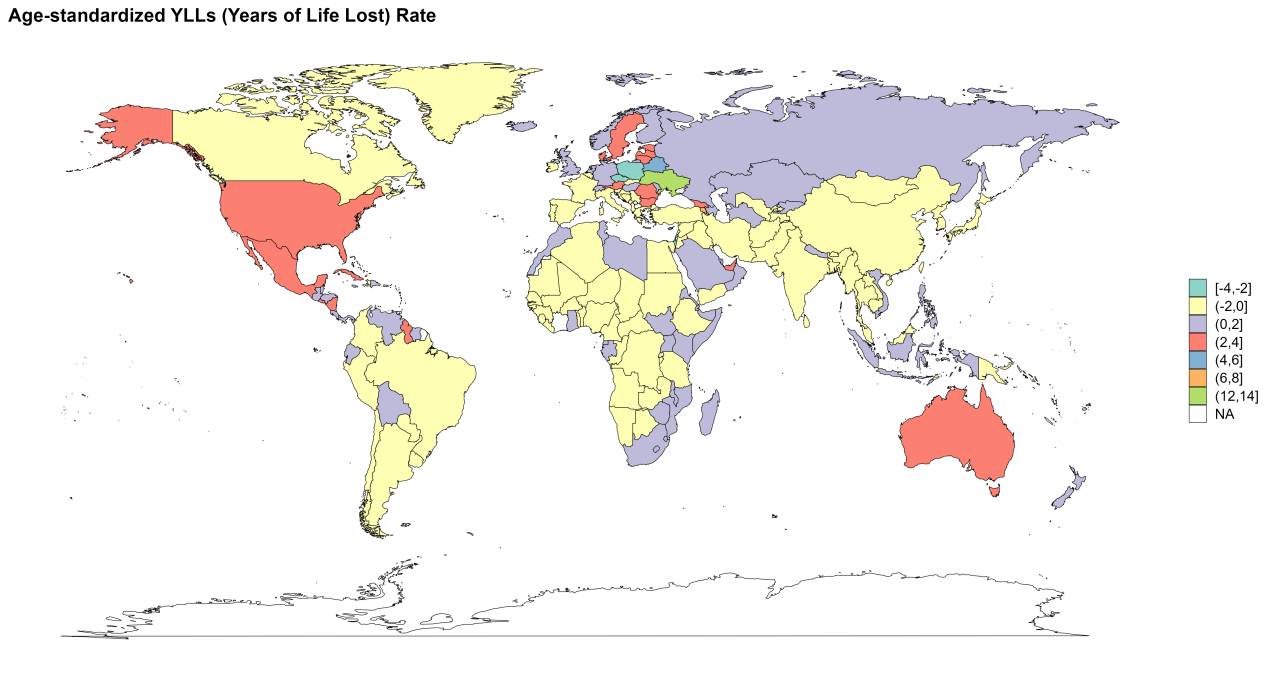


B

**Fig. S2.** Changes of EAPC in the burden of DKD attributable to LPA stratified by countries from 1990 to 2021. (A) changes in YLDs. (B) changes in YLLs.


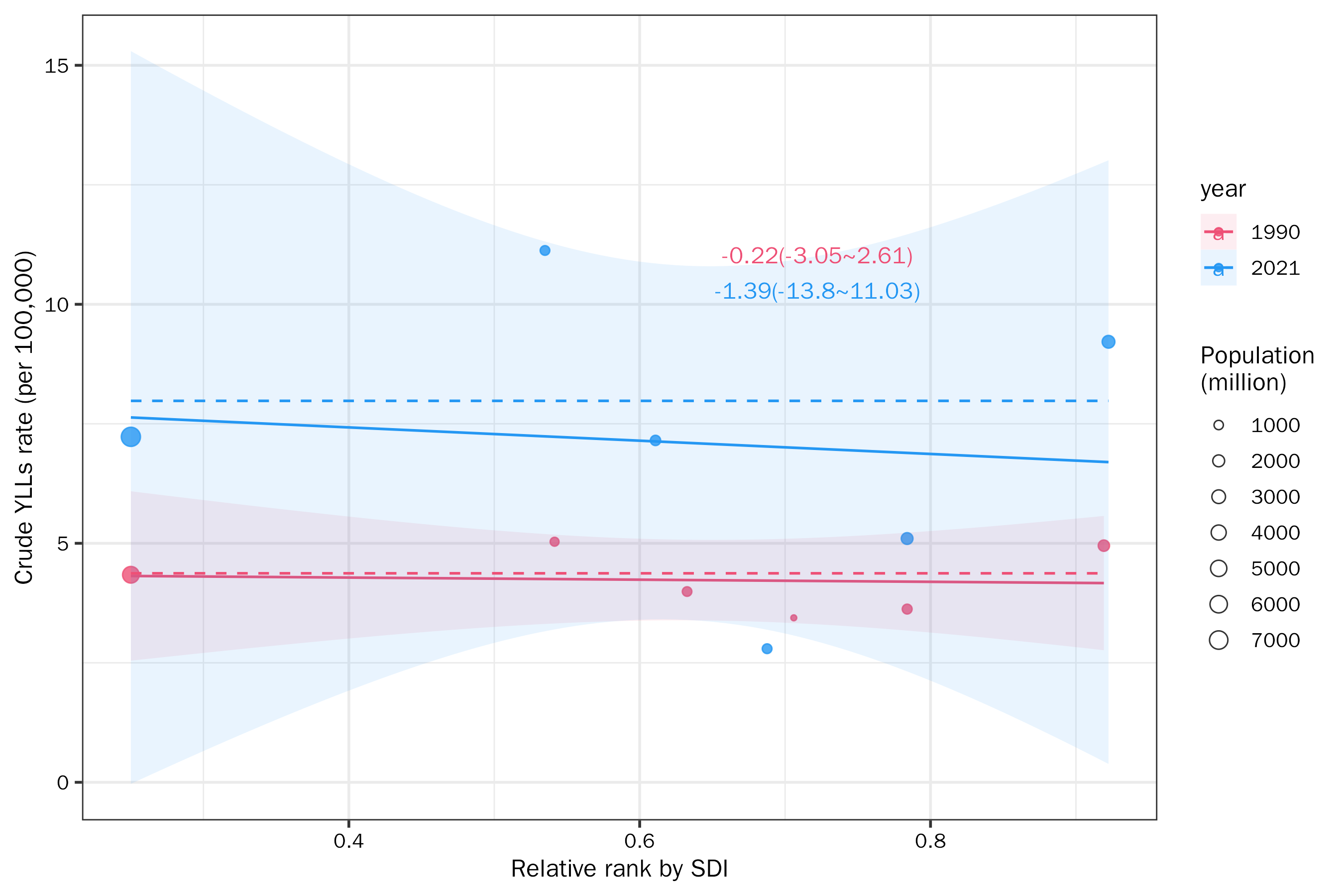


C


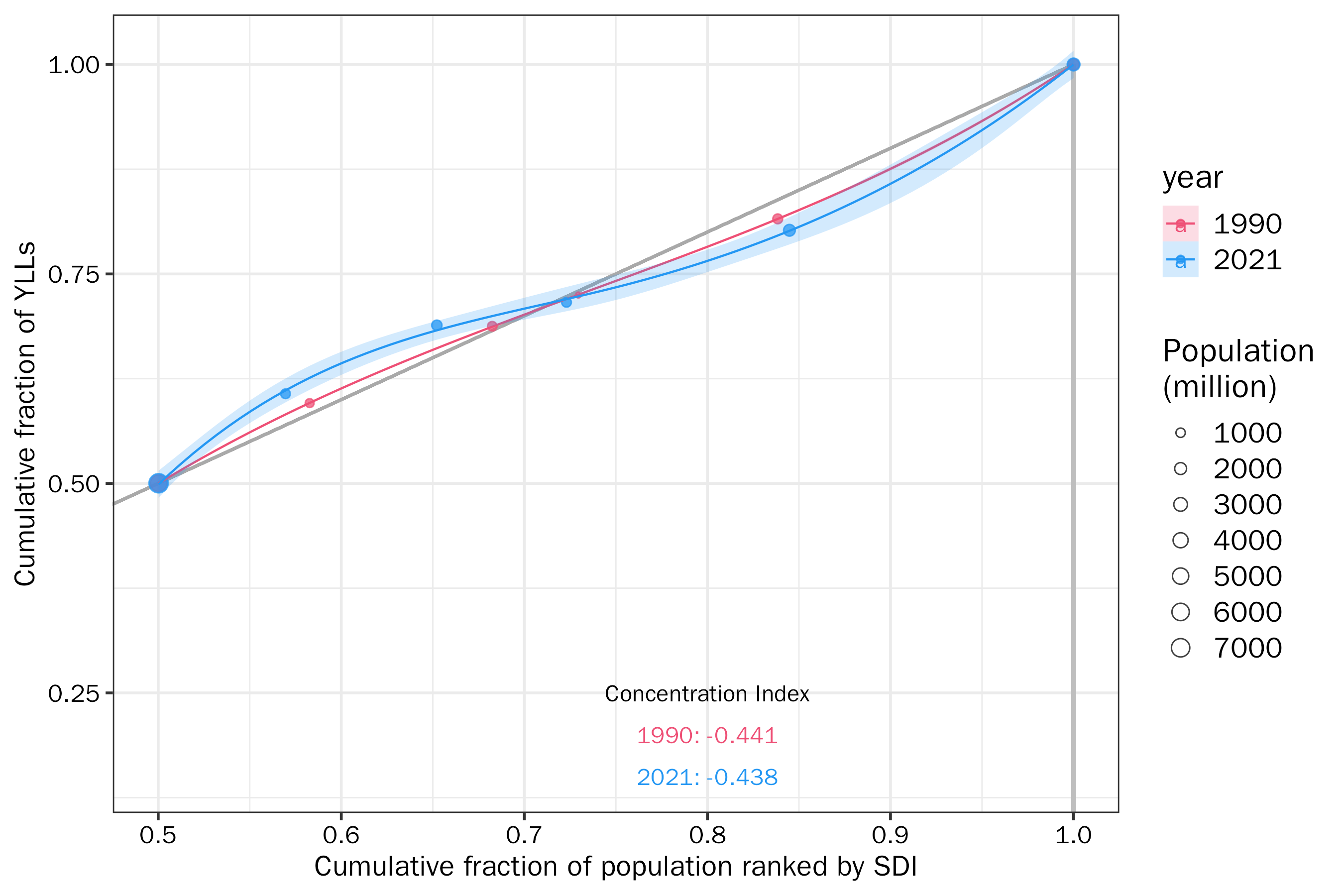


D


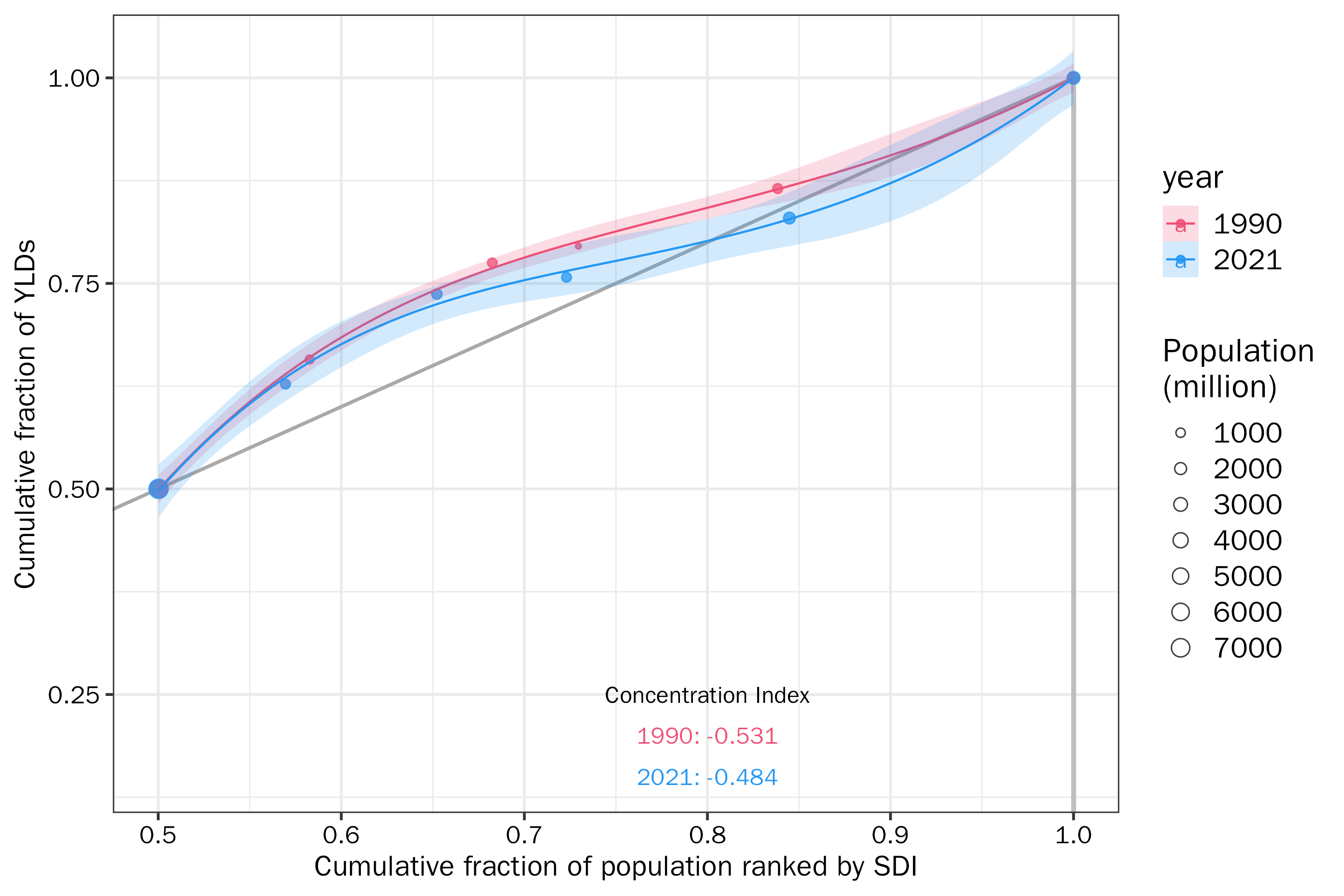


A

B


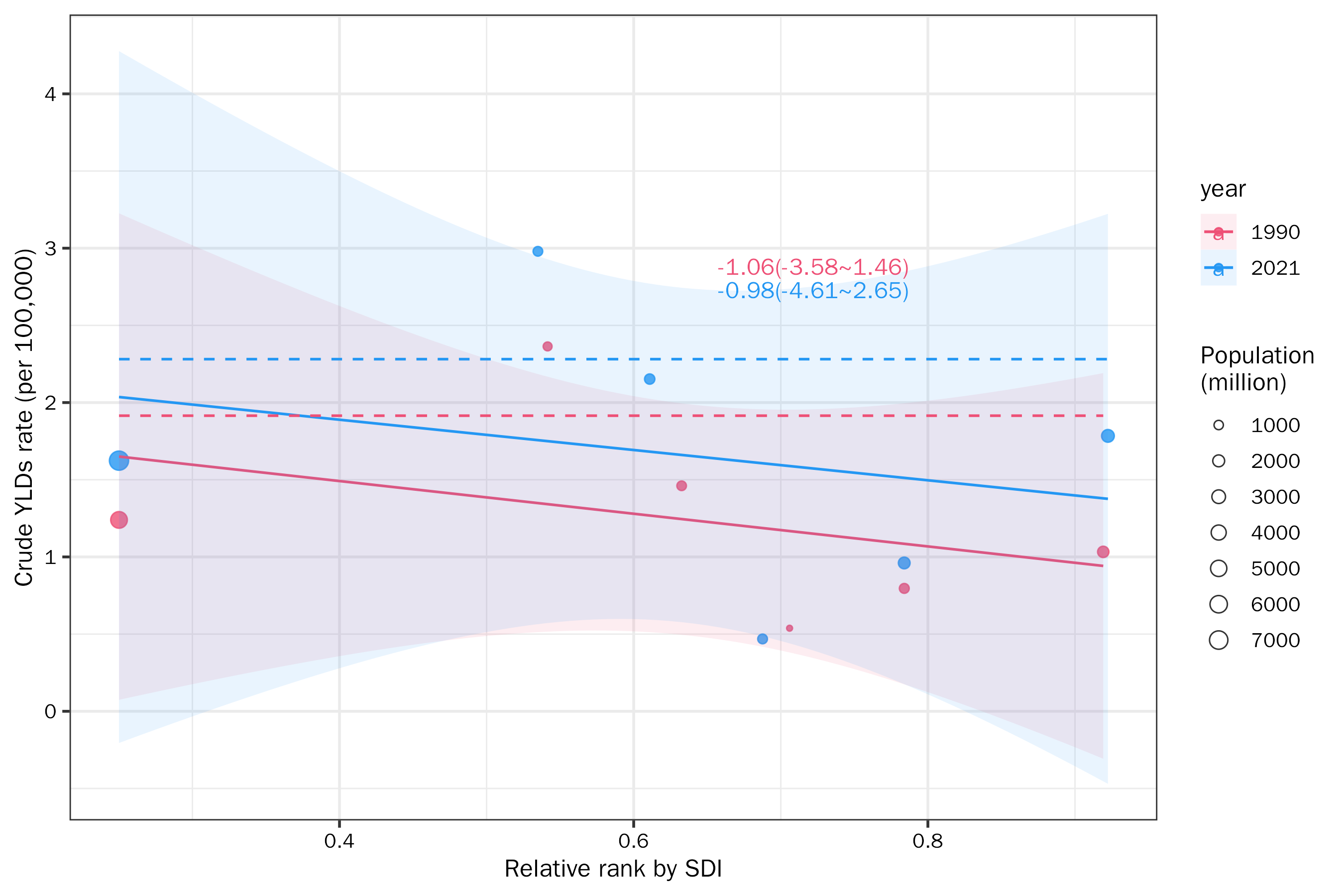


**Figure. S3.** SDI-related health inequality regression (A) and concentration (B) curves for the YLDs of DKD attributable to LPA, 1990 and 2019. SDI-related health inequality regression (C) and concentration (D) curves for the YLLs of DKD attributable to LPA, 1990 and 2019

**Table S1** The number of YLDs and age-standardized YLDs rate of DKD attributable to low physical activity in 1990 and 2021 with EAPC from 1990 to 2021 globally

| Items | 1990 | | 2021 | | 1990-2021 |
| --- | --- | --- | --- | --- | --- |
|  | Number of YLDs(95% UI) | The age-standardized YLDs rate/100000(95% UI) | Number of YLDs(95% UI) | The age-standardized YLDs rate/100000(95% UI) | EAPC(95%CI) |
| **Global** | 66127 (24806-116887) | 1.71 (0.65-3.02) | 128134 (49280-226865) | 1.49 (0.57-2.64) | 0.33(0.06-0.60) |
| **Sex** |  |  |  |  |  |
| Female | 37797 (14294-66610) | 1.79 (0.68-3.16) | 73021 (28290-129326) | 1.59 (0.62-2.82) | -0.25(-0.37--0.14) |
| Male | 28330 (10710-49619) | 1.63 (0.62-2.87) | 55112 (20997-96963) | 1.38 (0.53-2.41) | -0.47(-0.56--0.38) |
| **Age groups** |  |  |  |  |  |
| 25-29 | 553 (156-1278) | 0.12 (0.04-0.29) | 496 (150-1115) | 0.08 (0.03-0.19) | -1.33(-1.41--1.25) |
| 30-34 | 964 (284-2137) | 0.25 (0.07-0.55) | 1236 (389-2680) | 0.2 (0.06-0.44) | -0.50(-0.64--0.36) |
| 35-39 | 1648 (521-3457) | 0.47 (0.15-0.98) | 2502 (795-5261) | 0.45 (0.14-0.94) | -0.01(-0.13-0.12) |
| 40-44 | 2416 (737-5172) | 0.84 (0.26-1.81) | 4292 (1428-8665) | 0.86 (0.29-1.73) | 0.20(0.10-0.30) |
| 45-49 | 3397 (1090-6533) | 1.46 (0.47-2.81) | 7031 (2547-13998) | 1.48 (0.54-2.96) | 0.19(0.08-0.29) |
| 50-54 | 4913 (1624-9577) | 2.31 (0.76-4.51) | 10788 (3714-20801) | 2.42 (0.83-4.68) | 0.29(0.19-0.38) |
| 55-59 | 7044 (2418-13558) | 3.8 (1.31-7.32) | 14943 (5410-28934) | 3.78 (1.37-7.31) | 0.14(0.01-0.26) |
| 60-64 | 8947 (3188-16537) | 5.57 (1.98-10.3) | 15912 (5785-28774) | 4.97 (1.81-8.99) | -0.18(-0.34--0.03) |
| 65-69 | 9289 (3554-16856) | 7.51 (2.87-13.64) | 17573 (6404-32434) | 6.37 (2.32-11.76) | -0.37(-0.52--0.22) |
| 70-74 | 8547 (3270-16140) | 10.1 (3.86-19.06) | 17421 (6223-32959) | 8.46 (3.02-16.01) | -0.46(-0.59--0.33) |
| 75-79 | 8209 (2881-15280) | 13.34 (4.68-24.82) | 14478 (5358-27131) | 10.98 (4.06-20.57) | -0.57(-0.68--0.47) |
| 80-84 | 6129 (2208-11089) | 17.32 (6.24-31.35) | 11651 (4163-20505) | 13.3 (4.75-23.41) | -0.83(-0.92--0.73) |
| 85-89 | 3014 (1169-5381) | 19.94 (7.73-35.61) | 6605 (2496-11693) | 14.45 (5.46-25.58) | -1.07(-1.13--1.02) |

**Table S1 (Continued).**

| Items | 1990 | | | | 2021 | | | | | 1990-2021 |
| --- | --- | --- | --- | --- | --- | --- | --- | --- | --- | --- |
|  | Number of YLDs(95% UI) | | The age-standardized YLDs rate/100000(95% UI) | | Number of YLDs(95% UI) | | | The age-standardized YLDs rate/100000(95% UI) | | EAPC(95%CI) |
| 90-94 | | 1008 (371-1854) | | 23.53 (8.66-43.26) | | 2970 (1131-5502) | 16.6 (6.32-30.76) | | -1.20(-1.24--1.15) | |
| 95+ | | 51 (20-93) | | 4.99 (1.97-9.17) | | 237 (93-429) | 4.36 (1.7-7.88) | | -0.37(-0.49--0.25) | |
| **SDI regions** | |  | |  | |  |  | |  | |
| Low SDI | | 2698 (992-4883) | | 1.27 (0.46-2.31) | | 5237 (1937-9555) | 1.02 (0.39-1.84) | | -0.75(-0.8--0.69) | |
| Low-middle SDI | | 9246 (3494-16499) | | 1.59 (0.61-2.83) | | 18458 (6783-33228) | 1.28 (0.47-2.3) | | -0.6(-0.73--0.46) | |
| Middle SDI | | 17794 (6828-31433) | | 1.77 (0.68-3.11) | | 43686 (16480-78136) | 1.62 (0.61-2.9) | | -0.11(-0.29-0.06) | |
| High-middle SDI | | 15536 (5922-27998) | | 1.61 (0.61-2.9) | | 28066 (10812-49598) | 1.42 (0.55-2.51) | | -0.22(-0.4--0.05) | |
| High SDI | | 20790 (7867-36393) | | 1.89 (0.71-3.28) | | 32601 (12714-55529) | 1.64 (0.64-2.79) | | -0.38(-0.49--0.26) | |

**Table S2** The number of YLLs and age-standardized YLLs rate of DKD attributable to low physical activity in 1990 and 2021 with EAPC from 1990 to 2021 globally

| Items | 1990 | | 2021 | | 1990-2021 |
| --- | --- | --- | --- | --- | --- |
|  | Number of YLLs(95% UI) | The age-standardized YLLs rate/100000(95% UI) | Number of YLLs(95% UI) | The age-standardized YLLs rate/100000(95% UI) | EAPC(95%CI) |
| **Global** | 231571 (91970-388024) | 6.16 (2.49-10.24) | 570350 (226001-965286) | 6.7 (2.64-11.36) | 0.96(0.67-1.25) |
| **Sex** |  |  |  |  |  |
| Female | 125044 (52043-213027) | 6.03 (2.52-10.27) | 305855 (123296-512886) | 6.6 (2.65-11.09) | 0.24(0.16-0.31) |
| Male | 106527 (42448-185405) | 6.42 (2.5-10.83) | 264496 (103132-450041) | 6.88 (2.71-11.68) | 0.26(0.22-0.29) |
| **Age groups** |  |  |  |  |  |
| 25-29 | 461 (101-1263) | 0.1 (0.02-0.29) | 518 (119-1331) | 0.09 (0.02-0.23) | -0.61(-0.73--0.48) |
| 30-34 | 1436 (353-3424) | 0.37 (0.09-0.89) | 2122 (559-5000) | 0.35 (0.09-0.83) | -0.21(-0.33--0.09) |
| 35-39 | 3680 (890-8669) | 1.04 (0.25-2.46) | 5644 (1585-12844) | 1.01 (0.28-2.29) | -0.07(-0.21-0.07) |
| 40-44 | 6022 (1753-13333) | 2.1 (0.61-4.65) | 11652 (3650-25743) | 2.33 (0.73-5.15) | 0.30(0.19-0.40) |
| 45-49 | 9414 (2765-19574) | 4.05 (1.19-8.43) | 21239 (6725-42848) | 4.49 (1.42-9.05) | 0.39(0.32-0.46) |
| 50-54 | 16115 (5292-31923) | 7.58 (2.49-15.02) | 37537 (13289-72630) | 8.44 (2.99-16.32) | 0.36(0.32-0.40) |
| 55-59 | 24736 (8706-47654) | 13.36 (4.7-25.73) | 57604 (21405-110074) | 14.56 (5.41-27.82) | 0.27(0.18-0.35) |
| 60-64 | 32624 (11904-60250) | 20.31 (7.41-37.51) | 67847 (27491-128381) | 21.2 (8.59-40.11) | 0.04(-0.07-0.16) |
| 65-69 | 35021 (13158-64421) | 28.33 (10.64-52.12) | 79463 (30064-142469) | 28.81 (10.9-51.65) | 0.02(-0.12-0.16) |
| 70-74 | 34231 (12623-61482) | 40.43 (14.91-72.62) | 82549 (30536-143760) | 40.1 (14.84-69.84) | -0.01(-0.09-0.07) |
| 75-79 | 30053 (12347-51831) | 48.82 (20.06-84.2) | 68803 (27831-121614) | 52.17 (21.1-92.21) | 0.03(-0.04-0.10) |
| 80-84 | 20408 (7908-35973) | 57.69 (22.35-101.69) | 56165 (22720-93993) | 64.13 (25.94-107.32) | 0.32(0.27-0.38) |
| 85-89 | 11386 (4249-20574) | 75.35 (28.12-136.15) | 41752 (16273-74388) | 91.32 (35.59-162.7) | 0.72(0.61-0.84) |

**Table S2 (Continued).**

| Items | 1990 | | | | 2021 | | | | 1990-2021 |
| --- | --- | --- | --- | --- | --- | --- | --- | --- | --- |
|  | Number of YLLs(95% UI) | | | The age-standardized YLLs rate/100000(95% UI) | Number of YLLs(95% UI) | | The age-standardized YLLs rate/100000(95% UI) | | EAPC(95%CI) |
| 90-94 | | 4532 (1654-8300) | 105.76 (38.59-193.69) | | 25400 (10032-45583) | 141.99 (56.08-254.8) | | 1.19(1.06-1.32) | |
| 95+ | | 1452 (495-2960) | 142.62 (48.59-290.69) | | 12056 (4335-22694) | 221.2 (79.54-416.38) | | 1.67(1.55-1.79) | |
| **SDI regions** | |  |  | |  |  | |  | |
| Low SDI | | 17250 (6857-31120) | 8.22 (3.27-14.98) | | 31231 (12210-55269) | 6.67 (2.58-11.71) | | -0.76(-0.86--0.65) | |
| Low-middle SDI | | 42089 (16868-72535) | 7.39 (2.96-12.65) | | 97971 (36831-171548) | 6.99 (2.66-12.17) | | -0.23(-0.31--0.15) | |
| Middle SDI | | 85299 (33080-146007) | 9.07 (3.61-15.24) | | 225652 (85404-385344) | 8.73 (3.32-14.83) | | -0.15(-0.21--0.09) | |
| High-middle SDI | | 42444 (17039-72897) | 4.58 (1.84-7.83) | | 93267 (37837-159180) | 4.74 (1.93-8.11) | | 0.03(-0.11-0.17) | |
| High SDI | | 44265 (17622-75436) | 3.99 (1.59-6.76) | | 121724 (50395-201055) | 5.53 (2.32-9.12) | | 1.22(1.11-1.32) | |

**Table S3** The number of deaths and age-standardized deaths rate of DKD attributable to low physical activity stratified by regions and countries in 1990 and 2021 with EAPC from 1990 to 2021 globally.

| Items | 1990 | | 2021 | | 1990-2021 |
| --- | --- | --- | --- | --- | --- |
|  | Number of deaths (95% UI) | The age-standardized deaths rate/100000(95% UI) | Number of deaths(95% UI) | The age-standardized deaths rate/100000(95% UI) | EAPC(95%CI) |
| GBD Regions |  |  |  |  |  |
| Advanced Health System | 3326 (1326-5697) | 0.21 (0.08-0.36) | 9394 (3703-15620) | 0.28 (0.11-0.46) | 2.1 (1.42-2.8) |
| Africa | 1107 (432-1967) | 0.49 (0.19-0.85) | 2224 (923-3817) | 0.44 (0.17-0.75) | -0.49 (-1.87-0.91) |
| African Region | 794 (316-1428) | 0.45 (0.18-0.78) | 1602 (655-2746) | 0.42 (0.17-0.72) | -0.49 (-1.97-1) |
| America | 2050 (830-3446) | 0.35 (0.14-0.58) | 7292 (2946-11974) | 0.53 (0.21-0.86) | 2.28 (2.04-2.52) |
| Andean Latin America | 163 (63-283) | 0.91 (0.35-1.58) | 489 (196-823) | 0.86 (0.34-1.45) | 0.77 (0.09-1.47) |
| Asia | 6641 (2703-11194) | 0.44 (0.18-0.73) | 18459 (7271-32091) | 0.41 (0.16-0.71) | 0.89 (0.26-1.52) |
| Australasia | 15 (6-26) | 0.07 (0.03-0.12) | 56 (20-102) | 0.09 (0.03-0.17) | 2.62 (1.94-3.32) |
| Basic Health System | 5540 (2274-9327) | 0.49 (0.2-0.81) | 15850 (6283-26739) | 0.48 (0.19-0.81) | 1.11 (0.53-1.7) |
| Caribbean | 141 (60-236) | 0.61 (0.25-0.99) | 387 (162-645) | 0.71 (0.3-1.18) | 2.02 (1.71-2.34) |
| Central Africa | 156 (63-292) | 0.74 (0.29-1.33) | 290 (112-537) | 0.61 (0.23-1.11) | -1 (-2.62-0.65) |
| Central Asia | 21 (7-39) | 0.05 (0.02-0.09) | 55 (22-97) | 0.08 (0.03-0.13) | 1.22 (0.6-1.85) |
| Central Europe | 131 (52-236) | 0.1 (0.04-0.17) | 157 (59-266) | 0.07 (0.02-0.11) | -0.17 (-0.79-0.45) |
| Central Latin America | 284 (112-490) | 0.42 (0.16-0.71) | 1197 (496-2032) | 0.49 (0.2-0.84) | 2.48 (1.69-3.28) |
| Central Sub-Saharan Africa | 123 (51-232) | 0.78 (0.31-1.41) | 229 (85-431) | 0.6 (0.22-1.14) | -1.13 (-2.84-0.6) |
| Commonwealth High Income | 171 (63-297) | 0.11 (0.04-0.2) | 347 (124-610) | 0.11 (0.04-0.19) | 1.46 (0.69-2.25) |
| Commonwealth Low Income | 192 (71-364) | 0.27 (0.1-0.48) | 490 (187-872) | 0.26 (0.1-0.47) | 0.55 (-0.83-1.95) |

| Commonwealth Middle Income | 1599 (643-2788) | 0.32 (0.13-0.55) | 4125 (1617-7274) | 0.31 (0.12-0.53) | 0.44 (-0.48-1.36) |
| --- | --- | --- | --- | --- | --- |
| East Asia | 3227 (1302-5659) | 0.55 (0.23-0.97) | 8979 (3524-15385) | 0.47 (0.19-0.81) | 0.97 (0.4-1.54) |
| East Asia & Pacific - WB | 4900 (2003-8398) | 0.5 (0.2-0.83) | 13932 (5606-24031) | 0.45 (0.18-0.78) | 1.09 (0.62-1.57) |
| Eastern Africa | 282 (105-522) | 0.5 (0.19-0.91) | 592 (220-1049) | 0.49 (0.18-0.88) | -0.32 (-1.93-1.32) |
| Eastern Europe | 81 (32-143) | 0.03 (0.01-0.05) | 213 (79-363) | 0.06 (0.02-0.1) | 2.83 (2.23-3.43) |
| Eastern Mediterranean Region | 859 (341-1460) | 0.57 (0.23-0.98) | 1886 (772-3219) | 0.48 (0.19-0.81) | -0.34 (-1.43-0.77) |
| Eastern Sub-Saharan Africa | 293 (107-543) | 0.51 (0.18-0.92) | 603 (215-1082) | 0.5 (0.17-0.9) | -0.39 (-2.09-1.34) |
| Europe | 1405 (555-2482) | 0.14 (0.06-0.25) | 2742 (1057-4707) | 0.14 (0.06-0.24) | 1.39 (0.69-2.1) |
| Europe & Central Asia - WB | 1418 (560-2505) | 0.14 (0.05-0.25) | 2776 (1070-4754) | 0.14 (0.06-0.24) | 1.36 (0.72-2) |
| European Region | 1442 (570-2545) | 0.14 (0.06-0.25) | 2816 (1086-4835) | 0.14 (0.06-0.24) | 1.34 (0.71-1.98) |
| High-income Asia Pacific | 792 (313-1353) | 0.46 (0.18-0.78) | 2395 (932-4366) | 0.35 (0.14-0.62) | 1.35 (0.3-2.41) |
| High-income North America | 830 (331-1436) | 0.23 (0.09-0.39) | 3422 (1420-5528) | 0.48 (0.2-0.78) | 3.16 (2.51-3.81) |
| Latin America & Caribbean - WB | 1257 (496-2156) | 0.56 (0.22-0.93) | 3959 (1590-6569) | 0.57 (0.23-0.95) | 1.47 (0.92-2.03) |
| Limited Health System | 2129 (841-3743) | 0.34 (0.14-0.58) | 5159 (2016-9195) | 0.31 (0.12-0.54) | 0.25 (-0.78-1.28) |
| Middle East & North Africa - WB | 620 (241-1059) | 0.63 (0.24-1.08) | 1473 (607-2510) | 0.5 (0.2-0.84) | -0.09 (-1.11-0.93) |
| Minimal Health System | 247 (103-447) | 0.52 (0.21-0.95) | 407 (161-760) | 0.43 (0.17-0.8) | -1.08 (-2.66-0.52) |
| North Africa and Middle East | 883 (350-1528) | 0.65 (0.25-1.12) | 1951 (794-3333) | 0.5 (0.2-0.84) | -0.14 (-1.09-0.83) |
| North America | 831 (331-1436) | 0.23 (0.09-0.39) | 3422 (1420-5529) | 0.48 (0.2-0.78) | 3.16 (2.51-3.81) |
| Northern Africa | 327 (124-607) | 0.68 (0.25-1.25) | 699 (290-1217) | 0.54 (0.22-0.93) | -0.1 (-1.06-0.87) |
| Oceania | 14 (5-25) | 0.63 (0.25-1.08) | 41 (16-70) | 0.7 (0.29-1.2) | 0.59 (-0.73-1.93) |
| Region of the Americas | 2050 (830-3446) | 0.35 (0.14-0.58) | 7292 (2946-11974) | 0.53 (0.21-0.86) | 2.28 (2.04-2.52) |
| South-East Asia Region | 1823 (703-3134) | 0.33 (0.13-0.56) | 4987 (1919-8919) | 0.32 (0.12-0.56) | 0.78 (-0.06-1.62) |
| South Asia | 1362 (538-2440) | 0.29 (0.12-0.51) | 3609 (1401-6349) | 0.28 (0.11-0.49) | 0.62 (-0.27-1.52) |
| South Asia - WB | 1429 (566-2536) | 0.3 (0.12-0.52) | 3712 (1436-6566) | 0.28 (0.11-0.49) | 0.57 (-0.32-1.46) |
| Southeast Asia | 879 (345-1523) | 0.43 (0.17-0.74) | 2547 (977-4484) | 0.45 (0.17-0.78) | 1.07 (0.29-1.85) |
| Southern Africa | 108 (40-197) | 0.31 (0.11-0.54) | 243 (99-425) | 0.32 (0.13-0.56) | 0.42 (-0.84-1.7) |
| Southern Latin America | 197 (78-360) | 0.46 (0.18-0.84) | 280 (109-511) | 0.31 (0.12-0.55) | -0.23 (-0.59-0.13) |
| Southern Sub-Saharan Africa | 62 (23-109) | 0.27 (0.1-0.47) | 131 (52-240) | 0.27 (0.11-0.48) | 0.83 (-0.11-1.78) |
| Sub-Saharan Africa - WB | 786 (312-1411) | 0.45 (0.17-0.76) | 1535 (624-2630) | 0.4 (0.16-0.7) | -0.6 (-2.1-0.91) |
| Tropical Latin America | 478 (190-797) | 0.64 (0.26-1.08) | 1620 (652-2751) | 0.65 (0.26-1.11) | 1.32 (0.76-1.89) |
| Western Africa | 233 (88-428) | 0.37 (0.14-0.68) | 399 (156-713) | 0.3 (0.11-0.53) | -1.3 (-2.78-0.21) |
| Western Europe | 1001 (388-1797) | 0.17 (0.07-0.3) | 1988 (755-3503) | 0.15 (0.06-0.27) | 1.21 (0.16-2.27) |
| Western Pacific Region | 4082 (1667-7030) | 0.49 (0.19-0.82) | 11764 (4738-20086) | 0.44 (0.17-0.75) | 1.14 (0.68-1.59) |
| Western Sub-Saharan Africa | 274 (102-488) | 0.4 (0.15-0.7) | 486 (188-862) | 0.32 (0.12-0.57) | -1.18 (-2.66-0.33) |
| World Bank High Income | 3032 (1213-5225) | 0.24 (0.1-0.4) | 8805 (3475-14707) | 0.31 (0.12-0.51) | 2.05 (1.26-2.84) |
| World Bank Low Income | 629 (256-1111) | 0.55 (0.22-0.97) | 1108 (443-1950) | 0.44 (0.17-0.78) | -0.91 (-2.36-0.56) |
| World Bank Lower Middle Income | 2809 (1118-4838) | 0.33 (0.13-0.56) | 7143 (2758-12455) | 0.33 (0.13-0.57) | 0.59 (-0.31-1.49) |
| World Bank Upper Middle Income | 4772 (1938-8042) | 0.41 (0.16-0.68) | 13754 (5503-23372) | 0.43 (0.17-0.73) | 1.37 (0.92-1.82) |
| **Countries** |  |  |  |  |  |
| Afghanistan | 46 (17-88) | 0.72 (0.26-1.37) | 53 (16-106) | 0.59 (0.2-1.21) | -1.88 (-3.19--0.54) |
| Albania | 2 (1-4) | 0.12 (0.04-0.22) | 3 (1-6) | 0.07 (0.03-0.14) | 1.01 (0.46-1.57) |
| Algeria | 44 (17-86) | 0.52 (0.19-0.97) | 143 (61-256) | 0.51 (0.21-0.9) | 1.2 (0.13-2.28) |
| American Samoa | 0 (0-0) | 1.55 (0.63-2.8) | 2 (1-3) | 4.36 (1.78-7.46) | 4.96 (3.95-5.97) |
| Andorra | 0 (0-0) | 0.31 (0.11-0.59) | 0 (0-1) | 0.16 (0.06-0.31) | -0.13 (-0.79-0.54) |
| Angola | 10 (3-19) | 0.36 (0.13-0.66) | 27 (10-52) | 0.32 (0.11-0.6) | -0.64 (-2.48-1.23) |
| Antigua and Barbuda | 0 (0-1) | 0.73 (0.29-1.24) | 1 (0-2) | 1.02 (0.42-1.71) | 1.94 (1.58-2.31) |
| Argentina | 164 (65-303) | 0.55 (0.22-1) | 215 (81-395) | 0.37 (0.14-0.67) | -0.35 (-0.71-0.02) |
| Armenia | 0 (0-0) | 0.01 (0-0.02) | 4 (1-7) | 0.08 (0.03-0.15) | 8.22 (7.4-9.05) |
| Australia | 11 (4-19) | 0.06 (0.02-0.11) | 45 (16-80) | 0.08 (0.03-0.15) | 3.03 (2.31-3.74) |
| Austria | 22 (8-40) | 0.18 (0.07-0.32) | 100 (36-173) | 0.4 (0.15-0.7) | 4.98 (3.84-6.13) |
| Azerbaijan | 3 (1-5) | 0.06 (0.02-0.12) | 6 (2-12) | 0.07 (0.03-0.13) | 1.02 (0.46-1.59) |
| Bahamas | 1 (0-2) | 0.74 (0.3-1.27) | 3 (1-5) | 0.82 (0.31-1.43) | 1.9 (1.34-2.47) |
| Bahrain | 1 (0-1) | 0.5 (0.21-0.85) | 4 (1-7) | 0.76 (0.28-1.38) | 1.37 (-0.53-3.31) |
| Bangladesh | 101 (36-192) | 0.25 (0.09-0.46) | 242 (87-451) | 0.21 (0.07-0.38) | 0.67 (-0.49-1.85) |
| Barbados | 2 (1-4) | 0.79 (0.33-1.31) | 5 (2-9) | 0.98 (0.38-1.69) | 2.05 (1.55-2.56) |
| Belarus | 1 (0-2) | 0.01 (0-0.01) | 4 (1-7) | 0.02 (0.01-0.04) | 4.42 (3.68-5.18) |
| Belgium | 35 (14-61) | 0.22 (0.09-0.38) | 50 (19-92) | 0.16 (0.06-0.28) | 0.79 (-0.27-1.87) |
| Belize | 1 (0-1) | 0.73 (0.29-1.28) | 3 (1-5) | 1 (0.41-1.66) | 1.68 (0.73-2.63) |
| Benin | 4 (1-7) | 0.23 (0.08-0.46) | 7 (3-15) | 0.19 (0.07-0.36) | -0.95 (-2.57-0.69) |
| Bermuda | 0 (0-1) | 0.77 (0.3-1.3) | 1 (0-2) | 0.6 (0.23-1.01) | 1.16 (0.51-1.81) |
| Bhutan | 1 (0-2) | 0.66 (0.23-1.21) | 3 (1-5) | 0.51 (0.18-0.92) | 0.58 (-0.4-1.58) |
| Bolivia (Plurinational State of) | 31 (11-57) | 1.21 (0.43-2.2) | 92 (33-164) | 1.18 (0.43-2.06) | 1.01 (0.1-1.93) |
| Bosnia and Herzegovina | 4 (2-8) | 0.12 (0.04-0.21) | 5 (2-10) | 0.08 (0.03-0.15) | 0.25 (-0.36-0.86) |
| Botswana | 1 (1-3) | 0.3 (0.11-0.55) | 3 (1-6) | 0.26 (0.1-0.48) | 0.2 (-1.05-1.47) |
| Brazil | 466 (185-780) | 0.65 (0.26-1.09) | 1593 (641-2693) | 0.66 (0.26-1.11) | 1.34 (0.78-1.91) |
| Brunei Darussalam | 1 (0-1) | 0.9 (0.35-1.57) | 2 (1-3) | 0.78 (0.3-1.45) | 1.12 (-0.51-2.78) |
| Bulgaria | 6 (3-12) | 0.07 (0.03-0.12) | 14 (5-26) | 0.09 (0.03-0.17) | 2.71 (1.92-3.51) |
| Burkina Faso | 12 (4-22) | 0.35 (0.13-0.65) | 19 (7-36) | 0.27 (0.1-0.51) | -1.06 (-2.54-0.45) |
| Burundi | 9 (3-16) | 0.47 (0.17-0.9) | 13 (4-25) | 0.4 (0.13-0.77) | -1.45 (-3.19-0.32) |
| Cabo Verde | 0 (0-1) | 0.12 (0.05-0.23) | 1 (0-1) | 0.16 (0.06-0.31) | 0.92 (0.35-1.49) |
| Cambodia | 7 (2-13) | 0.23 (0.08-0.42) | 16 (6-32) | 0.17 (0.06-0.35) | -0.09 (-1.45-1.28) |
| Cameroon | 26 (9-49) | 0.77 (0.26-1.44) | 63 (24-122) | 0.66 (0.24-1.26) | -0.59 (-2.19-1.04) |
| Canada | 45 (15-80) | 0.14 (0.05-0.25) | 99 (34-183) | 0.12 (0.04-0.22) | 0.84 (0.08-1.61) |
| Central African Republic | 5 (2-10) | 0.58 (0.22-1.11) | 7 (3-13) | 0.41 (0.15-0.81) | -1.24 (-2.93-0.48) |
| Chad | 8 (3-16) | 0.33 (0.12-0.64) | 11 (4-21) | 0.23 (0.08-0.44) | -2.01 (-3.5--0.49) |
| Chile | 23 (8-42) | 0.26 (0.09-0.47) | 51 (18-92) | 0.19 (0.07-0.34) | 0.54 (0.13-0.95) |
| China | 2993 (1200-5304) | 0.53 (0.22-0.93) | 8453 (3279-14480) | 0.46 (0.18-0.79) | 1 (0.43-1.57) |
| Colombia | 53 (20-96) | 0.35 (0.13-0.65) | 128 (48-234) | 0.23 (0.09-0.41) | 0.52 (-0.08-1.12) |
| Comoros | 0 (0-1) | 0.3 (0.1-0.58) | 1 (0-2) | 0.31 (0.1-0.6) | 0.8 (-0.47-2.08) |
| Congo | 5 (2-10) | 0.58 (0.2-1.17) | 9 (3-18) | 0.44 (0.15-0.83) | -0.98 (-2.42-0.48) |
| Cook Islands | 0 (0-0) | 0.63 (0.23-1.1) | 0 (0-0) | 0.93 (0.38-1.66) | 2.99 (2.51-3.47) |
| Costa Rica | 5 (2-9) | 0.29 (0.11-0.54) | 19 (7-34) | 0.34 (0.13-0.61) | 2.04 (1.47-2.61) |
| Côte d'Ivoire | 11 (4-21) | 0.36 (0.13-0.67) | 22 (8-41) | 0.24 (0.09-0.45) | -1.03 (-2.73-0.71) |
| Croatia | 5 (2-9) | 0.09 (0.03-0.17) | 9 (3-16) | 0.09 (0.03-0.16) | 1.08 (0.3-1.87) |
| Cuba | 33 (14-54) | 0.33 (0.14-0.56) | 109 (42-188) | 0.53 (0.21-0.91) | 3.43 (2.91-3.95) |
| Cyprus | 2 (1-3) | 0.46 (0.17-0.84) | 3 (1-6) | 0.25 (0.1-0.49) | -0.93 (-1.46--0.4) |
| Czechia | 11 (4-21) | 0.08 (0.03-0.15) | 10 (4-17) | 0.04 (0.02-0.07) | -1.3 (-2.01--0.57) |
| Democratic People's Republic of Korea | 81 (30-144) | 0.69 (0.26-1.22) | 154 (59-279) | 0.52 (0.2-0.95) | 0.39 (-0.08-0.85) |
| Democratic Republic of the Congo | 100 (42-187) | 0.93 (0.38-1.69) | 180 (68-340) | 0.7 (0.25-1.37) | -1.14 (-2.81-0.56) |
| Denmark | 11 (4-20) | 0.12 (0.05-0.23) | 38 (15-66) | 0.26 (0.1-0.45) | 3.18 (2.17-4.2) |
| Djibouti | 0 (0-1) | 0.37 (0.13-0.7) | 2 (1-4) | 0.52 (0.17-1.01) | 1.91 (-0.06-3.91) |
| Dominica | 0 (0-1) | 0.69 (0.27-1.17) | 1 (0-1) | 0.88 (0.35-1.55) | 1.65 (1.47-1.84) |
| Dominican Republic | 21 (8-38) | 0.69 (0.25-1.23) | 68 (24-122) | 0.7 (0.25-1.26) | 2.1 (1.38-2.82) |
| Ecuador | 34 (14-62) | 0.74 (0.31-1.32) | 136 (52-252) | 0.89 (0.34-1.65) | 1.68 (0.76-2.6) |
| Egypt | 210 (79-384) | 1.14 (0.43-2.06) | 372 (146-695) | 0.77 (0.3-1.41) | -1 (-2.27-0.28) |
| El Salvador | 13 (5-28) | 0.47 (0.18-0.99) | 54 (20-101) | 0.83 (0.31-1.56) | 3.03 (2.37-3.69) |
| Equatorial Guinea | 1 (0-2) | 0.63 (0.23-1.16) | 2 (1-5) | 0.68 (0.2-1.34) | 0.04 (-1.8-1.91) |
| Eritrea | 1 (0-3) | 0.19 (0.06-0.38) | 4 (1-8) | 0.23 (0.08-0.46) | 1.1 (-0.94-3.19) |
| Estonia | 1 (0-1) | 0.04 (0.02-0.07) | 6 (2-11) | 0.16 (0.06-0.32) | 5.53 (4.59-6.48) |
| Eswatini | 1 (0-2) | 0.41 (0.15-0.76) | 2 (1-4) | 0.43 (0.15-0.83) | 0.81 (-0.72-2.37) |
| Ethiopia | 153 (53-291) | 1.07 (0.36-1.97) | 245 (86-459) | 0.74 (0.26-1.4) | -1.42 (-3.13-0.31) |
| Fiji | 3 (1-5) | 0.99 (0.39-1.66) | 13 (5-22) | 2.14 (0.88-3.77) | 2.98 (1.88-4.1) |
| Finland | 7 (3-11) | 0.09 (0.04-0.16) | 16 (6-29) | 0.09 (0.03-0.16) | 1.97 (0.92-3.02) |
| France | 196 (74-354) | 0.22 (0.08-0.39) | 362 (135-642) | 0.17 (0.06-0.29) | 0.8 (-0.3-1.9) |
| Gabon | 2 (1-4) | 0.47 (0.16-0.92) | 4 (1-9) | 0.62 (0.17-1.24) | 0.43 (-0.8-1.67) |
| Gambia | 1 (0-2) | 0.34 (0.13-0.63) | 2 (1-4) | 0.28 (0.09-0.55) | -0.45 (-2.01-1.14) |
| Georgia | 2 (1-3) | 0.03 (0.01-0.06) | 5 (2-10) | 0.09 (0.03-0.15) | 4.31 (3.55-5.07) |
| Germany | 176 (66-328) | 0.13 (0.05-0.23) | 354 (138-696) | 0.14 (0.05-0.26) | 2.05 (0.89-3.21) |
| Ghana | 21 (8-40) | 0.48 (0.19-0.9) | 79 (31-151) | 0.66 (0.25-1.22) | 1.51 (-0.03-3.07) |
| Greece | 79 (31-138) | 0.56 (0.22-0.99) | 154 (60-264) | 0.45 (0.18-0.75) | 0.12 (-1.11-1.36) |
| Greenland | 0 (0-0) | 0.27 (0.1-0.51) | 0 (0-0) | 0.18 (0.06-0.32) | 0.53 (-0.4-1.47) |
| Grenada | 1 (0-1) | 0.78 (0.3-1.33) | 1 (0-2) | 1.15 (0.46-1.99) | 2.06 (1.69-2.43) |
| Guam | 0 (0-1) | 0.82 (0.31-1.4) | 1 (0-2) | 0.49 (0.19-0.87) | 1.41 (0.69-2.13) |
| Guatemala | 11 (4-20) | 0.5 (0.18-0.89) | 34 (13-64) | 0.36 (0.13-0.66) | 0.95 (-0.55-2.47) |
| Guinea | 6 (2-12) | 0.22 (0.08-0.43) | 7 (2-14) | 0.15 (0.05-0.31) | -1.59 (-2.73--0.43) |
| Guinea-Bissau | 2 (1-3) | 0.54 (0.2-1.02) | 2 (1-4) | 0.34 (0.13-0.63) | -1.87 (-3.55--0.17) |
| Guyana | 2 (1-4) | 0.66 (0.26-1.15) | 7 (3-12) | 1.11 (0.45-1.98) | 3.93 (3-4.88) |
| Haiti | 19 (6-39) | 0.72 (0.23-1.57) | 36 (11-83) | 0.59 (0.18-1.36) | -0.14 (-1.27-0.99) |
| Honduras | 3 (1-6) | 0.17 (0.06-0.32) | 13 (5-24) | 0.24 (0.09-0.43) | 1.91 (0.78-3.06) |
| Hungary | 5 (2-10) | 0.04 (0.01-0.07) | 11 (4-20) | 0.05 (0.02-0.09) | 3.29 (2.4-4.19) |
| Iceland | 0 (0-0) | 0.06 (0.02-0.11) | 0 (0-1) | 0.07 (0.03-0.12) | 1.86 (1.22-2.5) |
| India | 1016 (397-1803) | 0.28 (0.11-0.48) | 2844 (1088-5036) | 0.27 (0.1-0.48) | 0.86 (0.02-1.7) |
| Indonesia | 398 (156-726) | 0.51 (0.2-0.93) | 1034 (405-1924) | 0.53 (0.2-0.97) | 0.81 (-0.04-1.67) |
| Iran (Islamic Republic of) | 84 (31-143) | 0.43 (0.16-0.76) | 190 (77-340) | 0.28 (0.11-0.5) | -0.09 (-1.03-0.87) |
| Iraq | 65 (26-112) | 0.86 (0.33-1.47) | 131 (47-251) | 0.64 (0.24-1.21) | -0.76 (-1.91-0.39) |
| Ireland | 8 (3-14) | 0.21 (0.09-0.38) | 14 (5-26) | 0.16 (0.06-0.3) | 0.5 (0.08-0.93) |
| Israel | 22 (9-38) | 0.52 (0.21-0.9) | 37 (14-68) | 0.25 (0.1-0.46) | -0.87 (-1.3--0.44) |
| Italy | 155 (61-276) | 0.18 (0.07-0.31) | 337 (130-626) | 0.16 (0.06-0.29) | 1.1 (-0.11-2.33) |
| Jamaica | 11 (5-19) | 0.61 (0.24-1.03) | 22 (9-39) | 0.69 (0.27-1.22) | 0.39 (-0.08-0.86) |
| Japan | 688 (266-1183) | 0.45 (0.17-0.78) | 2079 (824-3813) | 0.36 (0.15-0.63) | 1.49 (0.19-2.82) |
| Jordan | 7 (3-13) | 0.69 (0.27-1.18) | 45 (18-79) | 0.81 (0.31-1.41) | 1.17 (-0.28-2.65) |
| Kazakhstan | 6 (2-12) | 0.05 (0.02-0.09) | 13 (5-25) | 0.08 (0.03-0.16) | 1.28 (0.86-1.69) |
| Kenya | 24 (9-45) | 0.38 (0.14-0.72) | 101 (35-194) | 0.61 (0.2-1.16) | 2.08 (0.4-3.8) |
| Kiribati | 0 (0-1) | 1.43 (0.56-2.5) | 1 (0-2) | 1.91 (0.69-3.58) | 1.15 (-0.06-2.37) |
| Kuwait | 3 (1-5) | 0.45 (0.18-0.77) | 7 (3-13) | 0.29 (0.11-0.54) | -0.59 (-1.77-0.62) |
| Kyrgyzstan | 1 (0-2) | 0.04 (0.01-0.07) | 4 (1-7) | 0.09 (0.03-0.17) | 1.56 (0.64-2.49) |
| Lao People's Democratic Republic | 10 (3-20) | 0.6 (0.2-1.14) | 15 (5-29) | 0.4 (0.14-0.77) | -1.21 (-2.34--0.06) |
| Latvia | 1 (0-1) | 0.02 (0.01-0.04) | 3 (1-7) | 0.07 (0.03-0.14) | 5.61 (4.66-6.56) |
| Lebanon | 9 (3-16) | 0.47 (0.17-0.82) | 26 (10-47) | 0.4 (0.16-0.72) | 0.73 (0.35-1.12) |
| Lesotho | 1 (0-2) | 0.14 (0.04-0.27) | 1 (0-3) | 0.18 (0.06-0.38) | 1.33 (0.16-2.52) |
| Liberia | 5 (2-10) | 0.54 (0.2-1.01) | 7 (3-13) | 0.39 (0.15-0.73) | -1.57 (-2.9--0.23) |
| Libya | 7 (3-12) | 0.38 (0.14-0.69) | 23 (8-44) | 0.48 (0.17-0.93) | 2.21 (1.25-3.19) |
| Lithuania | 1 (0-2) | 0.02 (0.01-0.04) | 5 (2-8) | 0.07 (0.03-0.12) | 4.24 (3.35-5.13) |
| Luxembourg | 1 (1-3) | 0.28 (0.11-0.51) | 1 (1-3) | 0.12 (0.04-0.21) | -2.37 (-3.09--1.65) |
| Madagascar | 12 (4-24) | 0.33 (0.11-0.65) | 24 (9-46) | 0.35 (0.12-0.67) | -0.3 (-2.15-1.58) |
| Malawi | 11 (4-22) | 0.39 (0.14-0.75) | 27 (8-53) | 0.49 (0.15-0.97) | 0.42 (-1.24-2.12) |
| Malaysia | 62 (24-108) | 0.73 (0.29-1.27) | 192 (72-329) | 0.74 (0.28-1.27) | 0.64 (-0.18-1.46) |
| Maldives | 1 (0-2) | 1.53 (0.54-2.73) | 2 (1-3) | 0.65 (0.25-1.13) | -2.29 (-3.42--1.15) |
| Mali | 15 (6-28) | 0.49 (0.19-0.94) | 23 (8-45) | 0.33 (0.11-0.63) | -1.4 (-2.93-0.16) |
| Malta | 1 (0-2) | 0.29 (0.11-0.5) | 2 (1-4) | 0.16 (0.06-0.3) | 0 (-0.79-0.8) |
| Marshall Islands | 0 (0-0) | 1.32 (0.46-2.72) | 1 (0-2) | 2.79 (0.51-7.51) | 3.47 (2-4.97) |
| Mauritania | 7 (2-12) | 0.77 (0.29-1.44) | 12 (4-24) | 0.69 (0.26-1.35) | -0.61 (-1.83-0.63) |
| Mauritius | 5 (2-9) | 0.78 (0.31-1.35) | 32 (13-54) | 1.8 (0.76-3.07) | 4.75 (4.19-5.3) |
| Mexico | 166 (64-281) | 0.52 (0.2-0.89) | 781 (318-1343) | 0.66 (0.27-1.14) | 2.97 (2.07-3.88) |
| Micronesia (Federated States of) | 1 (0-2) | 2.08 (0.79-3.83) | 2 (1-3) | 2.93 (1.04-5.15) | 1.94 (0.77-3.13) |
| Monaco | 0 (0-0) | 0.17 (0.07-0.32) | 0 (0-1) | 0.2 (0.07-0.37) | 1.03 (-0.58-2.66) |
| Mongolia | 1 (0-3) | 0.14 (0.05-0.28) | 2 (1-4) | 0.12 (0.04-0.22) | -0.73 (-1.84-0.4) |
| Montenegro | 1 (0-1) | 0.1 (0.04-0.19) | 1 (0-2) | 0.1 (0.04-0.18) | 0.89 (0.48-1.31) |
| Morocco | 48 (17-98) | 0.37 (0.13-0.79) | 112 (45-202) | 0.36 (0.15-0.64) | 1 (0.31-1.7) |
| Mozambique | 11 (4-24) | 0.27 (0.09-0.56) | 25 (9-52) | 0.36 (0.11-0.74) | 0.85 (-0.95-2.69) |
| Myanmar | 46 (16-92) | 0.24 (0.08-0.44) | 81 (28-153) | 0.19 (0.07-0.37) | -0.08 (-0.82-0.68) |
| Namibia | 2 (1-3) | 0.29 (0.11-0.53) | 3 (1-6) | 0.27 (0.1-0.51) | -0.17 (-1.41-1.1) |
| Nauru | 0 (0-0) | 1.15 (0.41-2.36) | 0 (0-0) | 2.04 (0.71-3.98) | 1.99 (0.71-3.28) |
| Nepal | 19 (7-38) | 0.26 (0.1-0.48) | 55 (19-108) | 0.26 (0.09-0.51) | 1.2 (0.17-2.23) |
| Netherlands | 20 (7-38) | 0.1 (0.04-0.19) | 58 (21-108) | 0.14 (0.05-0.26) | 1.99 (1.11-2.88) |
| New Zealand | 4 (2-7) | 0.11 (0.04-0.19) | 11 (4-20) | 0.13 (0.05-0.22) | 1.43 (0.76-2.11) |
| Nicaragua | 4 (2-8) | 0.33 (0.13-0.6) | 22 (9-40) | 0.49 (0.2-0.86) | 2.93 (1.87-3.99) |
| Niger | 6 (2-11) | 0.28 (0.1-0.5) | 11 (4-22) | 0.17 (0.06-0.33) | -1.61 (-3.42-0.24) |
| Nigeria | 128 (50-232) | 0.37 (0.14-0.66) | 179 (70-330) | 0.26 (0.1-0.46) | -2.17 (-3.64--0.68) |
| Niue | 0 (0-0) | 1.1 (0.42-2.02) | 0 (0-0) | 2.33 (0.75-4.6) | 3.06 (2.79-3.34) |
| North Macedonia | 1 (0-2) | 0.07 (0.03-0.12) | 2 (1-3) | 0.06 (0.02-0.12) | 0.75 (0.46-1.04) |
| Northern Mariana Islands | 0 (0-0) | 2.09 (0.87-3.62) | 1 (0-1) | 2.21 (0.87-3.67) | 2.52 (1.05-4.01) |
| Norway | 4 (2-8) | 0.06 (0.02-0.1) | 13 (5-23) | 0.1 (0.04-0.18) | 2.21 (1.12-3.31) |
| Oman | 3 (1-5) | 0.51 (0.19-0.95) | 9 (4-17) | 0.62 (0.24-1.13) | 1.46 (-0.11-3.04) |
| Pakistan | 224 (82-401) | 0.46 (0.17-0.82) | 466 (168-885) | 0.45 (0.17-0.85) | -0.48 (-1.62-0.67) |
| Palau | 0 (0-0) | 1.16 (0.45-2.03) | 0 (0-1) | 2.03 (0.72-3.63) | 3.06 (2.38-3.75) |
| Palestine | 5 (2-9) | 0.68 (0.26-1.19) | 9 (4-17) | 0.47 (0.18-0.82) | -1.13 (-2.62-0.39) |
| Panama | 3 (1-6) | 0.24 (0.09-0.43) | 15 (6-27) | 0.34 (0.12-0.6) | 2.28 (1.73-2.83) |
| Papua New Guinea | 5 (2-10) | 0.34 (0.13-0.64) | 13 (4-23) | 0.3 (0.11-0.53) | -0.4 (-1.74-0.95) |
| Paraguay | 12 (4-21) | 0.6 (0.22-1.08) | 27 (11-49) | 0.5 (0.2-0.92) | 0.48 (-0.23-1.19) |
| Peru | 97 (36-170) | 0.92 (0.34-1.61) | 260 (94-478) | 0.78 (0.28-1.44) | 0.28 (-0.33-0.89) |
| Philippines | 74 (28-131) | 0.37 (0.14-0.64) | 328 (117-603) | 0.47 (0.17-0.84) | 2.28 (1.15-3.42) |
| Poland | 62 (24-112) | 0.15 (0.06-0.27) | 49 (19-85) | 0.06 (0.02-0.11) | -2.28 (-3.01--1.54) |
| Portugal | 37 (15-67) | 0.3 (0.12-0.54) | 75 (27-139) | 0.22 (0.08-0.41) | 0.39 (-0.6-1.39) |
| Puerto Rico | 37 (16-63) | 1.12 (0.48-1.89) | 87 (36-147) | 1.02 (0.42-1.73) | 2.27 (1.46-3.07) |
| Qatar | 0 (0-1) | 0.5 (0.17-0.9) | 4 (1-7) | 0.8 (0.31-1.42) | 0.93 (-1.54-3.48) |
| Republic of Korea | 96 (37-169) | 0.52 (0.2-0.94) | 286 (103-548) | 0.32 (0.11-0.61) | 0.74 (0.09-1.4) |
| Republic of Moldova | 1 (0-1) | 0.02 (0.01-0.03) | 1 (0-3) | 0.02 (0.01-0.04) | 1.82 (1.24-2.39) |
| Romania | 10 (4-20) | 0.04 (0.02-0.08) | 24 (9-41) | 0.06 (0.02-0.1) | 3.4 (2.58-4.23) |
| Russian Federation | 76 (30-135) | 0.05 (0.02-0.08) | 184 (67-316) | 0.07 (0.03-0.13) | 2.28 (1.69-2.86) |
| Rwanda | 7 (2-14) | 0.37 (0.12-0.7) | 13 (4-25) | 0.31 (0.09-0.59) | -1.14 (-2.95-0.69) |
| Saint Kitts and Nevis | 0 (0-1) | 1.11 (0.44-1.85) | 1 (0-1) | 1.28 (0.53-2.13) | 1.64 (1.27-2.02) |
| Saint Lucia | 1 (0-1) | 0.77 (0.3-1.31) | 2 (1-3) | 0.81 (0.31-1.37) | 1.92 (1.36-2.48) |
| Saint Vincent and the Grenadines | 0 (0-1) | 0.58 (0.23-0.97) | 1 (0-2) | 0.82 (0.32-1.39) | 2.97 (2.49-3.46) |
| Samoa | 1 (0-1) | 1.12 (0.43-2) | 2 (1-4) | 1.86 (0.68-3.3) | 2.38 (1.47-3.31) |
| San Marino | 0 (0-0) | 0.15 (0.05-0.26) | 0 (0-0) | 0.07 (0.03-0.14) | 0.29 (-1.07-1.66) |
| Sao Tome and Principe | 0 (0-1) | 0.53 (0.18-1.03) | 0 (0-1) | 0.47 (0.16-0.9) | -0.74 (-1.94-0.48) |
| Saudi Arabia | 45 (18-80) | 0.9 (0.36-1.56) | 210 (78-385) | 1.31 (0.49-2.34) | 1.23 (-0.2-2.68) |
| Senegal | 15 (6-28) | 0.54 (0.2-0.99) | 25 (10-51) | 0.38 (0.14-0.76) | -1.25 (-2.52-0.05) |
| Serbia | 13 (5-24) | 0.15 (0.05-0.28) | 17 (7-29) | 0.09 (0.04-0.17) | 0.2 (-0.4-0.8) |
| Seychelles | 0 (0-1) | 0.68 (0.26-1.19) | 1 (0-2) | 0.93 (0.35-1.65) | 1.55 (1.26-1.84) |
| Sierra Leone | 6 (2-11) | 0.35 (0.12-0.63) | 7 (3-13) | 0.23 (0.08-0.44) | -2.03 (-3.32--0.73) |
| Singapore | 8 (3-14) | 0.46 (0.17-0.81) | 29 (12-51) | 0.36 (0.14-0.63) | 2.33 (1.65-3.01) |
| Slovakia | 7 (3-13) | 0.12 (0.05-0.21) | 8 (3-15) | 0.08 (0.03-0.15) | -0.19 (-0.66-0.29) |
| Slovenia | 1 (1-2) | 0.05 (0.02-0.09) | 3 (1-5) | 0.05 (0.02-0.1) | 2.1 (1.25-2.97) |
| Solomon Islands | 1 (0-3) | 1.24 (0.42-2.36) | 2 (1-4) | 0.82 (0.32-1.4) | -1.16 (-2.56-0.26) |
| Somalia | 8 (3-16) | 0.53 (0.2-1.05) | 21 (7-42) | 0.53 (0.17-1.07) | -0.04 (-2.28-2.24) |
| South Africa | 49 (18-88) | 0.27 (0.1-0.48) | 108 (43-194) | 0.27 (0.11-0.48) | 0.96 (0.17-1.76) |
| South Sudan | 10 (4-20) | 0.49 (0.18-0.97) | 18 (6-34) | 0.66 (0.22-1.28) | 0.41 (-1.32-2.16) |
| Spain | 109 (39-197) | 0.21 (0.08-0.38) | 179 (68-357) | 0.12 (0.05-0.24) | -0.24 (-1.26-0.79) |
| Sri Lanka | 20 (7-36) | 0.23 (0.08-0.43) | 48 (18-94) | 0.2 (0.08-0.39) | 0.65 (0.12-1.18) |
| Sudan | 29 (11-58) | 0.33 (0.12-0.69) | 55 (22-106) | 0.3 (0.12-0.57) | -0.6 (-1.77-0.59) |
| Suriname | 2 (1-4) | 0.87 (0.36-1.49) | 6 (2-11) | 0.96 (0.36-1.7) | 1.55 (1.11-2) |
| Sweden | 13 (5-23) | 0.07 (0.03-0.13) | 41 (15-77) | 0.14 (0.05-0.26) | 3.16 (1.89-4.44) |
| Switzerland | 11 (4-20) | 0.1 (0.04-0.18) | 24 (9-47) | 0.09 (0.04-0.18) | 1.69 (0.56-2.84) |
| Syrian Arab Republic | 34 (13-61) | 0.74 (0.28-1.35) | 61 (24-116) | 0.55 (0.22-1.01) | -0.21 (-1.54-1.14) |
| Taiwan (Province of China) | 154 (62-264) | 1.38 (0.55-2.39) | 371 (140-648) | 0.81 (0.31-1.41) | 0.55 (-0.03-1.14) |
| Tajikistan | 0 (0-1) | 0.01 (0.01-0.03) | 1 (0-1) | 0.01 (0.01-0.03) | -0.71 (-1.9-0.49) |
| Thailand | 138 (53-250) | 0.51 (0.2-0.92) | 523 (193-972) | 0.48 (0.18-0.88) | 1.49 (0.92-2.06) |
| Timor-Leste | 1 (0-1) | 0.32 (0.12-0.59) | 2 (1-3) | 0.24 (0.08-0.44) | 0.61 (-0.79-2.04) |
| Togo | 3 (1-6) | 0.34 (0.12-0.63) | 8 (3-15) | 0.33 (0.12-0.63) | 0.05 (-1.78-1.91) |
| Tokelau | 0 (0-0) | 0.77 (0.28-1.46) | 0 (0-0) | 1.29 (0.46-2.33) | 2.66 (2.39-2.93) |
| Tonga | 0 (0-0) | 0.53 (0.18-0.92) | 1 (0-1) | 0.83 (0.31-1.41) | 1.76 (1.07-2.46) |
| Trinidad and Tobago | 4 (2-7) | 0.58 (0.23-0.95) | 19 (8-33) | 1.01 (0.42-1.73) | 3.94 (3.49-4.39) |
| Tunisia | 12 (4-21) | 0.29 (0.11-0.52) | 37 (14-70) | 0.32 (0.12-0.58) | 1.83 (1.22-2.44) |
| Turkey | 212 (84-384) | 0.74 (0.29-1.32) | 410 (143-718) | 0.48 (0.17-0.84) | 0.1 (-0.47-0.66) |
| Turkmenistan | 1 (1-3) | 0.08 (0.03-0.16) | 5 (2-11) | 0.15 (0.05-0.29) | 2.18 (1.21-3.17) |
| Tuvalu | 0 (0-0) | 1.06 (0.41-1.94) | 0 (0-0) | 1.45 (0.54-2.74) | 1.51 (0.96-2.06) |
| Uganda | 16 (6-31) | 0.33 (0.12-0.63) | 42 (13-81) | 0.4 (0.13-0.76) | 0.1 (-1.81-2.04) |
| Ukraine | 1 (0-1) | 0 (0-0) | 11 (4-20) | 0.01 (0-0.02) | 13.24 (11.74-14.75) |
| United Arab Emirates | 1 (1-2) | 0.38 (0.14-0.67) | 13 (4-24) | 0.73 (0.23-1.36) | 3.51 (0.78-6.31) |
| United Kingdom | 90 (33-165) | 0.09 (0.03-0.17) | 126 (47-223) | 0.08 (0.03-0.14) | 0.92 (-0.06-1.92) |
| United Republic of Tanzania | 15 (5-30) | 0.18 (0.06-0.34) | 33 (11-68) | 0.17 (0.06-0.35) | -0.45 (-1.94-1.06) |
| United States of America | 785 (311-1370) | 0.23 (0.09-0.41) | 3323 (1393-5372) | 0.53 (0.22-0.86) | 3.29 (2.64-3.93) |
| United States Virgin Islands | 0 (0-1) | 0.43 (0.16-0.75) | 1 (0-1) | 0.44 (0.18-0.78) | 2.45 (1.94-2.97) |
| Uruguay | 10 (4-18) | 0.25 (0.1-0.45) | 14 (5-25) | 0.21 (0.08-0.38) | 0.72 (0.06-1.38) |
| Uzbekistan | 5 (2-13) | 0.05 (0.01-0.12) | 14 (5-26) | 0.06 (0.02-0.12) | -0.34 (-1.33-0.67) |
| Vanuatu | 0 (0-1) | 0.65 (0.23-1.3) | 1 (0-1) | 0.58 (0.2-1.13) | -0.01 (-1.53-1.54) |
| Venezuela (Bolivarian Republic of) | 24 (9-43) | 0.28 (0.11-0.51) | 129 (50-240) | 0.45 (0.17-0.84) | 2.69 (1.94-3.44) |
| Viet Nam | 115 (41-218) | 0.33 (0.12-0.62) | 271 (93-507) | 0.32 (0.11-0.6) | 0.94 (0.26-1.61) |
| Yemen | 19 (7-38) | 0.48 (0.17-0.97) | 35 (14-68) | 0.29 (0.11-0.59) | -1.63 (-3.15--0.08) |
| Zambia | 14 (5-28) | 0.64 (0.22-1.23) | 33 (12-64) | 0.62 (0.23-1.13) | -0.63 (-2.37-1.15) |
| Zimbabwe | 8 (3-15) | 0.24 (0.09-0.46) | 13 (5-27) | 0.24 (0.09-0.47) | 0.1 (-1.41-1.63) |

**Table S4** The number of deaths and age-standardized DALYs rate of DKD attributable to low physical activity stratified by regions and countries in 1990 and 2021 with EAPC from 1990 to 2021 globally.

| Items | 1990 | | 2021 | | 1990-2021 |
| --- | --- | --- | --- | --- | --- |
|  | Number of DALYs(95% UI) | The age-standardized DALYs rate/100000(95% UI) | Number of DALYs(95% UI) | The age-standardized DALYs rate /100000(95% UI) | EAPC(95%CI) |
| **GBD Regions** |  |  |  |  |  |
| Advanced Health System | 85090 (32745-141092) | 5.25 (2.03-8.64) | 180481 (73880-293336) | 6.14 (2.51-10.05) | 1.38 (0.78-1.99) |
| Africa | 29720 (11626-52533) | 11.16 (4.42-19.13) | 59825 (24064-102796) | 9.54 (3.85-16.28) | -0.54 (-1.68-0.62) |
| African Region | 21572 (8550-37979) | 10.33 (4.1-18.14) | 43015 (16782-75151) | 8.99 (3.55-15.34) | -0.6 (-1.83-0.64) |
| America | 50565 (20036-86464) | 8.37 (3.31-14.29) | 155423 (64231-253465) | 11.53 (4.75-18.75) | 1.87 (1.65-2.08) |
| Andean Latin America | 3458 (1328-6146) | 17.91 (6.9-31.62) | 10406 (4213-17917) | 17.77 (7.19-30.48) | 0.86 (0.24-1.48) |
| Asia | 176292 (70524-297659) | 9.56 (3.79-16.12) | 425214 (163523-727831) | 8.67 (3.31-14.75) | 0.65 (0.21-1.09) |
| Australasia | 616 (247-1059) | 2.65 (1.07-4.55) | 1565 (651-2669) | 2.93 (1.22-5.05) | 1.24 (0.7-1.79) |
| Basic Health System | 144663 (57321-243930) | 10.67 (4.25-17.81) | 365488 (144397-618977) | 10.04 (3.96-16.75) | 0.9 (0.49-1.32) |
| Caribbean | 3341 (1371-5661) | 13.23 (5.43-22.32) | 8422 (3521-14044) | 15.57 (6.53-25.95) | 1.87 (1.59-2.14) |
| Central Africa | 4142 (1713-7749) | 16.07 (6.63-29.29) | 8022 (3225-14858) | 13.08 (5.19-24.11) | -0.94 (-2.29-0.44) |
| Central Asia | 1429 (517-2576) | 3.14 (1.15-5.66) | 2361 (920-4299) | 2.91 (1.13-5.26) | -0.12 (-0.6-0.36) |
| Central Europe | 4339 (1737-7535) | 2.97 (1.16-5.13) | 4529 (1874-7740) | 2.04 (0.84-3.49) | -0.3 (-0.88-0.28) |
| Central Latin America | 7265 (2845-12523) | 9.23 (3.66-15.82) | 29276 (12171-50184) | 11.61 (4.83-19.67) | 2.43 (1.75-3.11) |
| Central Sub-Saharan Africa | 3369 (1399-6233) | 16.82 (6.89-30.66) | 6372 (2537-11901) | 12.81 (4.87-23.91) | -1.12 (-2.52-0.3) |
| Commonwealth High Income | 5358 (2026-9366) | 3.54 (1.33-6.19) | 8479 (3300-14600) | 3.14 (1.24-5.39) | 0.53 (-0.1-1.17) |
| Commonwealth Low Income | 4984 (1791-9314) | 6.1 (2.28-11.22) | 12506 (4835-23004) | 5.72 (2.19-10.49) | 0.57 (-0.58-1.74) |
| Commonwealth Middle Income | 47361 (18696-80055) | 8.03 (3.17-13.45) | 115137 (44123-198527) | 7.51 (2.85-12.92) | 0.32 (-0.41-1.05) |
| East Asia | 83184 (33459-146517) | 11.1 (4.5-19.47) | 194224 (76754-333032) | 9.27 (3.64-15.75) | 0.74 (0.35-1.13) |
| East Asia & Pacific - WB | 124919 (50468-214909) | 10.39 (4.15-17.86) | 299240 (117321-512450) | 9.21 (3.62-15.68) | 0.84 (0.5-1.17) |
| Eastern Africa | 7026 (2601-13031) | 10.73 (3.98-19.62) | 13812 (5154-24856) | 9.58 (3.65-16.79) | -0.58 (-1.98-0.83) |
| Eastern Europe | 5203 (2091-9395) | 1.91 (0.76-3.46) | 6963 (2574-11906) | 1.93 (0.71-3.31) | 0.5 (-0.02-1.02) |
| Eastern Mediterranean Region | 23061 (9286-39499) | 13.11 (5.26-22.23) | 53397 (22068-93197) | 11.34 (4.65-19.29) | -0.19 (-1.08-0.7) |
| Eastern Sub-Saharan Africa | 6966 (2495-13047) | 10.27 (3.71-19.03) | 13634 (4744-24874) | 9.29 (3.28-16.66) | -0.62 (-2.09-0.88) |
| Europe | 40018 (15870-68456) | 3.9 (1.53-6.77) | 55816 (22397-94263) | 3.32 (1.34-5.57) | 0.35 (-0.29-0.99) |
| Europe & Central Asia - WB | 40927 (16169-70118) | 3.88 (1.52-6.73) | 57371 (23027-97155) | 3.33 (1.35-5.59) | 0.32 (-0.26-0.89) |
| European Region | 41442 (16389-70988) | 3.91 (1.53-6.77) | 58090 (23305-98334) | 3.33 (1.35-5.6) | 0.3 (-0.27-0.88) |
| High-income Asia Pacific | 17462 (6756-29128) | 9.15 (3.59-15.22) | 37684 (15014-64978) | 6.93 (2.72-11.72) | 0.87 (-0.04-1.78) |
| High-income North America | 20601 (8335-34141) | 5.79 (2.34-9.53) | 67716 (27569-110283) | 10.37 (4.22-17.08) | 2.53 (1.97-3.09) |
| Latin America & Caribbean - WB | 30748 (12413-52943) | 12.08 (4.84-20.71) | 89218 (36915-146274) | 12.65 (5.22-20.7) | 1.31 (0.85-1.77) |
| Limited Health System | 61044 (23790-104910) | 8.21 (3.2-13.88) | 140621 (53555-246140) | 7.32 (2.77-12.8) | 0.13 (-0.7-0.97) |
| Middle East & North Africa - WB | 16435 (6513-28110) | 13.92 (5.49-23.5) | 39920 (16599-68463) | 11.26 (4.63-19.22) | -0.01 (-0.8-0.79) |
| Minimal Health System | 6615 (2764-11996) | 11.77 (4.89-21) | 11304 (4464-21266) | 9.59 (3.81-17.56) | -1.07 (-2.41-0.28) |
| North Africa and Middle East | 23436 (9331-39698) | 14.4 (5.74-24.36) | 51845 (21407-91074) | 11.18 (4.55-18.93) | -0.13 (-0.88-0.63) |
| North America | 20609 (8338-34154) | 5.79 (2.34-9.53) | 67729 (27574-110303) | 10.37 (4.22-17.08) | 2.53 (1.97-3.09) |
| Northern Africa | 8387 (3274-15201) | 14.4 (5.59-26) | 18165 (7466-31759) | 11.82 (4.97-20.31) | -0.04 (-0.78-0.69) |
| Oceania | 458 (169-811) | 15.41 (5.87-26.56) | 1194 (453-2058) | 16 (6.24-26.74) | 0.32 (-0.67-1.32) |
| Region of the Americas | 50565 (20036-86464) | 8.37 (3.31-14.29) | 155423 (64231-253465) | 11.53 (4.75-18.75) | 1.87 (1.65-2.08) |
| South-East Asia Region | 53709 (20609-92407) | 8.19 (3.15-13.84) | 135010 (52619-237375) | 7.62 (2.93-13.24) | 0.62 (-0.03-1.27) |
| South Asia | 40354 (15650-70024) | 7.42 (2.88-12.74) | 99470 (38020-177081) | 6.88 (2.61-12.1) | 0.47 (-0.24-1.18) |
| South Asia - WB | 42226 (16419-73052) | 7.51 (2.92-12.79) | 102412 (39233-182033) | 6.9 (2.61-12.12) | 0.42 (-0.29-1.13) |
| Southeast Asia | 23926 (9288-42203) | 10.02 (3.91-17.28) | 66648 (24531-118587) | 10.31 (3.83-18.23) | 1.05 (0.45-1.65) |
| Southern Africa | 3361 (1269-6236) | 7.93 (3-14.37) | 7317 (2885-13326) | 7.8 (3.1-13.89) | 0.13 (-0.87-1.15) |
| Southern Latin America | 4209 (1764-7671) | 9.33 (3.85-16.99) | 5253 (2171-9159) | 5.94 (2.46-10.42) | -0.58 (-0.87--0.29) |
| Southern Sub-Saharan Africa | 2197 (823-3878) | 8.09 (3.02-14.18) | 4418 (1802-7974) | 7.53 (3.03-13.43) | 0.41 (-0.29-1.12) |
| Sub-Saharan Africa - WB | 21497 (8519-37748) | 10.29 (4.1-18) | 41934 (16299-73987) | 8.89 (3.5-15.38) | -0.68 (-1.93-0.59) |
| Tropical Latin America | 12598 (4960-22305) | 14.51 (5.74-24.62) | 36162 (14638-61943) | 14.14 (5.76-24.12) | 0.9 (0.45-1.36) |
| Western Africa | 6804 (2666-11993) | 9.15 (3.56-16.04) | 12509 (4873-22200) | 7.28 (2.87-12.6) | -1.09 (-2.32-0.16) |
| Western Europe | 25424 (10218-43090) | 4.3 (1.69-7.37) | 35349 (14336-59584) | 3.35 (1.34-5.67) | 0.1 (-0.79-0.99) |
| Western Pacific Region | 102662 (41620-176317) | 9.99 (3.99-17.25) | 244054 (95876-414085) | 8.7 (3.43-14.61) | 0.83 (0.5-1.17) |
| Western Sub-Saharan Africa | 7863 (3048-13880) | 9.56 (3.72-16.69) | 14993 (5849-26870) | 7.82 (3.1-13.59) | -0.98 (-2.21-0.27) |
| World Bank High Income | 74698 (29099-123700) | 5.78 (2.26-9.53) | 165266 (68472-271679) | 6.78 (2.8-11.1) | 1.39 (0.72-2.06) |
| World Bank Low Income | 16485 (6596-29078) | 12.03 (4.76-21.41) | 28589 (11401-50583) | 9.33 (3.65-16.19) | -0.99 (-2.21-0.24) |
| World Bank Lower Middle Income | 81132 (31608-139695) | 8.25 (3.2-14.05) | 196630 (75258-338746) | 7.97 (3.02-13.65) | 0.48 (-0.23-1.2) |
| World Bank Upper Middle Income | 125094 (49726-212022) | 9.05 (3.6-15.1) | 307401 (122288-517453) | 8.92 (3.55-15) | 1.06 (0.74-1.37) |
| **Countries** |  |  |  |  |  |
| Afghanistan | 1304 (476-2459) | 18.34 (6.82-33.96) | 1658 (526-3408) | 14.98 (5.03-29.69) | -1.78 (-2.94--0.61) |
| Albania | 62 (24-115) | 3.25 (1.23-6.05) | 89 (35-161) | 2.07 (0.79-3.69) | 0.81 (0.34-1.27) |
| Algeria | 1247 (491-2355) | 11.04 (4.11-20.42) | 3462 (1487-6014) | 10.11 (4.34-17.58) | 0.71 (-0.05-1.48) |
| American Samoa | 8 (3-14) | 34.7 (13.87-60.46) | 43 (17-74) | 91.01 (37.27-152.14) | 4.58 (3.82-5.35) |
| Andorra | 3 (1-6) | 6.65 (2.55-11.95) | 6 (2-11) | 3.79 (1.43-6.71) | -0.47 (-1.01-0.07) |
| Angola | 306 (110-587) | 8.45 (3.14-15.78) | 811 (296-1563) | 7.25 (2.64-13.89) | -0.72 (-2.18-0.77) |
| Antigua and Barbuda | 8 (3-14) | 15.98 (6.37-27.57) | 21 (9-37) | 20.37 (8.51-34.86) | 1.89 (1.55-2.22) |
| Argentina | 3442 (1435-6336) | 10.91 (4.49-19.96) | 4004 (1607-7172) | 7.07 (2.87-12.81) | -0.67 (-0.95--0.39) |
| Armenia | 50 (18-90) | 1.94 (0.71-3.44) | 130 (51-231) | 3.01 (1.18-5.35) | 2.79 (2.45-3.13) |
| Australia | 483 (194-841) | 2.5 (1-4.32) | 1250 (514-2137) | 2.76 (1.13-4.72) | 1.27 (0.73-1.82) |
| Austria | 579 (211-1056) | 4.66 (1.72-8.41) | 1505 (587-2586) | 6.96 (2.75-11.79) | 2.86 (1.9-3.84) |
| Azerbaijan | 170 (65-309) | 3.55 (1.41-6.29) | 296 (111-537) | 2.9 (1.09-5.18) | -0.07 (-0.51-0.37) |
| Bahamas | 27 (11-47) | 17.43 (7.17-30.04) | 77 (30-140) | 18.51 (7.18-33.1) | 1.77 (1.34-2.21) |
| Bahrain | 19 (7-34) | 10.74 (4.25-18.09) | 108 (43-200) | 14.01 (5.49-25.07) | 0.9 (-0.42-2.24) |
| Bangladesh | 2855 (1021-5423) | 6.3 (2.28-11.84) | 6644 (2551-12434) | 4.98 (1.89-9.12) | 0.73 (-0.22-1.69) |
| Barbados | 50 (21-84) | 18.27 (7.45-30.94) | 104 (41-177) | 20.55 (8-34.97) | 1.81 (1.35-2.27) |
| Belarus | 203 (76-378) | 1.59 (0.6-3) | 299 (115-555) | 1.83 (0.7-3.37) | 1.05 (0.51-1.6) |
| Belgium | 909 (368-1585) | 5.77 (2.35-10.1) | 1030 (412-1788) | 4.02 (1.63-6.98) | 0.05 (-0.84-0.95) |
| Belize | 15 (6-26) | 16.16 (6.44-28.07) | 69 (27-116) | 22.31 (8.78-37.42) | 1.89 (1.03-2.75) |
| Benin | 87 (32-170) | 4.78 (1.73-9.4) | 186 (67-368) | 3.98 (1.42-7.86) | -0.81 (-2.24-0.64) |
| Bermuda | 10 (4-17) | 15.97 (6.35-27.5) | 17 (7-28) | 11.91 (4.7-20.49) | 0.77 (0.16-1.38) |
| Bhutan | 40 (14-73) | 16.89 (5.99-30.63) | 79 (28-139) | 12.89 (4.56-22.64) | 0.27 (-0.55-1.09) |
| Bolivia (Plurinational State of) | 719 (255-1293) | 24.09 (8.6-42.98) | 2062 (721-3870) | 23.44 (8.27-42.86) | 0.85 (0.1-1.6) |
| Bosnia and Herzegovina | 129 (47-237) | 3.31 (1.21-5.98) | 130 (54-224) | 2.07 (0.86-3.57) | -0.34 (-0.92-0.25) |
| Botswana | 47 (18-87) | 8.7 (3.29-15.83) | 112 (44-213) | 7.49 (2.84-13.88) | 0.09 (-0.9-1.09) |
| Brazil | 12333 (4843-21873) | 14.56 (5.75-24.7) | 35565 (14381-60762) | 14.23 (5.79-24.19) | 0.92 (0.47-1.37) |
| Brunei Darussalam | 14 (6-25) | 15.85 (6.26-27.16) | 42 (15-75) | 13.98 (5.46-25.42) | 1.04 (-0.12-2.22) |
| Bulgaria | 274 (108-488) | 2.36 (0.95-4.17) | 376 (142-671) | 2.74 (1.02-4.87) | 1.38 (0.63-2.13) |
| Burkina Faso | 307 (117-583) | 7.76 (2.97-14.21) | 517 (198-965) | 6 (2.34-11.28) | -1 (-2.27-0.28) |
| Burundi | 191 (69-365) | 9.1 (3.24-16.98) | 299 (97-586) | 7.38 (2.38-14.18) | -1.51 (-3.02-0.02) |
| Cabo Verde | 8 (3-15) | 3.54 (1.41-6.58) | 17 (6-31) | 3.75 (1.44-6.82) | 0.36 (-0.26-0.98) |
| Cambodia | 171 (60-335) | 4.65 (1.65-8.65) | 383 (140-771) | 3.53 (1.32-6.83) | -0.16 (-1.26-0.96) |
| Cameroon | 666 (229-1253) | 16.37 (5.68-30.02) | 1812 (699-3494) | 14.92 (5.78-27.7) | -0.33 (-1.65-1.01) |
| Canada | 886 (311-1577) | 2.73 (0.95-4.85) | 1746 (644-3193) | 2.33 (0.85-4.23) | 0.71 (0.03-1.39) |
| Central African Republic | 150 (58-292) | 13.7 (5.36-26.42) | 220 (84-434) | 9.86 (3.9-19.84) | -1.13 (-2.46-0.21) |
| Chad | 215 (76-404) | 7.96 (2.91-15.08) | 338 (124-644) | 5.83 (2.21-11.09) | -1.69 (-3--0.35) |
| Chile | 555 (223-1038) | 5.73 (2.27-10.64) | 996 (384-1701) | 3.85 (1.48-6.61) | -0.02 (-0.37-0.34) |
| China | 77436 (31175-137670) | 10.7 (4.33-18.94) | 183014 (71621-316997) | 9.05 (3.52-15.44) | 0.76 (0.37-1.15) |
| Colombia | 1581 (610-2822) | 9.09 (3.46-16.06) | 3520 (1409-6095) | 6.32 (2.53-10.95) | 0.44 (-0.06-0.93) |
| Comoros | 10 (4-20) | 5.79 (2.07-11.22) | 26 (9-51) | 5.89 (1.95-11.28) | 0.63 (-0.42-1.7) |
| Congo | 144 (48-298) | 13.81 (4.88-27.89) | 283 (99-547) | 10.15 (3.6-19.84) | -0.95 (-2.07-0.18) |
| Cook Islands | 2 (1-3) | 14.39 (5.5-24.12) | 5 (2-9) | 19.43 (8.06-33.71) | 2.47 (2.11-2.84) |
| Costa Rica | 132 (51-226) | 7.71 (3.01-13.34) | 462 (186-837) | 8.31 (3.38-15.01) | 1.61 (1.11-2.12) |
| Côte d'Ivoire | 335 (119-671) | 8.82 (3.28-17.11) | 724 (292-1358) | 6.25 (2.43-11.68) | -0.88 (-2.27-0.53) |
| Croatia | 157 (60-278) | 2.7 (1.02-4.8) | 193 (75-333) | 2.1 (0.81-3.56) | 0.15 (-0.59-0.88) |
| Cuba | 809 (346-1399) | 7.99 (3.39-13.84) | 2264 (898-3893) | 11.51 (4.63-19.66) | 2.86 (2.38-3.35) |
| Cyprus | 40 (16-69) | 7.2 (2.6-12.47) | 72 (28-127) | 4.21 (1.55-7.44) | -0.91 (-1.15--0.67) |
| Czechia | 365 (142-671) | 2.64 (1.03-4.84) | 292 (121-509) | 1.36 (0.55-2.37) | -1.48 (-2.13--0.82) |
| Democratic People's Republic of Korea | 2167 (840-3902) | 15.13 (5.81-26.64) | 3925 (1476-6882) | 12.2 (4.6-21.39) | 0.3 (0.03-0.58) |
| Democratic Republic of the Congo | 2694 (1126-5032) | 19.67 (8.19-35.62) | 4892 (1957-9291) | 14.88 (5.78-27.95) | -1.11 (-2.5-0.3) |
| Denmark | 332 (128-579) | 3.85 (1.46-6.71) | 669 (268-1146) | 5.09 (1.99-8.66) | 1.34 (0.48-2.21) |
| Djibouti | 7 (2-15) | 6.84 (2.29-13.13) | 49 (18-98) | 9.43 (3.24-18.52) | 1.82 (0.22-3.43) |
| Dominica | 8 (3-14) | 13.86 (5.48-24.64) | 14 (5-25) | 16.94 (6.55-30.47) | 1.56 (1.37-1.75) |
| Dominican Republic | 510 (186-934) | 14.61 (5.39-26.2) | 1636 (580-2919) | 16.27 (5.81-29.16) | 2.13 (1.52-2.74) |
| Ecuador | 780 (321-1399) | 15.38 (6.33-27.67) | 3028 (1190-5741) | 18.8 (7.41-35.35) | 1.61 (0.79-2.44) |
| Egypt | 5210 (1921-9531) | 22.15 (8.29-40.44) | 10002 (3974-18265) | 16.31 (6.43-29.85) | -0.73 (-1.69-0.23) |
| El Salvador | 324 (125-640) | 11.12 (4.29-21.89) | 1160 (438-2154) | 18.73 (7.04-34.12) | 2.78 (2.16-3.41) |
| Equatorial Guinea | 25 (9-49) | 14.17 (5.39-26.02) | 64 (23-124) | 13.74 (4.71-26.06) | -0.34 (-1.86-1.21) |
| Eritrea | 35 (11-71) | 3.79 (1.33-7.51) | 100 (33-213) | 4.36 (1.48-8.82) | 0.87 (-0.79-2.55) |
| Estonia | 43 (16-78) | 2.08 (0.77-3.8) | 114 (42-218) | 3.7 (1.4-6.85) | 2.73 (1.91-3.56) |
| Eswatini | 31 (12-58) | 10.93 (4.14-20.44) | 69 (25-136) | 11.61 (4.31-22.41) | 0.85 (-0.39-2.1) |
| Ethiopia | 3696 (1284-7108) | 21 (7.11-39.28) | 5104 (1728-9274) | 13.39 (4.71-25.13) | -1.82 (-3.3--0.32) |
| Fiji | 90 (35-154) | 24.04 (9.73-40.38) | 315 (125-556) | 43.57 (17.78-76.21) | 2.4 (1.64-3.17) |
| Finland | 211 (89-363) | 2.89 (1.21-5.03) | 310 (120-530) | 2.19 (0.87-3.78) | 0.57 (-0.33-1.48) |
| France | 4002 (1679-6959) | 4.57 (1.92-7.98) | 6205 (2498-10460) | 3.67 (1.47-6.24) | 0.16 (-0.75-1.08) |
| Gabon | 50 (18-95) | 9.65 (3.48-18.01) | 102 (35-210) | 11.25 (3.69-22.89) | 0.21 (-0.77-1.2) |
| Gambia | 24 (9-45) | 7.51 (2.79-13.94) | 62 (23-116) | 6.5 (2.31-12.18) | -0.4 (-1.73-0.96) |
| Georgia | 174 (58-312) | 2.86 (0.97-5.19) | 188 (72-328) | 3.22 (1.23-5.6) | 1.12 (0.58-1.65) |
| Germany | 4755 (1822-8309) | 3.64 (1.38-6.35) | 5830 (2350-10093) | 2.66 (1.05-4.59) | 0.01 (-0.96-0.99) |
| Ghana | 521 (203-970) | 9.57 (3.77-17.58) | 1932 (763-3736) | 12.66 (4.96-23.73) | 1.48 (0.24-2.74) |
| Greece | 1451 (581-2511) | 9.68 (3.85-16.68) | 2302 (957-3900) | 7.86 (3.33-13.19) | 0.14 (-0.9-1.2) |
| Greenland | 1 (1-3) | 5.46 (1.98-10) | 2 (1-4) | 3.84 (1.47-6.58) | 0.67 (0.06-1.29) |
| Grenada | 13 (5-22) | 18.14 (6.99-31.63) | 28 (11-49) | 24.36 (9.53-42.88) | 2.15 (1.8-2.49) |
| Guam | 11 (4-19) | 16.59 (6.25-28.17) | 28 (10-48) | 13.17 (5.06-22.57) | 1.59 (1.05-2.13) |
| Guatemala | 270 (90-504) | 9.41 (3.24-16.65) | 812 (306-1549) | 7.63 (2.83-14.36) | 1.11 (-0.11-2.34) |
| Guinea | 165 (63-327) | 5.29 (2-10.31) | 201 (74-404) | 3.75 (1.38-7.33) | -1.57 (-2.56--0.57) |
| Guinea-Bissau | 48 (17-92) | 12.67 (4.65-24.08) | 61 (23-122) | 8.21 (3.21-15.62) | -1.6 (-2.98--0.21) |
| Guyana | 56 (22-105) | 14.84 (5.68-27.24) | 169 (67-314) | 25.63 (10.1-47.03) | 3.9 (3.15-4.65) |
| Haiti | 513 (168-990) | 16.28 (5.33-32.7) | 1023 (313-2371) | 13.88 (4.32-30.58) | -0.04 (-0.94-0.86) |
| Honduras | 105 (39-189) | 5.26 (1.96-9.63) | 384 (149-710) | 6.08 (2.34-10.98) | 1.34 (0.39-2.29) |
| Hungary | 228 (91-404) | 1.57 (0.62-2.82) | 270 (107-462) | 1.35 (0.53-2.33) | 1.01 (0.22-1.79) |
| Iceland | 8 (3-14) | 2.58 (1-4.6) | 13 (5-22) | 2.22 (0.89-3.7) | 0.22 (-0.24-0.69) |
| India | 30972 (11907-53180) | 7.13 (2.7-12.09) | 77558 (29305-138989) | 6.67 (2.53-11.76) | 0.58 (-0.08-1.24) |
| Indonesia | 11686 (4458-21507) | 12.76 (4.93-23) | 30329 (11816-55278) | 12.77 (4.96-22.84) | 0.81 (0.16-1.46) |
| Iran (Islamic Republic of) | 2420 (920-4158) | 9.88 (3.77-17.19) | 5002 (1984-8677) | 6.39 (2.56-10.92) | -0.13 (-0.86-0.6) |
| Iraq | 1774 (728-3053) | 21.69 (8.65-37.36) | 3754 (1377-6879) | 14.78 (5.46-27.25) | -0.81 (-1.75-0.13) |
| Ireland | 267 (109-458) | 6.49 (2.6-11.26) | 389 (152-680) | 4.76 (1.87-8.27) | -0.25 (-0.63-0.13) |
| Israel | 441 (173-745) | 9.47 (3.75-16.31) | 618 (241-1048) | 4.69 (1.81-8.02) | -1.38 (-1.68--1.08) |
| Italy | 4093 (1644-7197) | 4.56 (1.8-7.94) | 5748 (2280-9910) | 3.34 (1.37-5.72) | 0 (-1.05-1.05) |
| Jamaica | 229 (91-383) | 12.72 (5.03-21.3) | 506 (191-922) | 16.2 (6.1-29.52) | 0.92 (0.47-1.38) |
| Japan | 15097 (5836-25010) | 9.2 (3.61-15.21) | 32225 (12920-55287) | 7.49 (2.91-12.53) | 1.04 (-0.07-2.17) |
| Jordan | 207 (81-371) | 15.41 (6.18-26.17) | 1096 (429-1959) | 15.6 (6.14-27.26) | 0.6 (-0.57-1.78) |
| Kazakhstan | 445 (162-786) | 3.53 (1.29-6.29) | 566 (212-1031) | 3.12 (1.17-5.61) | -0.32 (-0.6--0.05) |
| Kenya | 555 (203-1052) | 7.45 (2.79-13.93) | 2400 (880-4540) | 11.67 (4.01-22.15) | 2.09 (0.63-3.57) |
| Kiribati | 13 (5-23) | 34.53 (13.34-60.36) | 30 (11-60) | 42.59 (15.36-79.28) | 0.94 (0.05-1.83) |
| Kuwait | 87 (36-160) | 11.33 (4.73-20.1) | 200 (82-368) | 6.42 (2.5-11.64) | -1.1 (-1.92--0.26) |
| Kyrgyzstan | 101 (38-188) | 3.5 (1.3-6.56) | 179 (65-330) | 3.78 (1.4-7.04) | -0.14 (-0.79-0.52) |
| Lao People's Democratic Republic | 280 (92-547) | 13.89 (4.65-26.8) | 385 (128-758) | 8.64 (2.94-16.64) | -1.43 (-2.37--0.48) |
| Latvia | 73 (27-138) | 2.03 (0.75-3.86) | 103 (39-191) | 2.46 (0.95-4.54) | 1.6 (0.82-2.38) |
| Lebanon | 242 (88-438) | 11.07 (4.03-19.73) | 511 (214-905) | 8.29 (3.47-14.47) | -0.12 (-0.4-0.16) |
| Lesotho | 28 (9-53) | 3.65 (1.24-6.93) | 42 (15-86) | 4.31 (1.64-8.6) | 0.86 (-0.04-1.78) |
| Liberia | 148 (57-274) | 13.19 (4.99-24.07) | 230 (96-433) | 9.95 (3.99-18.63) | -1.26 (-2.39--0.12) |
| Libya | 183 (66-325) | 9.39 (3.52-16.37) | 649 (232-1249) | 11.3 (4.15-21.71) | 2.03 (1.23-2.85) |
| Lithuania | 100 (38-190) | 2.21 (0.82-4.19) | 168 (64-292) | 2.73 (1.03-4.75) | 1.58 (0.85-2.32) |
| Luxembourg | 35 (14-59) | 6.48 (2.55-10.91) | 29 (11-50) | 2.62 (1.03-4.47) | -2.78 (-3.4--2.16) |
| Madagascar | 280 (89-554) | 6.3 (2.04-12.29) | 592 (208-1168) | 6.46 (2.29-12.26) | -0.19 (-1.76-1.4) |
| Malawi | 260 (91-519) | 7.68 (2.8-14.94) | 630 (208-1268) | 9.6 (3.02-18.73) | 0.42 (-1.04-1.9) |
| Malaysia | 1701 (634-2975) | 18.69 (7.09-32.93) | 5253 (2012-8986) | 18.24 (6.95-30.8) | 0.71 (0.05-1.37) |
| Maldives | 32 (11-60) | 35.81 (12.89-63.76) | 49 (18-89) | 14.19 (5.23-25) | -2.58 (-3.49--1.65) |
| Mali | 417 (164-789) | 11.23 (4.44-21.23) | 697 (249-1322) | 8.03 (2.85-14.73) | -1.26 (-2.52-0.02) |
| Malta | 27 (11-46) | 6.64 (2.59-11.4) | 40 (16-69) | 4.01 (1.59-6.92) | -0.32 (-1.03-0.39) |
| Marshall Islands | 6 (2-11) | 32.9 (11.64-67.47) | 23 (5-60) | 63.61 (13.33-165.09) | 3.29 (2.16-4.43) |
| Mauritania | 172 (59-324) | 17.88 (6.34-32.8) | 323 (125-583) | 15.49 (5.87-27.8) | -0.67 (-1.72-0.39) |
| Mauritius | 134 (52-241) | 18.38 (7.27-32.63) | 704 (289-1200) | 38.08 (15.74-64.86) | 4.22 (3.73-4.71) |
| Mexico | 3925 (1539-6782) | 10.19 (3.94-17.37) | 18827 (7745-32712) | 14.86 (6.19-25.75) | 3.17 (2.39-3.95) |
| Micronesia (Federated States of) | 23 (9-42) | 47.74 (18.4-86.26) | 47 (17-85) | 64.41 (23.47-112.49) | 1.91 (1.02-2.82) |
| Monaco | 4 (2-6) | 4.79 (1.97-8.45) | 5 (2-9) | 4.61 (1.82-7.75) | 0.19 (-1.16-1.56) |
| Mongolia | 51 (18-100) | 5.07 (1.78-9.64) | 88 (32-163) | 4.01 (1.46-7.26) | -0.5 (-1.42-0.43) |
| Montenegro | 18 (7-32) | 3 (1.2-5.22) | 22 (9-40) | 2.35 (0.89-4.17) | 0.07 (-0.32-0.47) |
| Morocco | 1278 (485-2562) | 8.99 (3.34-17.96) | 2927 (1195-5177) | 8.38 (3.37-14.93) | 0.97 (0.43-1.52) |
| Mozambique | 245 (85-529) | 4.97 (1.71-10.63) | 601 (199-1225) | 6.46 (2.21-13.22) | 0.88 (-0.62-2.41) |
| Myanmar | 1333 (455-2666) | 5.87 (2.09-11.52) | 2146 (759-3988) | 4.49 (1.6-8.26) | -0.32 (-0.9-0.27) |
| Namibia | 55 (22-102) | 8.64 (3.49-15.74) | 106 (41-200) | 7.6 (2.99-14.04) | -0.3 (-1.3-0.7) |
| Nauru | 1 (0-3) | 27.98 (9.83-54.62) | 3 (1-5) | 46.52 (16.97-89.13) | 1.75 (0.78-2.72) |
| Nepal | 620 (226-1142) | 7.1 (2.74-13.09) | 1631 (627-3174) | 7.03 (2.67-13.49) | 0.84 (-0.01-1.69) |
| Netherlands | 588 (224-1048) | 2.9 (1.09-5.16) | 1119 (433-1943) | 2.97 (1.13-5.19) | 0.76 (0.01-1.51) |
| New Zealand | 133 (53-231) | 3.42 (1.38-5.9) | 315 (129-558) | 3.8 (1.58-6.79) | 1.15 (0.59-1.71) |
| Nicaragua | 127 (45-236) | 8.34 (2.99-14.97) | 597 (230-1080) | 12.04 (4.77-21.7) | 2.77 (1.84-3.7) |
| Niger | 186 (68-351) | 7.15 (2.61-12.97) | 375 (134-714) | 4.63 (1.62-8.7) | -1.44 (-2.95-0.09) |
| Nigeria | 3932 (1539-6917) | 9.45 (3.71-16.44) | 6389 (2476-11685) | 7.05 (2.75-12.51) | -1.61 (-2.82--0.39) |
| Niue | 1 (0-1) | 25.86 (9.51-49.1) | 1 (0-2) | 48.1 (15.72-92.69) | 2.68 (2.4-2.96) |
| North Macedonia | 48 (19-87) | 2.62 (1.04-4.72) | 63 (24-112) | 2 (0.76-3.51) | 0.12 (-0.18-0.41) |
| Northern Mariana Islands | 7 (3-12) | 42.22 (17.24-71.93) | 22 (9-38) | 44.59 (17.75-73.68) | 2.34 (1.34-3.35) |
| Norway | 181 (69-309) | 2.57 (0.98-4.44) | 292 (118-503) | 2.72 (1.1-4.63) | 0.48 (-0.33-1.29) |
| Oman | 81 (30-148) | 12.01 (4.42-22.03) | 280 (107-522) | 13.28 (5.28-23.7) | 1.32 (0.06-2.59) |
| Pakistan | 5866 (2166-10263) | 10.74 (3.97-18.69) | 13557 (4969-25638) | 10.89 (4.03-20.48) | -0.24 (-1.21-0.74) |
| Palau | 3 (1-5) | 26.5 (10.22-46.8) | 9 (3-15) | 40.86 (15.03-72.4) | 2.65 (2.23-3.07) |
| Palestine | 124 (49-218) | 14.68 (5.81-25.4) | 256 (101-459) | 9.98 (3.95-17.64) | -1.02 (-2.22-0.2) |
| Panama | 96 (37-173) | 6.6 (2.49-11.83) | 359 (133-635) | 8.05 (2.98-14.28) | 1.69 (1.23-2.15) |
| Papua New Guinea | 184 (64-353) | 9.62 (3.46-18.13) | 441 (151-805) | 7.91 (2.84-13.95) | -0.52 (-1.52-0.49) |
| Paraguay | 265 (101-473) | 12.53 (4.79-21.88) | 598 (231-1100) | 10.4 (4.03-19.07) | 0.27 (-0.38-0.93) |
| Peru | 1959 (728-3540) | 17.42 (6.48-31.01) | 5315 (1921-9726) | 15.92 (5.76-29.12) | 0.45 (-0.1-1.01) |
| Philippines | 1905 (703-3384) | 7.36 (2.76-13.1) | 8401 (2871-15780) | 10.42 (3.67-19) | 2.47 (1.57-3.39) |
| Poland | 1748 (709-3099) | 4.04 (1.63-7.09) | 1469 (587-2528) | 2.04 (0.81-3.51) | -1.65 (-2.26--1.05) |
| Portugal | 864 (354-1515) | 6.43 (2.61-11.23) | 1162 (431-2004) | 4.14 (1.53-7.12) | -0.42 (-1.3-0.48) |
| Puerto Rico | 774 (335-1324) | 22.04 (9.46-38.13) | 1492 (624-2483) | 20.23 (8.25-33.43) | 1.95 (1.26-2.64) |
| Qatar | 14 (5-27) | 10.98 (3.78-19.23) | 120 (50-221) | 15.13 (6.06-27.11) | 0.48 (-1.25-2.25) |
| Republic of Korea | 2163 (863-3802) | 8.99 (3.49-15.87) | 4841 (1775-8953) | 5.24 (1.94-9.63) | 0.31 (-0.18-0.81) |
| Republic of Moldova | 90 (35-167) | 2.17 (0.84-3.94) | 121 (46-221) | 2.02 (0.75-3.63) | 0.45 (0.05-0.85) |
| Romania | 647 (243-1155) | 2.41 (0.9-4.31) | 912 (355-1588) | 2.45 (0.95-4.26) | 1.59 (0.94-2.24) |
| Russian Federation | 3729 (1487-6635) | 2.13 (0.84-3.82) | 4975 (1799-8449) | 2.03 (0.73-3.46) | 0.22 (-0.26-0.7) |
| Rwanda | 167 (53-343) | 6.98 (2.37-13.56) | 282 (83-563) | 5.43 (1.61-10.4) | -1.4 (-2.93-0.15) |
| Saint Kitts and Nevis | 8 (3-14) | 23.9 (9.32-41.41) | 17 (7-31) | 25.57 (10.18-44.21) | 1.62 (1.26-1.97) |
| Saint Lucia | 13 (5-23) | 16.05 (6.05-27.95) | 40 (16-69) | 16.64 (6.45-28.63) | 1.84 (1.37-2.31) |
| Saint Vincent and the Grenadines | 9 (3-15) | 12.47 (4.93-21.45) | 24 (10-42) | 17.17 (6.95-30.51) | 2.8 (2.37-3.23) |
| Samoa | 23 (9-41) | 27.03 (10.41-47.42) | 60 (21-104) | 41.66 (14.45-72.64) | 2.05 (1.31-2.78) |
| San Marino | 2 (1-3) | 4.14 (1.58-7.09) | 2 (1-4) | 2.66 (1.02-4.77) | -0.04 (-1.03-0.97) |
| Sao Tome and Principe | 6 (2-11) | 9.98 (3.72-19.01) | 10 (4-19) | 9.54 (3.41-18.25) | -0.38 (-1.43-0.69) |
| Saudi Arabia | 1273 (495-2228) | 21.04 (8.52-36.77) | 6776 (2494-12623) | 29.24 (11.18-52.63) | 1.44 (0.36-2.54) |
| Senegal | 394 (148-730) | 12.53 (4.64-23.03) | 681 (270-1342) | 8.77 (3.48-17.37) | -1.23 (-2.31--0.13) |
| Serbia | 339 (128-585) | 3.33 (1.27-5.86) | 358 (139-644) | 2.14 (0.83-3.85) | -0.31 (-0.88-0.27) |
| Seychelles | 9 (4-16) | 16.62 (6.35-28.97) | 22 (8-38) | 18.97 (6.84-33.68) | 1.13 (0.88-1.39) |
| Sierra Leone | 157 (57-272) | 8.15 (2.98-14.04) | 213 (77-391) | 5.7 (2.11-10.42) | -1.56 (-2.67--0.44) |
| Singapore | 188 (68-323) | 9.25 (3.34-15.84) | 576 (225-985) | 6.83 (2.71-11.82) | 1.91 (1.38-2.44) |
| Slovakia | 205 (82-354) | 3.42 (1.37-5.88) | 214 (87-383) | 2.25 (0.9-4) | -0.46 (-0.92-0) |
| Slovenia | 49 (19-87) | 2.01 (0.79-3.58) | 73 (28-129) | 1.56 (0.62-2.74) | 0.41 (-0.31-1.13) |
| Solomon Islands | 40 (13-78) | 29.46 (10.09-55.54) | 75 (26-134) | 20.01 (7.51-34.69) | -0.99 (-2.04-0.07) |
| Somalia | 200 (69-402) | 10.4 (3.84-20.87) | 529 (163-1094) | 10.33 (3.42-20.96) | -0.04 (-1.89-1.85) |
| South Africa | 1773 (655-3093) | 8.46 (3.15-14.65) | 3647 (1505-6593) | 7.73 (3.13-13.84) | 0.45 (-0.12-1.02) |
| South Sudan | 216 (72-439) | 9.29 (3.15-18.47) | 417 (141-784) | 12.54 (4.11-23.93) | 0.52 (-1.03-2.1) |
| Spain | 2506 (951-4338) | 4.68 (1.75-8.22) | 3071 (1146-5595) | 2.73 (1.09-4.89) | -0.7 (-1.53-0.14) |
| Sri Lanka | 536 (193-1021) | 5.25 (1.92-9.91) | 1236 (432-2402) | 4.64 (1.7-8.89) | 0.61 (0.22-0.99) |
| Sudan | 972 (377-1818) | 9.91 (3.79-18.62) | 1852 (735-3429) | 8.35 (3.34-15.38) | -0.68 (-1.67-0.33) |
| Suriname | 58 (23-102) | 21.87 (8.88-38.27) | 151 (57-275) | 23.35 (8.73-41.7) | 1.26 (0.9-1.62) |
| Sweden | 388 (149-685) | 2.41 (0.91-4.22) | 694 (267-1231) | 2.81 (1.09-4.83) | 1.38 (0.35-2.43) |
| Switzerland | 346 (137-597) | 3.25 (1.3-5.59) | 480 (176-832) | 2.42 (0.91-4.14) | 0.08 (-0.76-0.94) |
| Syrian Arab Republic | 886 (348-1552) | 16.86 (6.52-29.8) | 1673 (675-3164) | 12.34 (5.09-22.87) | -0.11 (-1.18-0.97) |
| Taiwan (Province of China) | 3581 (1433-6306) | 26.04 (10.36-44.78) | 7286 (2719-12272) | 16.72 (6.16-28.49) | 0.63 (0.16-1.1) |
| Tajikistan | 54 (19-103) | 2.11 (0.77-4.05) | 85 (32-153) | 1.53 (0.58-2.69) | -1.07 (-2.1--0.03) |
| Thailand | 3450 (1275-6320) | 10.8 (4.14-19.23) | 11371 (4219-20994) | 10.34 (3.88-19.03) | 1.43 (0.97-1.89) |
| Timor-Leste | 16 (5-30) | 6.63 (2.41-12.08) | 43 (14-79) | 5.23 (1.69-9.57) | 0.5 (-0.63-1.64) |
| Togo | 76 (28-151) | 7.2 (2.67-13.59) | 223 (85-422) | 6.82 (2.64-12.42) | 0.13 (-1.4-1.68) |
| Tokelau | 0 (0-0) | 18.35 (6.64-34.53) | 0 (0-1) | 26.86 (9.91-46.82) | 2.05 (1.84-2.27) |
| Tonga | 9 (3-15) | 14.79 (5.37-25.49) | 16 (6-27) | 19.76 (7.36-33.36) | 1.02 (0.44-1.6) |
| Trinidad and Tobago | 111 (46-187) | 13.51 (5.49-22.71) | 471 (191-815) | 24.04 (9.76-41.86) | 3.74 (3.34-4.13) |
| Tunisia | 297 (114-534) | 6.28 (2.36-11.17) | 802 (301-1465) | 6.26 (2.34-11.49) | 1.29 (0.84-1.75) |
| Turkey | 5189 (2045-9250) | 15.85 (6.25-27.97) | 9073 (3386-16042) | 9.83 (3.64-17.2) | -0.17 (-0.62-0.29) |
| Turkmenistan | 79 (29-145) | 4.28 (1.57-7.87) | 198 (72-388) | 4.99 (1.84-9.73) | 1.27 (0.48-2.08) |
| Tuvalu | 2 (1-3) | 25.89 (9.78-47.75) | 3 (1-6) | 32.71 (11.83-60.69) | 1.09 (0.75-1.43) |
| Uganda | 367 (120-709) | 6.44 (2.17-12.2) | 955 (304-1868) | 7.45 (2.38-14.02) | -0.04 (-1.71-1.66) |
| Ukraine | 965 (365-1828) | 1.38 (0.52-2.62) | 1184 (459-2145) | 1.49 (0.58-2.69) | 0.99 (0.38-1.6) |
| United Arab Emirates | 58 (22-102) | 10.06 (3.72-17.32) | 510 (190-902) | 14.56 (4.89-26.14) | 2.38 (0.49-4.31) |
| United Kingdom | 3373 (1252-6021) | 3.72 (1.38-6.61) | 3727 (1454-6291) | 2.89 (1.14-4.97) | -0.18 (-0.91-0.57) |
| United Republic of Tanzania | 378 (137-761) | 3.77 (1.36-7.32) | 780 (277-1570) | 3.39 (1.2-6.9) | -0.61 (-1.9-0.7) |
| United States of America | 19713 (8036-32580) | 6.11 (2.48-10.06) | 65966 (26998-107662) | 11.36 (4.64-18.62) | 2.61 (2.07-3.17) |
| United States Virgin Islands | 8 (3-14) | 9.18 (3.57-16.51) | 16 (6-28) | 8.69 (3.53-15.38) | 1.84 (1.33-2.35) |
| Uruguay | 213 (79-376) | 5.41 (2.01-9.58) | 253 (95-450) | 4.42 (1.67-7.81) | 0.31 (-0.22-0.84) |
| Uzbekistan | 304 (104-566) | 2.75 (0.94-5.16) | 630 (252-1173) | 2.39 (0.96-4.29) | -0.56 (-1.33-0.22) |
| Vanuatu | 7 (3-14) | 13.58 (4.95-26.23) | 20 (7-37) | 12.19 (4.14-22.63) | 0 (-1.16-1.18) |
| Venezuela (Bolivarian Republic of) | 705 (269-1286) | 7.52 (2.88-13.63) | 3156 (1229-5913) | 10.4 (4.06-19.34) | 2.11 (1.46-2.77) |
| Viet Nam | 2637 (914-5013) | 6.85 (2.41-12.89) | 6233 (2156-11564) | 6.52 (2.23-12.13) | 0.96 (0.4-1.53) |
| Yemen | 560 (208-1057) | 11.72 (4.32-22.28) | 1084 (423-2031) | 7.38 (2.85-14.14) | -1.39 (-2.69--0.07) |
| Zambia | 354 (126-674) | 13.49 (4.75-25.68) | 857 (298-1693) | 13.19 (4.8-24.86) | -0.59 (-2.12-0.97) |
| Zimbabwe | 262 (97-493) | 6.68 (2.59-12.3) | 441 (169-879) | 6.36 (2.48-12.23) | 0.01 (-1.23-1.27) |

**Table S5** The number of deaths and age-standardized YLDs rate of DKD attributable to low physical activity stratified by regions and countries in 1990 and 2021 with EAPC from 1990 to 2021 globally.

| Items | 1990 | | | 2021 | | | | 1990-2021 | |
| --- | --- | --- | --- | --- | --- | --- | --- | --- | --- |
|  | Number of YLDs(95% UI) | The age-standardized YLDs rate/100000(95% UI) | | Number of YLDs(95% UI) | | The age-standardized YLDs rate/100000(95% UI) | | EAPC(95%CI) | |
| **GBD Regions** |  |  | |  | |  | |  | |
| Advanced Health System | 28955 (11121-50998) | 1.78 (0.68-3.11) | | 40635 (15935-69569) | | 1.49 (0.58-2.57) | | 0.15 (-0.41-0.7) | |
| Africa | 4423 (1646-7943) | 1.56 (0.58-2.82) | | 8436 (3119-15444) | | 1.2 (0.44-2.19) | | -0.78 (-1.8-0.26) | |
| African Region | 3504 (1310-6401) | 1.59 (0.58-2.94) | | 6968 (2542-12972) | | 1.29 (0.47-2.37) | | -0.69 (-1.8-0.44) | |
| America | 11281 (4261-19850) | 1.85 (0.7-3.26) | | 21265 (8407-36656) | | 1.62 (0.64-2.79) | | 0.2 (0.01-0.4) | |
| Andean Latin America | 265 (98-509) | 1.28 (0.47-2.39) | | 596 (236-1074) | | 0.99 (0.39-1.78) | | 0.27 (-0.25-0.8) | |
| Asia | 33122 (12922-58517) | 1.71 (0.67-3.03) | | 79301 (29086-139725) | | 1.57 (0.58-2.75) | | 0.95 (0.54-1.36) | |
| Australasia | 366 (139-687) | 1.56 (0.58-2.93) | | 711 (261-1322) | | 1.4 (0.51-2.61) | | 0.04 (-0.43-0.51) | |
| Basic Health System | 25026 (9630-44391) | 1.76 (0.67-3.12) | | 61006 (22830-108504) | | 1.62 (0.61-2.87) | | 1 (0.61-1.38) | |
| Caribbean | 399 (158-694) | 1.53 (0.61-2.66) | | 651 (260-1117) | | 1.21 (0.49-2.08) | | 0.19 (-0.04-0.41) | |
| Central Africa | 525 (191-949) | 1.94 (0.71-3.51) | | 1117 (405-2106) | | 1.65 (0.6-3.01) | | -0.67 (-1.93-0.6) | |
| Central Asia | 940 (334-1704) | 2.1 (0.76-3.82) | | 1073 (406-1938) | | 1.33 (0.51-2.39) | | -1.2 (-1.68--0.72) | |
| Central Europe | 1802 (664-3124) | 1.23 (0.45-2.15) | | 1923 (745-3416) | | 0.91 (0.35-1.6) | | 0.02 (-0.54-0.59) | |
| Central Latin America | 1545 (583-2727) | 1.8 (0.69-3.11) | | 3882 (1465-6983) | | 1.52 (0.57-2.72) | | 0.95 (0.4-1.51) | |
| Central Sub-Saharan Africa | 438 (158-811) | 2.07 (0.76-3.7) | | 894 (316-1674) | | 1.62 (0.58-3) | | -0.95 (-2.25-0.36) | |
| Commonwealth High Income | 2775 (1061-5112) | 1.85 (0.71-3.38) | | 3478 (1338-6149) | | 1.39 (0.53-2.48) | | -0.45 (-1.02-0.11) | |
| Commonwealth Low Income | 712 (238-1343) | 0.84 (0.28-1.58) | | 1814 (616-3397) | | 0.78 (0.27-1.44) | | 0.88 (-0.2-1.96) | |
| Commonwealth Middle Income | 9899 (3732-18021) | 1.64 (0.63-2.97) | | 21979 (8249-39200) | | 1.39 (0.53-2.47) | | 0.27 (-0.44-0.98) | |
| East Asia | 14405 (5733-25745) | 1.76 (0.69-3.14) | | 38380 (14016-69734) | | 1.74 (0.63-3.17) | | 1.64 (1.24-2.03) | |
| East Asia & Pacific - WB | 23089 (9138-40744) | 1.8 (0.7-3.18) | | 57071 (20825-102264) | | 1.71 (0.62-3.07) | | 1.3 (0.97-1.63) | |
| Eastern Africa | 468 (165-840) | 0.66 (0.23-1.18) | | 980 (360-1766) | | 0.58 (0.22-1.07) | | -0.29 (-1.53-0.97) | |
| Eastern Europe | 3530 (1353-6477) | 1.31 (0.5-2.42) | | 3418 (1297-6066) | | 0.96 (0.37-1.73) | | -0.5 (-1-0) | |
| Eastern Mediterranean Region | 2803 (1035-4903) | 1.5 (0.55-2.61) | | 5659 (2205-9963) | | 1.08 (0.43-1.87) | | -0.64 (-1.41-0.13) | |
| Eastern Sub-Saharan Africa | 319 (103-613) | 0.45 (0.15-0.86) | | 763 (262-1466) | | 0.46 (0.16-0.87) | | 0.15 (-1.24-1.56) | |
| Europe | 17167 (6405-31117) | 1.66 (0.62-2.99) | | 18937 (7098-33363) | | 1.22 (0.45-2.17) | | -0.42 (-1-0.17) | |
| Europe & Central Asia - WB | 17772 (6624-32280) | 1.67 (0.63-3.02) | | 19642 (7365-34595) | | 1.22 (0.45-2.17) | | -0.46 (-0.99-0.07) | |
| European Region | 17917 (6682-32523) | 1.67 (0.63-3.02) | | 19842 (7434-34917) | | 1.22 (0.45-2.18) | | -0.47 (-1-0.06) | |
| High-income Asia Pacific | 4362 (1559-7371) | 2.15 (0.77-3.63) | | 8645 (3278-14661) | | 1.98 (0.73-3.4) | | 1.49 (0.69-2.29) | |
| High-income North America | 6372 (2424-11184) | 1.82 (0.69-3.16) | | 10116 (4005-17407) | | 1.66 (0.65-2.87) | | 0.02 (-0.47-0.51) | |
| Latin America & Caribbean - WB | 4977 (1896-8642) | 1.84 (0.69-3.19) | | 11251 (4292-19500) | | 1.59 (0.6-2.75) | | 0.6 (0.22-0.99) | |
| Limited Health System | 11281 (4287-20327) | 1.49 (0.57-2.67) | | 24935 (9391-44457) | | 1.25 (0.47-2.22) | | 0.22 (-0.59-1.03) | |
| Middle East & North Africa - WB | 1997 (770-3470) | 1.55 (0.6-2.69) | | 4032 (1611-7117) | | 1.03 (0.42-1.77) | | -0.5 (-1.17-0.16) | |
| Minimal Health System | 802 (295-1459) | 1.39 (0.52-2.49) | | 1471 (515-2719) | | 1.15 (0.42-2.09) | | -0.97 (-2.22-0.31) | |
| North Africa and Middle East | 2868 (1112-4930) | 1.62 (0.63-2.8) | | 5374 (2145-9443) | | 1.06 (0.43-1.83) | | -0.48 (-1.14-0.18) | |
| North America | 6372 (2424-11185) | 1.82 (0.69-3.16) | | 10117 (4005-17408) | | 1.66 (0.65-2.87) | | 0.02 (-0.47-0.51) | |
| Northern Africa | 920 (343-1611) | 1.42 (0.54-2.5) | | 1567 (621-2790) | | 0.93 (0.37-1.6) | | -0.83 (-1.43--0.23) | |
| Oceania | 61 (22-111) | 1.97 (0.72-3.61) | | 125 (47-218) | | 1.56 (0.61-2.68) | | -0.51 (-1.42-0.41) | |
| Region of the Americas | 11281 (4261-19850) | 1.85 (0.7-3.26) | | 21265 (8407-36656) | | 1.62 (0.64-2.79) | | 0.2 (0.01-0.4) | |
| South-East Asia Region | 10821 (4041-19478) | 1.64 (0.63-2.96) | | 24819 (9172-44340) | | 1.38 (0.51-2.45) | | 0.47 (-0.17-1.12) | |
| South Asia | 8126 (3066-14745) | 1.48 (0.57-2.68) | | 18632 (6965-32978) | | 1.26 (0.47-2.21) | | 0.51 (-0.2-1.23) | |
| South Asia - WB | 8304 (3128-15056) | 1.46 (0.56-2.65) | | 18987 (7113-33636) | | 1.25 (0.47-2.19) | | 0.5 (-0.21-1.21) | |
| Southeast Asia | 3989 (1424-7293) | 1.67 (0.6-3.05) | | 9471 (3289-17061) | | 1.44 (0.51-2.57) | | 0.39 (-0.21-0.99) | |
| Southern Africa | 848 (305-1572) | 1.89 (0.68-3.39) | | 1489 (538-2746) | | 1.46 (0.52-2.66) | | -0.98 (-1.88--0.07) | |
| Southern Latin America | 497 (195-892) | 1.08 (0.43-1.94) | | 651 (247-1144) | | 0.77 (0.29-1.36) | | -0.16 (-0.34-0.03) | |
| Southern Sub-Saharan Africa | 741 (272-1371) | 2.63 (0.96-4.8) | | 1226 (446-2241) | | 1.98 (0.72-3.63) | | -0.74 (-1.38--0.11) | |
| Sub-Saharan Africa - WB | 3527 (1304-6418) | 1.6 (0.58-2.94) | | 6919 (2525-12874) | | 1.3 (0.47-2.39) | | -0.73 (-1.87-0.43) | |
| Tropical Latin America | 2286 (870-4038) | 2.49 (0.94-4.35) | | 5494 (2093-9512) | | 2.13 (0.81-3.68) | | 0.47 (0.08-0.86) | |
| Western Africa | 1662 (604-3143) | 2.12 (0.78-3.92) | | 3284 (1200-6236) | | 1.73 (0.62-3.23) | | -0.75 (-1.89-0.4) | |
| Western Europe | 11000 (4104-20075) | 1.92 (0.72-3.45) | | 12427 (4629-22202) | | 1.41 (0.52-2.54) | | -0.6 (-1.34-0.15) | |
| Western Pacific Region | 19043 (7603-33428) | 1.72 (0.67-3.02) | | 47800 (17688-85888) | | 1.67 (0.61-2.99) | | 1.48 (1.13-1.83) | |
| Western Sub-Saharan Africa | 1816 (657-3434) | 2.1 (0.76-3.91) | | 3682 (1346-7013) | | 1.74 (0.63-3.27) | | -0.69 (-1.83-0.47) | |
| World Bank High Income | 24767 (9500-43498) | 1.93 (0.74-3.39) | | 36304 (14315-62221) | | 1.63 (0.64-2.79) | | 0.15 (-0.44-0.75) | |
| World Bank Low Income | 1791 (651-3141) | 1.26 (0.46-2.21) | | 3298 (1161-5860) | | 0.99 (0.35-1.78) | | -0.8 (-1.93-0.35) | |
| World Bank Lower Middle Income | 15856 (5976-28680) | 1.6 (0.61-2.87) | | 33559 (12509-60543) | | 1.33 (0.5-2.39) | | 0.14 (-0.56-0.84) | |
| World Bank Upper Middle Income | 23651 (9060-42025) | 1.64 (0.62-2.9) | | 54886 (20646-97164) | | 1.56 (0.58-2.75) | | 1.11 (0.8-1.43) | |
| **Countries** |  |  | |  | |  | |  | |
| Afghanistan | 88 (32-162) | 1.3 (0.48-2.39) | | 120 (46-233) | | 0.94 (0.37-1.75) | | -1.87 (-2.94--0.8) | |
| Albania | 25 (9-47) | 1.24 (0.44-2.36) | | 37 (13-71) | | 0.87 (0.31-1.64) | | 0.66 (0.23-1.08) | |
| Algeria | 186 (70-335) | 1.42 (0.53-2.52) | | 388 (151-699) | | 1.01 (0.39-1.74) | | -0.31 (-0.89-0.27) | |
| American Samoa | 1 (0-1) | 2.84 (1.14-4.95) | | 2 (1-3) | | 3.79 (1.54-6.59) | | 2.09 (1.46-2.72) | |
| Andorra | 1 (0-2) | 2.39 (0.83-4.18) | | 3 (1-5) | | 1.65 (0.6-3.02) | | -0.36 (-0.84-0.11) | |
| Angola | 53 (18-104) | 1.37 (0.46-2.66) | | 143 (52-284) | | 1.13 (0.41-2.1) | | -0.66 (-2-0.69) | |
| Antigua and Barbuda | 1 (0-1) | 1.2 (0.45-2.2) | | 1 (0-2) | | 1.05 (0.42-1.9) | | 0.24 (0.02-0.45) | |
| Argentina | 344 (128-613) | 1.08 (0.41-1.92) | | 432 (156-773) | | 0.79 (0.28-1.42) | | -0.17 (-0.34-0) | |
| Armenia | 45 (16-84) | 1.77 (0.63-3.19) | | 61 (22-116) | | 1.42 (0.5-2.69) | | 0.46 (0.14-0.78) | |
| Australia | 309 (119-579) | 1.57 (0.6-2.95) | | 600 (223-1138) | | 1.4 (0.53-2.65) | | 0 (-0.48-0.48) | |
| Austria | 255 (88-485) | 2.07 (0.71-3.91) | | 374 (130-693) | | 2.04 (0.72-3.77) | | 0.41 (-0.38-1.19) | |
| Azerbaijan | 103 (36-194) | 2.19 (0.79-4.08) | | 143 (51-275) | | 1.42 (0.52-2.7) | | -0.98 (-1.43--0.52) | |
| Bahamas | 2 (1-4) | 1.51 (0.58-2.72) | | 5 (2-8) | | 1.07 (0.42-1.97) | | -0.14 (-0.48-0.2) | |
| Bahrain | 3 (1-6) | 1.14 (0.44-2.04) | | 14 (6-25) | | 1.17 (0.47-2.06) | | 0.57 (-0.07-1.2) | |
| Bangladesh | 518 (176-992) | 1.11 (0.38-2.15) | | 1363 (460-2561) | | 0.98 (0.33-1.81) | | 1.31 (0.41-2.22) | |
| Barbados | 5 (2-9) | 1.8 (0.72-3.27) | | 7 (3-12) | | 1.48 (0.59-2.55) | | 0.17 (-0.21-0.54) | |
| Belarus | 188 (69-355) | 1.47 (0.54-2.76) | | 231 (89-425) | | 1.42 (0.54-2.62) | | 0.48 (-0.05-1.02) | |
| Belgium | 400 (149-733) | 2.61 (0.97-4.72) | | 430 (155-770) | | 1.95 (0.7-3.53) | | -0.37 (-1.12-0.39) | |
| Belize | 1 (0-2) | 1.42 (0.52-2.59) | | 4 (1-7) | | 1.17 (0.45-2.09) | | -0.12 (-0.85-0.62) | |
| Benin | 14 (5-27) | 0.74 (0.26-1.39) | | 32 (11-63) | | 0.63 (0.22-1.19) | | -0.62 (-1.98-0.75) | |
| Bermuda | 1 (0-2) | 1.78 (0.67-3.27) | | 2 (1-3) | | 1.3 (0.5-2.25) | | 0.14 (-0.4-0.68) | |
| Bhutan | 7 (3-14) | 2.81 (1.07-5.39) | | 13 (5-24) | | 1.97 (0.76-3.7) | | -0.06 (-0.81-0.69) | |
| Bolivia (Plurinational State of) | 41 (14-80) | 1.25 (0.43-2.39) | | 92 (33-176) | | 0.97 (0.35-1.82) | | 0.1 (-0.53-0.74) | |
| Bosnia and Herzegovina | 43 (15-83) | 1.08 (0.4-2.03) | | 40 (14-76) | | 0.68 (0.25-1.27) | | -0.37 (-0.88-0.14) | |
| Botswana | 15 (5-28) | 2.68 (0.91-4.87) | | 36 (13-71) | | 2.18 (0.77-4.07) | | -0.04 (-0.94-0.87) | |
| Brazil | 2242 (857-3972) | 2.5 (0.95-4.37) | | 5422 (2064-9356) | | 2.15 (0.82-3.7) | | 0.49 (0.11-0.88) | |
| Brunei Darussalam | 2 (1-3) | 1.47 (0.51-2.63) | | 6 (2-10) | | 1.43 (0.52-2.57) | | 0.78 (0.08-1.48) | |
| Bulgaria | 140 (54-255) | 1.19 (0.46-2.15) | | 111 (42-216) | | 0.84 (0.32-1.66) | | -0.65 (-1.35-0.05) | |
| Burkina Faso | 52 (17-100) | 1.28 (0.43-2.46) | | 93 (32-173) | | 1.01 (0.35-1.92) | | -0.9 (-2.11-0.33) | |
| Burundi | 10 (3-21) | 0.48 (0.15-0.93) | | 20 (6-39) | | 0.44 (0.15-0.83) | | -0.62 (-2.03-0.81) | |
| Cabo Verde | 2 (1-4) | 1.07 (0.36-2.02) | | 4 (1-7) | | 0.75 (0.3-1.46) | | -0.78 (-1.38--0.18) | |
| Cambodia | 27 (9-54) | 0.77 (0.26-1.49) | | 58 (21-111) | | 0.53 (0.19-0.98) | | -0.35 (-1.48-0.79) | |
| Cameroon | 87 (29-168) | 2.02 (0.7-3.87) | | 280 (105-532) | | 2.1 (0.78-3.84) | | 0.35 (-0.88-1.59) | |
| Canada | 243 (88-457) | 0.75 (0.27-1.4) | | 392 (135-730) | | 0.58 (0.2-1.11) | | 0.23 (-0.4-0.86) | |
| Central African Republic | 19 (7-39) | 1.72 (0.6-3.28) | | 31 (10-62) | | 1.27 (0.44-2.46) | | -0.97 (-2.21-0.28) | |
| Chad | 42 (15-81) | 1.53 (0.54-2.91) | | 65 (24-129) | | 1.03 (0.37-2) | | -1.78 (-3.03--0.51) | |
| Chile | 113 (42-202) | 1.11 (0.42-2) | | 177 (67-309) | | 0.71 (0.26-1.25) | | 0 (-0.27-0.27) | |
| China | 13360 (5281-23962) | 1.7 (0.67-3.04) | | 36059 (13059-66068) | | 1.69 (0.61-3.11) | | 1.68 (1.28-2.09) | |
| Colombia | 419 (150-789) | 2.24 (0.81-4.17) | | 1003 (364-1861) | | 1.81 (0.66-3.36) | | 0.85 (0.42-1.29) | |
| Comoros | 1 (0-1) | 0.34 (0.11-0.68) | | 2 (1-3) | | 0.36 (0.12-0.66) | | 0.97 (0.01-1.94) | |
| Congo | 15 (5-29) | 1.37 (0.46-2.62) | | 35 (12-65) | | 1.1 (0.37-2.1) | | -0.38 (-1.38-0.63) | |
| Cook Islands | 0 (0-0) | 1.97 (0.73-3.53) | | 1 (0-1) | | 2.32 (0.94-3.9) | | 1.88 (1.55-2.2) | |
| Costa Rica | 40 (15-74) | 2.24 (0.86-4.14) | | 77 (28-141) | | 1.39 (0.51-2.52) | | -0.18 (-0.63-0.28) | |
| Côte d'Ivoire | 67 (22-130) | 1.61 (0.53-3.16) | | 152 (49-294) | | 1.2 (0.38-2.27) | | -0.66 (-1.94-0.64) | |
| Croatia | 65 (24-123) | 1.09 (0.41-2.03) | | 60 (22-109) | | 0.72 (0.26-1.29) | | -0.34 (-1.02-0.33) | |
| Cuba | 150 (59-271) | 1.46 (0.57-2.64) | | 195 (78-338) | | 1.02 (0.42-1.78) | | 0.12 (-0.28-0.53) | |
| Cyprus | 12 (4-24) | 1.57 (0.53-2.96) | | 26 (9-47) | | 1.3 (0.46-2.32) | | 0.07 (-0.22-0.35) | |
| Czechia | 154 (55-289) | 1.13 (0.41-2.14) | | 140 (54-254) | | 0.69 (0.26-1.27) | | -0.89 (-1.51--0.27) | |
| Democratic People's Republic of Korea | 422 (155-787) | 2.82 (1.07-5.16) | | 735 (245-1363) | | 2.24 (0.76-4.09) | | 0.03 (-0.21-0.26) | |
| Democratic Republic of the Congo | 342 (125-634) | 2.35 (0.87-4.29) | | 667 (231-1266) | | 1.85 (0.66-3.5) | | -0.96 (-2.26-0.36) | |
| Denmark | 179 (64-337) | 2.13 (0.77-4) | | 213 (81-405) | | 1.83 (0.68-3.45) | | -0.3 (-1.05-0.45) | |
| Djibouti | 1 (0-1) | 0.44 (0.13-0.87) | | 3 (1-5) | | 0.47 (0.16-0.87) | | 1.1 (-0.3-2.52) | |
| Dominica | 1 (0-1) | 1.01 (0.36-1.92) | | 1 (0-1) | | 0.86 (0.34-1.63) | | 0.14 (-0.05-0.34) | |
| Dominican Republic | 69 (24-126) | 1.87 (0.66-3.39) | | 146 (56-266) | | 1.43 (0.54-2.58) | | 0.23 (-0.3-0.76) | |
| Ecuador | 73 (29-141) | 1.32 (0.52-2.55) | | 166 (64-312) | | 0.99 (0.38-1.86) | | 0.22 (-0.3-0.73) | |
| Egypt | 467 (170-822) | 1.7 (0.63-2.96) | | 727 (277-1359) | | 1.01 (0.39-1.84) | | -1.53 (-2.28--0.77) | |
| El Salvador | 49 (17-95) | 1.65 (0.59-3.11) | | 71 (24-141) | | 1.16 (0.39-2.32) | | 0.63 (-0.01-1.27) | |
| Equatorial Guinea | 3 (1-6) | 1.78 (0.62-3.23) | | 8 (3-15) | | 1.47 (0.52-2.66) | | -0.92 (-2.29-0.46) | |
| Eritrea | 2 (1-5) | 0.23 (0.07-0.49) | | 6 (2-14) | | 0.24 (0.08-0.49) | | 0.69 (-0.85-2.25) | |
| Estonia | 28 (10-52) | 1.34 (0.49-2.44) | | 41 (15-76) | | 1.46 (0.54-2.67) | | 1.3 (0.55-2.06) | |
| Eswatini | 6 (2-12) | 2.06 (0.72-3.92) | | 11 (4-22) | | 1.74 (0.59-3.35) | | -0.1 (-1.19-1) | |
| Ethiopia | 82 (26-164) | 0.45 (0.14-0.91) | | 192 (63-375) | | 0.46 (0.15-0.91) | | 0.2 (-1.23-1.66) | |
| Fiji | 9 (3-16) | 2.25 (0.81-3.93) | | 20 (8-36) | | 2.59 (1.05-4.56) | | 1.27 (0.68-1.87) | |
| Finland | 112 (44-208) | 1.56 (0.61-2.88) | | 125 (48-228) | | 1.04 (0.4-1.95) | | -0.09 (-0.91-0.73) | |
| France | 1510 (570-2619) | 1.8 (0.69-3.13) | | 2227 (869-4035) | | 1.61 (0.62-2.89) | | -0.32 (-1.08-0.44) | |
| Gabon | 7 (2-13) | 1.2 (0.4-2.34) | | 10 (3-21) | | 0.95 (0.35-1.97) | | -0.84 (-1.7-0.02) | |
| Gambia | 4 (1-8) | 1.26 (0.43-2.35) | | 10 (3-20) | | 0.97 (0.32-1.87) | | -0.58 (-1.83-0.67) | |
| Georgia | 132 (42-250) | 2.18 (0.71-4.17) | | 82 (29-154) | | 1.4 (0.5-2.61) | | -1.04 (-1.54--0.54) | |
| Germany | 1982 (717-3648) | 1.57 (0.56-2.85) | | 1743 (648-3117) | | 0.98 (0.35-1.81) | | -1.65 (-2.46--0.83) | |
| Ghana | 57 (19-110) | 0.97 (0.33-1.83) | | 156 (54-294) | | 0.91 (0.31-1.65) | | 0.13 (-0.97-1.25) | |
| Greece | 333 (122-612) | 2.19 (0.8-4.03) | | 429 (163-773) | | 1.79 (0.64-3.28) | | 0.51 (-0.32-1.35) | |
| Greenland | 0 (0-1) | 1.05 (0.37-1.94) | | 1 (0-1) | | 0.83 (0.3-1.47) | | 0.61 (0.14-1.09) | |
| Grenada | 1 (0-1) | 1.18 (0.44-2.2) | | 1 (0-2) | | 1.05 (0.4-2.01) | | 0.34 (0.09-0.59) | |
| Guam | 2 (1-3) | 2.13 (0.8-3.75) | | 3 (1-6) | | 1.58 (0.59-2.86) | | 0.47 (0.02-0.93) | |
| Guatemala | 41 (14-82) | 1.26 (0.43-2.48) | | 85 (30-169) | | 0.75 (0.27-1.48) | | -0.7 (-1.68-0.29) | |
| Guinea | 39 (13-79) | 1.23 (0.43-2.42) | | 51 (17-102) | | 0.89 (0.3-1.76) | | -1.42 (-2.37--0.47) | |
| Guinea-Bissau | 6 (2-12) | 1.59 (0.52-3.06) | | 9 (3-19) | | 1.16 (0.4-2.2) | | -1.12 (-2.42-0.19) | |
| Guyana | 5 (2-9) | 1.12 (0.41-2.14) | | 7 (3-13) | | 1.05 (0.4-1.9) | | 0.72 (0.16-1.29) | |
| Haiti | 42 (14-78) | 1.28 (0.45-2.28) | | 80 (30-155) | | 1 (0.38-1.86) | | -0.39 (-1.19-0.43) | |
| Honduras | 36 (12-68) | 1.75 (0.6-3.23) | | 82 (31-160) | | 1.22 (0.47-2.31) | | -0.38 (-1.23-0.49) | |
| Hungary | 122 (47-221) | 0.84 (0.32-1.54) | | 109 (40-200) | | 0.59 (0.23-1.11) | | -0.29 (-0.97-0.39) | |
| Iceland | 5 (2-9) | 1.73 (0.65-3.13) | | 7 (3-13) | | 1.33 (0.51-2.34) | | -0.56 (-0.99--0.13) | |
| India | 6616 (2510-12061) | 1.52 (0.59-2.77) | | 15154 (5772-26896) | | 1.28 (0.48-2.26) | | 0.59 (-0.09-1.27) | |
| Indonesia | 2204 (785-4050) | 2.49 (0.9-4.51) | | 4847 (1668-8858) | | 2.03 (0.71-3.7) | | -0.08 (-0.76-0.61) | |
| Iran (Islamic Republic of) | 447 (161-785) | 1.67 (0.62-2.88) | | 899 (337-1574) | | 1.06 (0.39-1.86) | | 0.21 (-0.45-0.87) | |
| Iraq | 153 (62-277) | 1.7 (0.68-3.06) | | 325 (131-591) | | 1.1 (0.45-1.97) | | -0.77 (-1.54-0) | |
| Ireland | 146 (55-266) | 3.52 (1.32-6.41) | | 210 (80-373) | | 2.66 (0.99-4.72) | | -0.54 (-0.9--0.18) | |
| Israel | 111 (42-203) | 2.31 (0.88-4.2) | | 158 (57-283) | | 1.36 (0.49-2.43) | | -1.51 (-1.68--1.34) | |
| Italy | 1793 (680-3216) | 2.02 (0.78-3.62) | | 1928 (713-3461) | | 1.37 (0.51-2.45) | | -0.44 (-1.33-0.46) | |
| Jamaica | 22 (8-40) | 1.25 (0.46-2.26) | | 39 (15-73) | | 1.27 (0.5-2.37) | | 0.71 (0.49-0.94) | |
| Japan | 4079 (1459-6910) | 2.39 (0.85-4.05) | | 7677 (2902-13102) | | 2.37 (0.88-4.08) | | 1.61 (0.66-2.57) | |
| Jordan | 19 (7-35) | 1.23 (0.46-2.22) | | 96 (36-177) | | 1.13 (0.44-2.05) | | 0.49 (-0.44-1.43) | |
| Kazakhstan | 276 (97-511) | 2.25 (0.81-4.18) | | 248 (93-459) | | 1.36 (0.52-2.51) | | -1.4 (-1.66--1.15) | |
| Kenya | 47 (15-93) | 0.57 (0.19-1.1) | | 152 (51-295) | | 0.65 (0.22-1.26) | | 0.75 (-0.56-2.07) | |
| Kiribati | 1 (0-2) | 2.86 (1.13-5.04) | | 2 (1-4) | | 2.66 (1.06-4.51) | | 0.24 (-0.55-1.02) | |
| Kuwait | 12 (5-24) | 1.16 (0.44-2.13) | | 46 (17-85) | | 1.15 (0.45-1.98) | | 0.52 (0.06-0.98) | |
| Kyrgyzstan | 77 (27-148) | 2.67 (0.95-5.16) | | 85 (31-167) | | 1.84 (0.69-3.53) | | -1.13 (-1.75--0.5) | |
| Lao People's Democratic Republic | 24 (8-47) | 1.19 (0.41-2.31) | | 38 (13-74) | | 0.81 (0.29-1.59) | | -0.99 (-1.93--0.04) | |
| Latvia | 58 (21-110) | 1.61 (0.6-3.05) | | 52 (19-98) | | 1.31 (0.5-2.56) | | 0.02 (-0.71-0.75) | |
| Lebanon | 26 (10-48) | 1.11 (0.42-2.06) | | 54 (21-98) | | 0.89 (0.35-1.61) | | -0.05 (-0.26-0.17) | |
| Lesotho | 11 (4-21) | 1.37 (0.47-2.61) | | 9 (3-19) | | 0.94 (0.33-1.82) | | -1.52 (-2.35--0.67) | |
| Liberia | 22 (8-43) | 1.89 (0.68-3.55) | | 38 (14-74) | | 1.41 (0.53-2.67) | | -1.31 (-2.3--0.31) | |
| Libya | 23 (9-43) | 1.1 (0.4-2) | | 58 (21-107) | | 0.9 (0.34-1.59) | | 0.42 (-0.24-1.08) | |
| Lithuania | 84 (32-162) | 1.86 (0.71-3.58) | | 99 (37-177) | | 1.65 (0.62-2.97) | | 0.78 (0.06-1.5) | |
| Luxembourg | 15 (6-26) | 2.65 (0.99-4.76) | | 11 (4-21) | | 1.1 (0.4-2.01) | | -3.08 (-3.61--2.54) | |
| Madagascar | 21 (7-41) | 0.44 (0.14-0.87) | | 47 (15-92) | | 0.45 (0.15-0.87) | | -0.06 (-1.5-1.4) | |
| Malawi | 15 (4-30) | 0.41 (0.13-0.82) | | 33 (10-67) | | 0.46 (0.14-0.91) | | 0.3 (-1.09-1.72) | |
| Malaysia | 264 (96-491) | 2.82 (1.05-5.19) | | 712 (246-1308) | | 2.42 (0.84-4.39) | | 0.72 (0.11-1.33) | |
| Maldives | 3 (1-5) | 2.84 (1.05-5.05) | | 7 (3-13) | | 1.98 (0.72-3.47) | | -0.4 (-1.27-0.47) | |
| Mali | 69 (24-136) | 1.78 (0.62-3.48) | | 134 (41-265) | | 1.41 (0.43-2.72) | | -1 (-2.16-0.19) | |
| Malta | 11 (4-19) | 2.52 (0.98-4.52) | | 14 (5-25) | | 1.63 (0.66-2.9) | | -0.42 (-1.04-0.19) | |
| Marshall Islands | 0 (0-1) | 2.52 (0.92-4.6) | | 1 (0-2) | | 3.17 (1.26-5.44) | | 1.83 (0.79-2.88) | |
| Mauritania | 25 (8-47) | 2.49 (0.84-4.65) | | 52 (20-94) | | 2.33 (0.89-4.22) | | -0.16 (-1.14-0.82) | |
| Mauritius | 13 (5-25) | 1.75 (0.64-3.31) | | 43 (15-76) | | 2.34 (0.83-4.15) | | 2.59 (2.23-2.95) | |
| Mexico | 698 (269-1218) | 1.6 (0.61-2.78) | | 2023 (788-3635) | | 1.57 (0.61-2.82) | | 1.62 (1.03-2.22) | |
| Micronesia (Federated States of) | 2 (1-3) | 3.63 (1.43-6.45) | | 3 (1-4) | | 3.28 (1.39-5.58) | | 0.64 (-0.17-1.45) | |
| Monaco | 2 (1-3) | 2.4 (0.9-4.36) | | 2 (1-3) | | 1.9 (0.71-3.37) | | -0.7 (-1.86-0.46) | |
| Mongolia | 20 (7-40) | 2.03 (0.72-3.99) | | 38 (14-73) | | 1.73 (0.6-3.22) | | -0.06 (-1.01-0.91) | |
| Montenegro | 7 (2-12) | 1.09 (0.4-1.99) | | 7 (2-12) | | 0.7 (0.25-1.3) | | -0.8 (-1.17--0.43) | |
| Morocco | 171 (61-311) | 1.12 (0.41-2.02) | | 252 (95-461) | | 0.69 (0.26-1.24) | | -0.32 (-0.77-0.15) | |
| Mozambique | 21 (6-41) | 0.38 (0.12-0.75) | | 42 (13-85) | | 0.39 (0.13-0.78) | | -0.16 (-1.51-1.2) | |
| Myanmar | 179 (61-359) | 0.8 (0.28-1.6) | | 314 (107-639) | | 0.64 (0.22-1.26) | | 0.05 (-0.56-0.66) | |
| Namibia | 17 (6-33) | 2.62 (0.92-5.02) | | 30 (11-56) | | 2.02 (0.73-3.74) | | -0.55 (-1.47-0.38) | |
| Nauru | 0 (0-0) | 1.78 (0.62-3.34) | | 0 (0-0) | | 2.09 (0.74-3.65) | | 0.72 (-0.17-1.61) | |
| Nepal | 168 (61-323) | 1.9 (0.7-3.51) | | 375 (123-729) | | 1.57 (0.52-3.02) | | -0.39 (-1.2-0.43) | |
| Netherlands | 311 (111-583) | 1.55 (0.55-2.91) | | 436 (149-834) | | 1.27 (0.44-2.41) | | -0.28 (-0.95-0.38) | |
| New Zealand | 57 (21-104) | 1.46 (0.53-2.66) | | 111 (42-207) | | 1.37 (0.51-2.58) | | 0.27 (-0.16-0.7) | |
| Nicaragua | 27 (10-54) | 1.68 (0.6-3.25) | | 77 (28-150) | | 1.48 (0.55-2.79) | | 0.56 (-0.25-1.38) | |
| Niger | 47 (16-90) | 1.7 (0.58-3.2) | | 110 (36-219) | | 1.27 (0.44-2.48) | | -0.94 (-2.36-0.51) | |
| Nigeria | 1194 (433-2271) | 2.71 (0.98-5.07) | | 2337 (860-4503) | | 2.34 (0.86-4.41) | | -0.69 (-1.8-0.44) | |
| Niue | 0 (0-0) | 2.4 (0.92-4.31) | | 0 (0-0) | | 2.67 (1.06-4.58) | | 1.05 (0.79-1.31) | |
| North Macedonia | 24 (9-47) | 1.32 (0.5-2.54) | | 29 (11-53) | | 0.9 (0.34-1.66) | | -0.23 (-0.55-0.09) | |
| Northern Mariana Islands | 1 (0-1) | 3.1 (1.18-5.47) | | 1 (1-2) | | 2.33 (0.97-4.12) | | 0.87 (0.15-1.58) | |
| Norway | 117 (45-209) | 1.74 (0.67-3.12) | | 143 (55-253) | | 1.47 (0.57-2.66) | | -0.53 (-1.19-0.15) | |
| Oman | 12 (5-22) | 1.54 (0.59-2.69) | | 35 (13-62) | | 1.17 (0.45-2.03) | | 0.13 (-0.84-1.12) | |
| Pakistan | 816 (293-1462) | 1.46 (0.53-2.59) | | 1728 (653-3221) | | 1.27 (0.47-2.33) | | -0.7 (-1.58-0.19) | |
| Palau | 0 (0-0) | 2.44 (0.95-4.35) | | 1 (0-1) | | 2.52 (1.02-4.33) | | 1.25 (0.91-1.59) | |
| Palestine | 12 (4-22) | 1.31 (0.47-2.3) | | 30 (11-54) | | 0.94 (0.36-1.64) | | -0.67 (-1.61-0.27) | |
| Panama | 30 (11-57) | 2.02 (0.73-3.78) | | 57 (21-109) | | 1.3 (0.48-2.45) | | -0.51 (-0.86--0.15) | |
| Papua New Guinea | 32 (11-62) | 1.73 (0.61-3.27) | | 70 (25-128) | | 1.25 (0.46-2.25) | | -0.91 (-1.92-0.1) | |
| Paraguay | 44 (14-80) | 1.99 (0.65-3.58) | | 72 (27-131) | | 1.22 (0.45-2.22) | | -0.79 (-1.38--0.19) | |
| Peru | 151 (54-297) | 1.26 (0.46-2.42) | | 338 (128-634) | | 0.99 (0.38-1.86) | | 0.36 (-0.13-0.86) | |
| Philippines | 263 (87-490) | 0.92 (0.32-1.68) | | 795 (282-1498) | | 0.96 (0.36-1.79) | | 0.91 (0.07-1.76) | |
| Poland | 581 (221-1061) | 1.35 (0.52-2.47) | | 681 (253-1198) | | 0.98 (0.37-1.71) | | -0.08 (-0.6-0.44) | |
| Portugal | 281 (106-527) | 2.07 (0.79-3.92) | | 283 (108-511) | | 1.28 (0.48-2.33) | | -0.62 (-1.35-0.12) | |
| Puerto Rico | 66 (26-115) | 1.84 (0.73-3.21) | | 100 (43-173) | | 1.51 (0.63-2.65) | | 0.91 (0.38-1.45) | |
| Qatar | 3 (1-7) | 1.32 (0.5-2.35) | | 24 (10-46) | | 1.56 (0.63-2.69) | | 0.33 (-0.48-1.16) | |
| Republic of Korea | 254 (95-467) | 0.91 (0.34-1.64) | | 835 (303-1479) | | 0.89 (0.32-1.58) | | 2.31 (1.79-2.83) | |
| Republic of Moldova | 80 (30-150) | 1.91 (0.74-3.49) | | 94 (34-176) | | 1.59 (0.57-2.95) | | 0.1 (-0.29-0.49) | |
| Romania | 438 (162-800) | 1.62 (0.59-2.89) | | 495 (193-930) | | 1.38 (0.53-2.55) | | 0.88 (0.25-1.51) | |
| Russian Federation | 2140 (797-3821) | 1.24 (0.47-2.2) | | 1925 (707-3424) | | 0.8 (0.29-1.42) | | -0.97 (-1.44--0.5) | |
| Rwanda | 9 (3-19) | 0.36 (0.11-0.73) | | 20 (6-39) | | 0.35 (0.11-0.66) | | 0.18 (-1.24-1.62) | |
| Saint Kitts and Nevis | 0 (0-1) | 1.37 (0.48-2.59) | | 1 (0-1) | | 1.08 (0.4-1.95) | | -0.07 (-0.3-0.17) | |
| Saint Lucia | 1 (0-2) | 1.1 (0.4-2.03) | | 2 (1-4) | | 1.01 (0.39-1.75) | | 0.99 (0.64-1.34) | |
| Saint Vincent and the Grenadines | 1 (0-1) | 1.07 (0.39-1.98) | | 1 (1-3) | | 1.01 (0.38-1.88) | | 0.93 (0.6-1.26) | |
| Samoa | 2 (1-4) | 2.36 (0.85-4.4) | | 4 (2-7) | | 2.59 (1.03-4.5) | | 0.92 (0.26-1.58) | |
| San Marino | 1 (0-1) | 2.23 (0.83-4.1) | | 1 (0-2) | | 1.7 (0.62-3.01) | | -0.25 (-1.06-0.57) | |
| Sao Tome and Principe | 1 (0-1) | 1.16 (0.37-2.28) | | 1 (0-2) | | 0.93 (0.33-1.75) | | -0.72 (-1.69-0.27) | |
| Saudi Arabia | 107 (39-197) | | 1.53 (0.58-2.78) | | 362 (128-634) | | 1.3 (0.49-2.21) | | 0.16 (-0.67-0.99) |
| Senegal | 45 (17-87) | | 1.37 (0.5-2.61) | | 74 (28-144) | | 0.89 (0.33-1.72) | | -1.5 (-2.51--0.47) |
| Serbia | 79 (27-148) | | 0.71 (0.25-1.31) | | 71 (28-131) | | 0.46 (0.17-0.83) | | -0.61 (-1.14--0.08) |
| Seychelles | 1 (0-2) | | 2.06 (0.73-3.8) | | 2 (1-4) | | 1.69 (0.63-3.29) | | -0.04 (-0.31-0.23) |
| Sierra Leone | 31 (10-59) | | 1.53 (0.51-2.91) | | 46 (17-89) | | 1.16 (0.43-2.16) | | -1.3 (-2.35--0.25) |
| Singapore | 27 (10-50) | | 1.23 (0.46-2.22) | | 127 (45-230) | | 1.47 (0.52-2.68) | | 2.28 (1.84-2.72) |
| Slovakia | 69 (27-123) | | 1.16 (0.46-2.06) | | 79 (29-147) | | 0.85 (0.31-1.55) | | -0.13 (-0.57-0.31) |
| Slovenia | 26 (10-48) | | 1.06 (0.39-1.95) | | 36 (13-64) | | 0.83 (0.31-1.53) | | -0.03 (-0.7-0.64) |
| Solomon Islands | 4 (2-8) | | 3.08 (1.18-5.59) | | 7 (3-14) | | 1.94 (0.72-3.4) | | -1.28 (-2.28--0.27) |
| Somalia | 10 (3-21) | | 0.46 (0.15-0.95) | | 26 (8-54) | | 0.45 (0.14-0.92) | | -0.24 (-1.96-1.5) |
| South Africa | 612 (224-1138) | | 2.83 (1.04-5.27) | | 1040 (382-1920) | | 2.1 (0.77-3.88) | | -0.77 (-1.27--0.25) |
| South Sudan | 10 (3-21) | | 0.44 (0.14-0.86) | | 18 (6-34) | | 0.47 (0.17-0.87) | | 0.08 (-1.32-1.51) |
| Spain | 926 (325-1725) | | 1.76 (0.62-3.24) | | 1063 (375-1997) | | 1.18 (0.42-2.2) | | -0.42 (-1.09-0.26) |
| Sri Lanka | 88 (29-177) | | 0.81 (0.27-1.59) | | 228 (77-443) | | 0.84 (0.28-1.61) | | 1.1 (0.78-1.43) |
| Sudan | 199 (76-380) | | 1.93 (0.73-3.54) | | 313 (124-584) | | 1.27 (0.51-2.27) | | -1.22 (-2.1--0.34) |
| Suriname | 5 (2-9) | | 1.82 (0.67-3.36) | | 9 (3-16) | | 1.37 (0.54-2.41) | | -0.16 (-0.45-0.14) |
| Sweden | 212 (75-400) | | 1.39 (0.49-2.64) | | 224 (80-389) | | 1.09 (0.39-1.89) | | -0.27 (-1.11-0.58) |
| Switzerland | 184 (65-321) | | 1.79 (0.63-3.19) | | 205 (68-372) | | 1.24 (0.4-2.27) | | -0.85 (-1.54--0.15) |
| Syrian Arab Republic | 89 (34-166) | | 1.57 (0.58-2.86) | | 144 (55-264) | | 1.01 (0.39-1.81) | | -0.16 (-1.01-0.69) |
| Taiwan (Province of China) | 623 (228-1118) | | 3.89 (1.43-6.93) | | 1585 (558-2831) | | 3.75 (1.31-6.64) | | 1.73 (1.25-2.21) |
| Tajikistan | 46 (17-88) | | 1.81 (0.65-3.47) | | 68 (23-126) | | 1.23 (0.45-2.29) | | -1.22 (-2.26--0.17) |
| Thailand | 613 (212-1209) | | 1.85 (0.65-3.61) | | 1779 (632-3157) | | 1.62 (0.58-2.9) | | 1.33 (0.87-1.79) |
| Timor-Leste | 2 (1-4) | | 0.88 (0.31-1.69) | | 5 (2-10) | | 0.65 (0.24-1.23) | | 0.14 (-0.95-1.24) |
| Togo | 13 (5-25) | | 1.2 (0.45-2.34) | | 38 (13-72) | | 1.08 (0.37-2) | | 0.06 (-1.39-1.54) |
| Tokelau | 0 (0-0) | | 1.85 (0.7-3.32) | | 0 (0-0) | | 2 (0.79-3.45) | | 1 (0.82-1.18) |
| Tonga | 1 (1-3) | | 2.36 (0.89-4.28) | | 2 (1-3) | | 2.27 (0.9-4.09) | | 0.19 (-0.35-0.73) |
| Trinidad and Tobago | 12 (5-22) | | 1.33 (0.53-2.42) | | 26 (10-47) | | 1.38 (0.53-2.5) | | 1.2 (0.94-1.46) |
| Tunisia | 48 (17-90) | | 0.95 (0.35-1.74) | | 90 (34-162) | | 0.67 (0.25-1.2) | | 0.06 (-0.32-0.45) |
| Turkey | 704 (265-1292) | | 2 (0.77-3.63) | | 1079 (408-1934) | | 1.13 (0.43-2.02) | | -0.13 (-0.68-0.43) |
| Turkmenistan | 40 (14-77) | | 2.32 (0.82-4.48) | | 69 (25-133) | | 1.81 (0.64-3.42) | | 0.18 (-0.64-1) |
| Tuvalu | 0 (0-0) | | 2.24 (0.84-4.08) | | 0 (0-0) | | 2.14 (0.85-3.96) | | 0.1 (-0.2-0.39) |
| Uganda | 28 (9-58) | | 0.45 (0.14-0.92) | | 65 (20-129) | | 0.44 (0.14-0.85) | | -0.16 (-1.71-1.41) |
| Ukraine | 953 (358-1809) | | 1.36 (0.51-2.59) | | 977 (381-1850) | | 1.23 (0.48-2.33) | | 0.18 (-0.42-0.79) |
| United Arab Emirates | 15 (6-30) | | 1.61 (0.63-2.86) | | 131 (49-251) | | 1.45 (0.6-2.56) | | 0.36 (-0.42-1.14) |
| United Kingdom | 2092 (773-3873) | | 2.4 (0.9-4.39) | | 2160 (825-3861) | | 1.84 (0.69-3.32) | | -0.56 (-1.21-0.09) |
| United Republic of Tanzania | 45 (15-91) | | 0.42 (0.14-0.84) | | 93 (32-190) | | 0.37 (0.13-0.73) | | -0.02 (-1.24-1.21) |
| United States of America | 6128 (2336-10707) | | 1.93 (0.73-3.35) | | 9724 (3861-16791) | | 1.79 (0.71-3.1) | | 0.03 (-0.45-0.51) |
| United States Virgin Islands | 1 (0-2) | | 0.96 (0.36-1.87) | | 2 (1-3) | | 0.91 (0.37-1.65) | | 1.32 (0.82-1.83) |
| Uruguay | 39 (14-76) | | 1.02 (0.36-1.98) | | 42 (15-78) | | 0.83 (0.29-1.53) | | -0.26 (-0.68-0.16) |
| Uzbekistan | 201 (70-378) | | 1.79 (0.62-3.39) | | 279 (110-543) | | 1.05 (0.43-1.93) | | -1.31 (-2.04--0.57) |
| Vanuatu | 1 (0-2) | | 1.41 (0.53-2.66) | | 2 (1-3) | | 0.91 (0.33-1.7) | | -1.02 (-2.08-0.05) |
| Venezuela (Bolivarian Republic of) | 204 (81-390) | | 2.08 (0.81-3.99) | | 405 (147-788) | | 1.34 (0.49-2.58) | | -0.11 (-0.65-0.43) |
| Viet Nam | 302 (99-595) | | 0.77 (0.26-1.48) | | 631 (209-1234) | | 0.63 (0.21-1.2) | | 0.56 (0.01-1.11) |
| Yemen | 81 (28-146) | | 1.63 (0.6-2.92) | | 180 (65-336) | | 1.1 (0.42-2.07) | | -0.96 (-2.18-0.27) |
| Zambia | 18 (6-36) | | 0.64 (0.22-1.23) | | 45 (16-89) | | 0.61 (0.2-1.16) | | -0.17 (-1.57-1.26) |
| Zimbabwe | 80 (29-145) | | 1.92 (0.71-3.46) | | 99 (35-185) | | 1.33 (0.47-2.42) | | -1.21 (-2.33--0.08) |

**Table S6** The number of deaths and age-standardized YLLs rate of DKD attributable to low physical activity stratified by regions and countries in 1990 and 2021 with EAPC from 1990 to 2021 globally.

| Items | 1990 | | 2021 | | 1990-2021 |
| --- | --- | --- | --- | --- | --- |
|  | Number of YLLs(95% UI) | The age-standardized YLLs rate/100000(95% UI) | Number of YLLs(95% UI) | The age-standardized YLLs rate/100000(95% UI) | EAPC(95%CI) |
| GBD Regions |  |  |  |  |  |
| Advanced Health System | 56135 (22750-95689) | 3.47 (1.4-5.93) | 139845 (57487-229510) | 4.65 (1.92-7.65) | 1.89 (1.27-2.52) |
| Africa | 25296 (9863-45698) | 9.6 (3.75-16.89) | 51388 (20257-88782) | 8.34 (3.42-14.32) | -0.5 (-1.66-0.68) |
| African Region | 18068 (7017-32678) | 8.73 (3.41-15.85) | 36047 (14423-62419) | 7.7 (3.13-13.17) | -0.59 (-1.84-0.68) |
| America | 39283 (16108-67678) | 6.52 (2.66-11.19) | 134158 (54983-221211) | 9.91 (4.05-16.29) | 2.23 (2-2.46) |
| Andean Latin America | 3193 (1215-5740) | 16.64 (6.37-29.55) | 9810 (4006-16899) | 16.78 (6.85-28.78) | 0.9 (0.28-1.52) |
| Asia | 143170 (57141-246037) | 7.85 (3.12-13.22) | 345913 (137708-600668) | 7.1 (2.82-12.3) | 0.58 (0.13-1.03) |
| Australasia | 250 (101-420) | 1.1 (0.44-1.85) | 853 (321-1519) | 1.53 (0.58-2.77) | 2.53 (1.89-3.19) |
| Basic Health System | 119637 (47198-205702) | 8.92 (3.59-15.02) | 304482 (122387-514882) | 8.42 (3.4-14.17) | 0.89 (0.47-1.31) |
| Caribbean | 2942 (1214-5014) | 11.7 (4.81-19.76) | 7772 (3234-12991) | 14.36 (6-24.05) | 2.05 (1.77-2.33) |
| Central Africa | 3616 (1433-6823) | 14.13 (5.66-26.12) | 6905 (2724-13089) | 11.43 (4.43-21.11) | -0.98 (-2.34-0.41) |
| Central Asia | 489 (174-886) | 1.05 (0.37-1.9) | 1288 (516-2323) | 1.58 (0.63-2.83) | 1.07 (0.52-1.61) |
| Central Europe | 2538 (1047-4538) | 1.73 (0.7-3.1) | 2606 (1035-4547) | 1.13 (0.45-1.98) | -0.55 (-1.18-0.07) |
| Central Latin America | 5720 (2246-9957) | 7.43 (2.92-12.96) | 25394 (10646-43445) | 10.09 (4.22-17.16) | 2.71 (1.99-3.43) |
| Central Sub-Saharan Africa | 2931 (1187-5529) | 14.75 (6.12-27.19) | 5479 (2081-10420) | 11.19 (4.18-20.93) | -1.15 (-2.57-0.29) |
| Commonwealth High Income | 2582 (953-4557) | 1.68 (0.63-2.93) | 5001 (1908-8686) | 1.75 (0.68-3.01) | 1.44 (0.74-2.15) |
| Commonwealth Low Income | 4272 (1521-8167) | 5.27 (1.94-9.97) | 10692 (3935-19758) | 4.94 (1.86-8.92) | 0.52 (-0.65-1.7) |
| Commonwealth Middle Income | 37462 (15288-65157) | 6.39 (2.58-11.15) | 93158 (34536-164876) | 6.12 (2.32-10.8) | 0.33 (-0.41-1.07) |
| East Asia | 68779 (27524-122052) | 9.33 (3.75-16.45) | 155845 (61178-270350) | 7.53 (2.96-12.93) | 0.55 (0.16-0.94) |
| East Asia & Pacific - WB | 101830 (41148-177039) | 8.59 (3.44-14.7) | 242169 (97920-417930) | 7.49 (3.01-12.97) | 0.73 (0.39-1.08) |
| Eastern Africa | 6558 (2418-12205) | 10.06 (3.73-18.47) | 12831 (4746-22958) | 8.99 (3.35-15.8) | -0.6 (-2.01-0.82) |
| Eastern Europe | 1673 (634-3095) | 0.6 (0.23-1.08) | 3545 (1307-6092) | 0.97 (0.36-1.66) | 1.95 (1.35-2.55) |
| Eastern Mediterranean Region | 20258 (8099-34348) | 11.62 (4.61-19.54) | 47737 (18815-84837) | 10.26 (4.12-17.64) | -0.14 (-1.04-0.77) |
| Eastern Sub-Saharan Africa | 6647 (2370-12543) | 9.81 (3.52-18.31) | 12871 (4396-23546) | 8.82 (3.11-15.84) | -0.66 (-2.14-0.85) |
| Europe | 22852 (9243-39948) | 2.24 (0.9-3.91) | 36879 (14608-62701) | 2.1 (0.83-3.54) | 0.84 (0.17-1.51) |
| Europe & Central Asia - WB | 23155 (9361-40415) | 2.21 (0.89-3.87) | 37729 (14955-64199) | 2.1 (0.83-3.56) | 0.82 (0.22-1.43) |
| European Region | 23525 (9489-40992) | 2.23 (0.89-3.89) | 38247 (15173-65088) | 2.11 (0.83-3.57) | 0.8 (0.2-1.4) |
| High-income Asia Pacific | 13100 (4971-22278) | 7 (2.7-11.86) | 29039 (11700-51890) | 4.95 (1.95-8.53) | 0.65 (-0.3-1.61) |
| High-income North America | 14229 (5682-24291) | 3.97 (1.59-6.73) | 57599 (23357-93738) | 8.71 (3.53-14.2) | 3.31 (2.73-3.9) |
| Latin America & Caribbean - WB | 25771 (10396-44892) | 10.24 (4.08-17.76) | 77968 (31887-127979) | 11.06 (4.51-18.14) | 1.42 (0.94-1.9) |
| Limited Health System | 49763 (19882-86817) | 6.73 (2.67-11.74) | 115686 (43065-205148) | 6.07 (2.29-10.74) | 0.12 (-0.72-0.96) |
| Middle East & North Africa - WB | 14438 (5628-24712) | 12.37 (4.79-21.21) | 35888 (14839-62100) | 10.23 (4.28-17.52) | 0.05 (-0.75-0.86) |
| Minimal Health System | 5813 (2402-10791) | 10.39 (4.23-18.7) | 9832 (3930-18357) | 8.44 (3.32-15.76) | -1.09 (-2.43-0.28) |
| North Africa and Middle East | 20569 (8110-35328) | 12.78 (5.05-21.91) | 46470 (19161-81216) | 10.13 (4.11-17.48) | -0.08 (-0.85-0.69) |
| North America | 14236 (5685-24301) | 3.98 (1.59-6.73) | 57611 (23362-93757) | 8.72 (3.53-14.2) | 3.31 (2.73-3.9) |
| Northern Africa | 7467 (2826-13640) | 12.97 (4.87-23.66) | 16598 (6798-29027) | 10.89 (4.52-18.83) | 0.04 (-0.71-0.79) |
| Oceania | 397 (144-708) | 13.44 (5.02-23.29) | 1069 (415-1844) | 14.44 (5.7-24.42) | 0.42 (-0.58-1.44) |
| Region of the Americas | 39283 (16108-67678) | 6.52 (2.66-11.19) | 134158 (54983-221211) | 9.91 (4.05-16.29) | 2.23 (2-2.46) |
| South-East Asia Region | 42889 (16626-74196) | 6.55 (2.53-11.27) | 110191 (41814-196881) | 6.24 (2.37-11.18) | 0.65 (0.01-1.31) |
| South Asia | 32228 (12413-57671) | 5.95 (2.37-10.61) | 80837 (30507-143918) | 5.62 (2.13-9.88) | 0.46 (-0.26-1.18) |
| South Asia - WB | 33922 (13128-60194) | 6.05 (2.41-10.7) | 83425 (31152-148926) | 5.64 (2.12-9.98) | 0.4 (-0.31-1.11) |
| Southeast Asia | 19937 (7705-35893) | 8.35 (3.26-14.62) | 57176 (20657-102681) | 8.87 (3.33-15.75) | 1.16 (0.56-1.77) |
| Southern Africa | 2513 (935-4681) | 6.04 (2.26-11.01) | 5829 (2240-10620) | 6.34 (2.5-11.2) | 0.44 (-0.6-1.49) |
| Southern Latin America | 3713 (1543-6827) | 8.25 (3.41-15.23) | 4602 (1894-8095) | 5.18 (2.13-9.15) | -0.63 (-0.94--0.32) |
| Southern Sub-Saharan Africa | 1455 (542-2619) | 5.46 (2.03-9.7) | 3192 (1275-5961) | 5.55 (2.22-10.26) | 0.9 (0.15-1.65) |
| Sub-Saharan Africa - WB | 17970 (6961-32658) | 8.68 (3.39-15.6) | 35016 (14007-61119) | 7.59 (3.07-13.06) | -0.67 (-1.94-0.62) |
| Tropical Latin America | 10312 (4043-18437) | 12.02 (4.76-20.56) | 30668 (12386-52818) | 12.02 (4.85-20.58) | 0.99 (0.52-1.46) |
| Western Africa | 5142 (1902-9245) | 7.03 (2.61-12.77) | 9225 (3639-16961) | 5.56 (2.17-9.96) | -1.19 (-2.45-0.08) |
| Western Europe | 14423 (5720-25642) | 2.38 (0.95-4.2) | 22923 (8937-38850) | 1.93 (0.77-3.31) | 0.56 (-0.42-1.56) |
| Western Pacific Region | 83619 (33965-146360) | 8.27 (3.32-14.22) | 196254 (79743-333422) | 7.03 (2.82-12.02) | 0.69 (0.35-1.03) |
| Western Sub-Saharan Africa | 6047 (2223-10885) | 7.47 (2.75-13.45) | 11311 (4295-20657) | 6.08 (2.34-10.78) | -1.06 (-2.32-0.21) |
| World Bank High Income | 49932 (19898-85134) | 3.85 (1.54-6.55) | 128963 (53101-212433) | 5.15 (2.15-8.5) | 1.88 (1.18-2.58) |
| World Bank Low Income | 14694 (5916-26413) | 10.77 (4.39-19.08) | 25291 (10091-44748) | 8.34 (3.29-14.7) | -1.01 (-2.24-0.23) |
| World Bank Lower Middle Income | 65277 (26051-113206) | 6.65 (2.65-11.47) | 163071 (60813-284114) | 6.65 (2.49-11.49) | 0.56 (-0.16-1.28) |
| World Bank Upper Middle Income | 101443 (40204-175263) | 7.41 (2.98-12.6) | 252515 (101733-424546) | 7.36 (2.97-12.38) | 1.05 (0.73-1.37) |
| **Countries** |  |  |  |  |  |
| Afghanistan | 1216 (436-2321) | 17.04 (6.24-32.15) | 1538 (471-3235) | 14.05 (4.42-27.95) | -1.78 (-2.95--0.6) |
| Albania | 37 (13-70) | 2.02 (0.75-3.72) | 52 (20-100) | 1.2 (0.45-2.28) | 0.92 (0.41-1.43) |
| Algeria | 1060 (402-2020) | 9.62 (3.64-18.21) | 3074 (1359-5539) | 9.1 (3.93-16.18) | 0.86 (0.07-1.66) |
| American Samoa | 7 (3-13) | 31.86 (12.58-56) | 41 (16-70) | 87.22 (35.59-145.89) | 4.74 (3.97-5.51) |
| Andorra | 2 (1-4) | 4.26 (1.6-8.05) | 4 (1-7) | 2.14 (0.8-4.04) | -0.54 (-1.14-0.05) |
| Angola | 253 (87-507) | 7.07 (2.45-13.43) | 667 (243-1329) | 6.12 (2.21-11.98) | -0.73 (-2.22-0.79) |
| Antigua and Barbuda | 8 (3-13) | 14.78 (5.99-25.52) | 20 (8-35) | 19.32 (8.04-33.06) | 2 (1.66-2.35) |
| Argentina | 3098 (1283-5800) | 9.84 (4.04-18.36) | 3572 (1432-6464) | 6.28 (2.55-11.26) | -0.72 (-1.02--0.42) |
| Armenia | 5 (2-9) | 0.17 (0.07-0.32) | 69 (26-128) | 1.59 (0.6-2.96) | 7.86 (6.99-8.74) |
| Australia | 174 (67-296) | 0.92 (0.35-1.58) | 649 (242-1164) | 1.36 (0.51-2.43) | 2.88 (2.22-3.54) |
| Austria | 324 (119-588) | 2.59 (0.96-4.68) | 1131 (430-1960) | 4.91 (1.89-8.52) | 4.17 (3.08-5.26) |
| Azerbaijan | 67 (24-128) | 1.36 (0.5-2.51) | 153 (56-293) | 1.48 (0.54-2.83) | 0.94 (0.5-1.39) |
| Bahamas | 24 (10-43) | 15.92 (6.49-27.62) | 72 (28-132) | 17.44 (6.75-31) | 1.93 (1.49-2.37) |
| Bahrain | 16 (6-29) | 9.59 (3.78-16.41) | 94 (36-176) | 12.84 (4.99-23.28) | 0.95 (-0.47-2.38) |
| Bangladesh | 2337 (823-4663) | 5.19 (1.86-10.04) | 5281 (1883-10250) | 4.01 (1.43-7.62) | 0.58 (-0.38-1.55) |
| Barbados | 45 (19-76) | 16.47 (6.74-28.06) | 97 (39-165) | 19.07 (7.46-32.81) | 1.96 (1.49-2.43) |
| Belarus | 15 (6-28) | 0.12 (0.05-0.22) | 68 (26-126) | 0.41 (0.16-0.76) | 4.66 (3.85-5.49) |
| Belgium | 509 (206-907) | 3.16 (1.26-5.63) | 599 (231-1066) | 2.07 (0.81-3.65) | 0.36 (-0.64-1.38) |
| Belize | 14 (5-24) | 14.74 (5.82-25.69) | 65 (25-111) | 21.15 (8.27-35.52) | 2.04 (1.17-2.91) |
| Benin | 73 (25-143) | 4.04 (1.41-7.94) | 154 (54-311) | 3.35 (1.23-6.64) | -0.85 (-2.29-0.61) |
| Bermuda | 9 (3-15) | 14.19 (5.58-24.29) | 15 (6-26) | 10.61 (4.12-18.66) | 0.86 (0.24-1.48) |
| Bhutan | 33 (11-64) | 14.09 (4.78-26.03) | 66 (22-121) | 10.92 (3.72-19.76) | 0.33 (-0.5-1.16) |
| Bolivia (Plurinational State of) | 678 (241-1221) | 22.84 (8.13-41.37) | 1970 (689-3688) | 22.47 (7.86-41.17) | 0.88 (0.13-1.65) |
| Bosnia and Herzegovina | 86 (33-163) | 2.24 (0.85-4.13) | 90 (35-160) | 1.4 (0.55-2.46) | -0.32 (-0.95-0.32) |
| Botswana | 32 (12-62) | 6.03 (2.31-11.51) | 77 (29-153) | 5.31 (2.05-10.21) | 0.14 (-0.89-1.18) |
| Brazil | 10090 (3954-18042) | 12.06 (4.76-20.64) | 30143 (12187-51693) | 12.08 (4.88-20.6) | 1 (0.54-1.46) |
| Brunei Darussalam | 13 (5-22) | 14.38 (5.7-24.9) | 36 (13-66) | 12.55 (4.94-22.98) | 1.08 (-0.15-2.32) |
| Bulgaria | 134 (56-243) | 1.17 (0.47-2.07) | 265 (97-484) | 1.9 (0.69-3.5) | 2.75 (1.95-3.56) |
| Burkina Faso | 255 (96-498) | 6.49 (2.48-12.38) | 424 (147-814) | 4.98 (1.81-9.5) | -1.03 (-2.31-0.27) |
| Burundi | 181 (66-345) | 8.62 (3.11-16.17) | 279 (88-557) | 6.94 (2.21-13.42) | -1.56 (-3.08--0.03) |
| Cabo Verde | 6 (2-10) | 2.48 (0.92-4.6) | 13 (5-25) | 3.01 (1.13-5.66) | 0.74 (0.11-1.36) |
| Cambodia | 144 (49-284) | 3.88 (1.32-7.3) | 325 (118-675) | 3 (1.12-6.06) | -0.12 (-1.22-1) |
| Cameroon | 580 (190-1099) | 14.35 (4.71-27.11) | 1532 (552-2984) | 12.83 (4.72-24.7) | -0.43 (-1.76-0.92) |
| Canada | 642 (219-1124) | 1.99 (0.68-3.49) | 1354 (489-2532) | 1.75 (0.63-3.26) | 0.86 (0.16-1.57) |
| Central African Republic | 130 (51-257) | 11.99 (4.64-23.82) | 190 (72-377) | 8.59 (3.33-17.24) | -1.16 (-2.49-0.19) |
| Chad | 173 (62-339) | 6.43 (2.32-12.64) | 273 (97-543) | 4.8 (1.77-9.34) | -1.67 (-3--0.32) |
| Chile | 442 (177-837) | 4.61 (1.8-8.7) | 819 (312-1448) | 3.14 (1.2-5.52) | -0.01 (-0.4-0.38) |
| China | 64076 (25583-114510) | 9 (3.61-15.95) | 146954 (56488-255035) | 7.36 (2.87-12.67) | 0.57 (0.18-0.96) |
| Colombia | 1162 (449-2121) | 6.85 (2.63-12.61) | 2517 (953-4591) | 4.51 (1.72-8.22) | 0.29 (-0.23-0.81) |
| Comoros | 9 (3-19) | 5.45 (1.84-10.75) | 25 (8-49) | 5.53 (1.8-10.73) | 0.61 (-0.45-1.68) |
| Congo | 129 (42-272) | 12.44 (4.19-25.34) | 249 (85-485) | 9.05 (3.09-17.88) | -1.02 (-2.16-0.12) |
| Cook Islands | 1 (1-3) | 12.42 (4.57-21.14) | 4 (2-8) | 17.11 (6.96-30.15) | 2.56 (2.19-2.94) |
| Costa Rica | 92 (35-167) | 5.46 (2.1-9.88) | 385 (148-713) | 6.91 (2.67-12.89) | 2.16 (1.59-2.73) |
| Côte d'Ivoire | 268 (97-555) | 7.2 (2.61-14.01) | 572 (229-1096) | 5.06 (1.93-9.57) | -0.93 (-2.34-0.5) |
| Croatia | 92 (35-170) | 1.61 (0.6-2.96) | 132 (52-233) | 1.38 (0.54-2.44) | 0.43 (-0.35-1.21) |
| Cuba | 659 (280-1115) | 6.53 (2.75-11.04) | 2069 (807-3562) | 10.48 (4.15-18.02) | 3.27 (2.76-3.78) |
| Cyprus | 28 (11-49) | 5.64 (2.08-9.78) | 46 (18-81) | 2.91 (1.12-5.29) | -1.31 (-1.62--1) |
| Czechia | 211 (82-399) | 1.51 (0.58-2.87) | 152 (58-270) | 0.66 (0.25-1.18) | -1.99 (-2.69--1.29) |
| Democratic People's Republic of Korea | 1746 (637-3167) | 12.31 (4.48-22.09) | 3189 (1212-5844) | 9.96 (3.8-18.1) | 0.37 (0.08-0.65) |
| Democratic Republic of the Congo | 2352 (977-4493) | 17.32 (7.17-32.17) | 4225 (1568-8138) | 13.03 (4.96-24.75) | -1.14 (-2.54-0.29) |
| Denmark | 153 (58-288) | 1.72 (0.65-3.23) | 455 (184-771) | 3.26 (1.31-5.51) | 2.54 (1.59-3.5) |
| Djibouti | 7 (2-14) | 6.4 (2.14-12.48) | 46 (17-92) | 8.96 (3.03-17.61) | 1.86 (0.26-3.49) |
| Dominica | 7 (3-13) | 12.84 (5.12-22.64) | 13 (5-24) | 16.08 (6.25-29.16) | 1.65 (1.46-1.84) |
| Dominican Republic | 440 (160-833) | 12.74 (4.62-23.29) | 1490 (524-2726) | 14.84 (5.25-27.21) | 2.37 (1.75-2.99) |
| Ecuador | 707 (291-1261) | 14.06 (5.74-25.09) | 2862 (1101-5531) | 17.81 (6.88-34.06) | 1.7 (0.86-2.56) |
| Egypt | 4743 (1743-8782) | 20.45 (7.67-38.14) | 9275 (3664-17110) | 15.3 (5.97-28.16) | -0.67 (-1.64-0.31) |
| El Salvador | 275 (103-555) | 9.47 (3.52-19.19) | 1089 (408-2018) | 17.57 (6.56-32.3) | 3 (2.36-3.65) |
| Equatorial Guinea | 22 (8-43) | 12.4 (4.69-23.35) | 56 (18-112) | 12.27 (3.96-23.35) | -0.25 (-1.81-1.33) |
| Eritrea | 32 (10-67) | 3.56 (1.2-7.11) | 93 (30-203) | 4.12 (1.36-8.31) | 0.88 (-0.79-2.57) |
| Estonia | 15 (6-28) | 0.74 (0.27-1.38) | 73 (27-139) | 2.24 (0.83-4.21) | 4.11 (3.2-5.02) |
| Eswatini | 25 (9-48) | 8.87 (3.26-16.51) | 58 (21-121) | 9.86 (3.62-19.98) | 1.04 (-0.24-2.33) |
| Ethiopia | 3615 (1249-6938) | 20.55 (7-38.44) | 4913 (1665-8900) | 12.93 (4.52-24.29) | -1.88 (-3.36--0.37) |
| Fiji | 81 (31-139) | 21.8 (8.56-37.08) | 295 (116-524) | 40.97 (16.77-71.11) | 2.49 (1.72-3.27) |
| Finland | 98 (40-172) | 1.34 (0.54-2.33) | 185 (67-324) | 1.14 (0.42-1.99) | 1.14 (0.15-2.13) |
| France | 2492 (957-4385) | 2.77 (1.07-4.96) | 3977 (1533-7023) | 2.06 (0.83-3.63) | 0.46 (-0.56-1.48) |
| Gabon | 44 (15-84) | 8.46 (3.02-16.31) | 92 (30-195) | 10.3 (3.19-21.25) | 0.33 (-0.66-1.34) |
| Gambia | 20 (7-39) | 6.25 (2.32-12.12) | 52 (18-101) | 5.53 (1.94-10.91) | -0.36 (-1.71-1.01) |
| Georgia | 42 (14-78) | 0.68 (0.22-1.24) | 106 (40-189) | 1.81 (0.69-3.27) | 3.86 (3.15-4.58) |
| Germany | 2773 (1117-4936) | 2.07 (0.82-3.68) | 4087 (1651-7435) | 1.68 (0.66-3) | 0.99 (-0.1-2.1) |
| Ghana | 464 (182-873) | 8.6 (3.41-16.07) | 1776 (695-3408) | 11.76 (4.57-22.11) | 1.61 (0.35-2.88) |
| Greece | 1118 (447-1965) | 7.49 (2.97-13.18) | 1873 (754-3160) | 6.06 (2.51-10.21) | -0.02 (-1.19-1.16) |
| Greenland | 1 (0-2) | 4.42 (1.6-8.05) | 2 (1-3) | 3.01 (1.13-5.25) | 0.69 (0.04-1.35) |
| Grenada | 12 (5-20) | 16.96 (6.53-29.66) | 26 (10-47) | 23.3 (9.13-40.65) | 2.25 (1.9-2.61) |
| Guam | 9 (4-17) | 14.46 (5.43-25.01) | 25 (9-43) | 11.6 (4.39-19.98) | 1.76 (1.2-2.32) |
| Guatemala | 229 (78-432) | 8.15 (2.82-14.45) | 727 (268-1384) | 6.88 (2.54-12.84) | 1.35 (0.1-2.62) |
| Guinea | 126 (46-250) | 4.06 (1.52-8.07) | 150 (54-304) | 2.86 (1.03-5.74) | -1.62 (-2.62--0.6) |
| Guinea-Bissau | 41 (14-82) | 11.08 (3.95-21.21) | 51 (20-105) | 7.05 (2.73-13.89) | -1.68 (-3.06--0.27) |
| Guyana | 51 (20-96) | 13.72 (5.27-25.38) | 162 (63-303) | 24.58 (9.7-45.49) | 4.11 (3.35-4.87) |
| Haiti | 471 (154-919) | 15 (4.88-30.72) | 943 (280-2242) | 12.88 (3.9-29.17) | -0.01 (-0.91-0.9) |
| Honduras | 69 (24-128) | 3.51 (1.23-6.56) | 301 (112-558) | 4.85 (1.81-8.95) | 1.94 (0.96-2.92) |
| Hungary | 106 (42-196) | 0.72 (0.28-1.35) | 161 (64-273) | 0.76 (0.3-1.3) | 2.31 (1.39-3.22) |
| Iceland | 3 (1-5) | 0.85 (0.33-1.55) | 6 (2-11) | 0.9 (0.36-1.54) | 1.4 (0.83-1.98) |
| India | 24356 (9549-43544) | 5.61 (2.2-9.94) | 62405 (23334-113239) | 5.39 (2.03-9.63) | 0.57 (-0.09-1.24) |
| Indonesia | 9482 (3646-17958) | 10.26 (4.01-18.81) | 25482 (9821-48179) | 10.74 (4.18-19.99) | 0.99 (0.35-1.64) |
| Iran (Islamic Republic of) | 1973 (732-3484) | 8.22 (3.1-14.11) | 4103 (1629-7212) | 5.33 (2.12-9.33) | -0.19 (-0.94-0.56) |
| Iraq | 1621 (644-2811) | 19.99 (7.98-34.39) | 3429 (1194-6428) | 13.68 (4.83-25.95) | -0.82 (-1.77-0.14) |
| Ireland | 120 (47-212) | 2.97 (1.19-5.16) | 179 (69-321) | 2.09 (0.82-3.74) | 0.1 (-0.32-0.52) |
| Israel | 331 (128-567) | 7.16 (2.84-12.41) | 460 (176-831) | 3.33 (1.29-5.95) | -1.32 (-1.71--0.93) |
| Italy | 2300 (937-4015) | 2.53 (1.02-4.36) | 3820 (1436-6834) | 1.97 (0.73-3.43) | 0.27 (-0.87-1.43) |
| Jamaica | 207 (81-347) | 11.47 (4.5-19.24) | 467 (174-864) | 14.93 (5.57-27.73) | 0.95 (0.46-1.43) |
| Japan | 11017 (4156-18739) | 6.82 (2.61-11.56) | 24548 (9966-43626) | 5.11 (2-8.81) | 0.82 (-0.36-2.01) |
| Jordan | 188 (74-340) | 14.18 (5.7-24.59) | 1000 (393-1756) | 14.47 (5.78-25.1) | 0.61 (-0.58-1.82) |
| Kazakhstan | 169 (61-324) | 1.28 (0.46-2.41) | 318 (118-628) | 1.76 (0.66-3.4) | 0.76 (0.41-1.12) |
| Kenya | 508 (187-949) | 6.88 (2.58-12.91) | 2248 (826-4296) | 11.02 (3.82-21.03) | 2.19 (0.72-3.68) |
| Kiribati | 12 (4-21) | 31.67 (12.04-55.57) | 28 (10-57) | 39.93 (14.36-76.01) | 0.99 (0.1-1.89) |
| Kuwait | 75 (31-136) | 10.18 (4.3-18.5) | 154 (61-292) | 5.26 (2.05-9.77) | -1.43 (-2.34--0.52) |
| Kyrgyzstan | 25 (9-48) | 0.83 (0.29-1.59) | 94 (34-180) | 1.94 (0.7-3.73) | 1.11 (0.24-1.98) |
| Lao People's Democratic Republic | 256 (83-499) | 12.7 (4.18-24.01) | 348 (115-685) | 7.83 (2.65-15.27) | -1.47 (-2.42--0.52) |
| Latvia | 15 (6-29) | 0.42 (0.16-0.8) | 51 (19-99) | 1.15 (0.44-2.2) | 4.47 (3.58-5.36) |
| Lebanon | 216 (73-393) | 9.96 (3.41-17.94) | 457 (191-815) | 7.41 (3.09-13.3) | -0.13 (-0.42-0.16) |
| Lesotho | 17 (6-34) | 2.28 (0.75-4.37) | 33 (11-68) | 3.37 (1.14-6.85) | 1.83 (0.87-2.79) |
| Liberia | 126 (48-235) | 11.31 (4.3-20.54) | 193 (74-358) | 8.54 (3.26-16.05) | -1.25 (-2.41--0.08) |
| Libya | 160 (58-294) | 8.29 (3.06-15.14) | 591 (203-1155) | 10.4 (3.65-20.13) | 2.22 (1.39-3.05) |
| Lithuania | 16 (6-30) | 0.36 (0.14-0.66) | 69 (28-123) | 1.08 (0.43-1.98) | 3.53 (2.67-4.4) |
| Luxembourg | 21 (8-36) | 3.83 (1.51-6.83) | 18 (7-33) | 1.52 (0.6-2.75) | -2.59 (-3.28--1.9) |
| Madagascar | 259 (82-519) | 5.86 (1.91-11.57) | 545 (193-1105) | 6.01 (2.12-11.62) | -0.2 (-1.78-1.4) |
| Malawi | 246 (86-496) | 7.27 (2.64-14.29) | 598 (198-1209) | 9.14 (2.88-17.87) | 0.42 (-1.04-1.91) |
| Malaysia | 1437 (544-2547) | 15.87 (6.08-27.97) | 4542 (1705-7799) | 15.82 (5.88-27.07) | 0.71 (0.03-1.39) |
| Maldives | 30 (10-56) | 32.97 (11.58-59.77) | 41 (15-77) | 12.21 (4.56-22.2) | -2.84 (-3.76--1.91) |
| Mali | 348 (134-676) | 9.45 (3.66-18.02) | 563 (206-1133) | 6.62 (2.36-13.02) | -1.31 (-2.59--0.01) |
| Malta | 16 (6-29) | 4.12 (1.6-7.19) | 26 (10-47) | 2.38 (0.9-4.23) | -0.26 (-1.03-0.52) |
| Marshall Islands | 5 (2-11) | 30.38 (10.55-63.61) | 22 (4-59) | 60.45 (11.85-160.27) | 3.39 (2.25-4.53) |
| Mauritania | 147 (49-282) | 15.39 (5.24-29.03) | 272 (98-503) | 13.16 (4.67-24.65) | -0.76 (-1.83-0.32) |
| Mauritius | 120 (47-220) | 16.62 (6.58-29.8) | 661 (271-1129) | 35.74 (14.9-60.93) | 4.35 (3.85-4.86) |
| Mexico | 3227 (1251-5602) | 8.59 (3.31-14.71) | 16803 (6794-29639) | 13.29 (5.42-23) | 3.4 (2.58-4.22) |
| Micronesia (Federated States of) | 21 (8-39) | 44.11 (16.62-80.4) | 44 (16-81) | 61.12 (21.92-107.37) | 2 (1.09-2.91) |
| Monaco | 2 (1-4) | 2.39 (0.96-4.52) | 3 (1-6) | 2.71 (0.99-4.95) | 0.84 (-0.66-2.35) |
| Mongolia | 31 (10-63) | 3.03 (1.01-6.15) | 50 (18-97) | 2.28 (0.82-4.28) | -0.78 (-1.69-0.13) |
| Montenegro | 12 (5-21) | 1.91 (0.75-3.33) | 16 (6-29) | 1.65 (0.6-3.02) | 0.49 (0.08-0.91) |
| Morocco | 1107 (407-2251) | 7.87 (2.81-15.8) | 2675 (1070-4757) | 7.69 (3.09-13.82) | 1.14 (0.57-1.71) |
| Mozambique | 224 (77-494) | 4.59 (1.58-10.03) | 559 (186-1164) | 6.07 (2.07-12.63) | 0.96 (-0.55-2.5) |
| Myanmar | 1154 (386-2344) | 5.06 (1.78-10.08) | 1832 (654-3441) | 3.84 (1.34-7.24) | -0.38 (-0.96-0.21) |
| Namibia | 38 (15-73) | 6.01 (2.31-11.22) | 76 (29-145) | 5.58 (2.12-10.52) | -0.2 (-1.23-0.84) |
| Nauru | 1 (0-2) | 26.2 (9.12-51.79) | 3 (1-5) | 44.43 (16.17-86.44) | 1.8 (0.83-2.79) |
| Nepal | 452 (157-870) | 5.2 (1.84-10.04) | 1256 (452-2600) | 5.46 (1.96-11.02) | 1.25 (0.39-2.13) |
| Netherlands | 277 (101-504) | 1.35 (0.49-2.47) | 683 (271-1195) | 1.7 (0.69-3) | 1.61 (0.75-2.47) |
| New Zealand | 75 (30-135) | 1.96 (0.76-3.49) | 204 (80-364) | 2.43 (0.96-4.32) | 1.67 (0.99-2.36) |
| Nicaragua | 99 (36-184) | 6.66 (2.45-12.2) | 520 (200-943) | 10.55 (4.16-19.11) | 3.17 (2.21-4.13) |
| Niger | 140 (50-271) | 5.45 (1.96-10.34) | 265 (91-545) | 3.36 (1.14-6.7) | -1.62 (-3.16--0.06) |
| Nigeria | 2738 (1042-5096) | 6.74 (2.59-12.24) | 4053 (1585-7555) | 4.72 (1.86-8.71) | -2.04 (-3.29--0.77) |
| Niue | 1 (0-1) | 23.46 (8.73-43.69) | 1 (0-2) | 45.44 (14.37-89.54) | 2.8 (2.51-3.08) |
| North Macedonia | 24 (9-44) | 1.3 (0.51-2.42) | 34 (13-63) | 1.1 (0.41-2.04) | 0.41 (0.06-0.77) |
| Northern Mariana Islands | 6 (2-11) | 39.13 (15.81-67.65) | 21 (8-35) | 42.26 (16.77-70.33) | 2.45 (1.42-3.49) |
| Norway | 64 (24-113) | 0.83 (0.31-1.48) | 149 (55-266) | 1.25 (0.47-2.2) | 1.76 (0.76-2.76) |
| Oman | 69 (25-130) | 10.47 (3.9-19.65) | 245 (94-469) | 12.11 (4.86-21.88) | 1.47 (0.18-2.78) |
| Pakistan | 5050 (1769-9037) | 9.28 (3.38-16.27) | 11829 (4185-22696) | 9.62 (3.41-18.26) | -0.18 (-1.16-0.82) |
| Palau | 2 (1-4) | 24.06 (9.24-42.89) | 8 (3-14) | 38.34 (13.76-68.77) | 2.77 (2.33-3.2) |
| Palestine | 111 (43-197) | 13.36 (5.17-23.08) | 226 (91-425) | 9.03 (3.6-16.25) | -1.06 (-2.29-0.18) |
| Panama | 66 (24-118) | 4.58 (1.65-8.24) | 302 (112-544) | 6.76 (2.52-12.22) | 2.32 (1.8-2.85) |
| Papua New Guinea | 152 (51-297) | 7.9 (2.76-14.94) | 370 (124-693) | 6.66 (2.34-11.85) | -0.44 (-1.45-0.57) |
| Paraguay | 222 (81-402) | 10.54 (3.85-19.03) | 525 (202-975) | 9.19 (3.53-16.96) | 0.44 (-0.23-1.1) |
| Peru | 1808 (674-3292) | 16.16 (6.07-28.87) | 4977 (1772-9188) | 14.92 (5.31-27.65) | 0.46 (-0.1-1.03) |
| Philippines | 1642 (597-2961) | 6.44 (2.41-11.59) | 7607 (2604-14499) | 9.46 (3.31-17.5) | 2.67 (1.75-3.59) |
| Poland | 1167 (467-2129) | 2.69 (1.05-4.87) | 788 (300-1400) | 1.05 (0.4-1.87) | -2.68 (-3.4--1.95) |
| Portugal | 583 (234-1054) | 4.36 (1.71-7.84) | 880 (324-1571) | 2.86 (1.06-5.04) | -0.34 (-1.3-0.63) |
| Puerto Rico | 708 (304-1214) | 20.21 (8.55-34.8) | 1392 (588-2326) | 18.72 (7.6-31.15) | 2.06 (1.34-2.78) |
| Qatar | 11 (3-22) | 9.66 (3.2-17.29) | 96 (39-180) | 13.57 (5.33-24.56) | 0.5 (-1.38-2.42) |
| Republic of Korea | 1909 (752-3382) | 8.07 (3.09-14.49) | 4006 (1463-7556) | 4.35 (1.59-8.24) | -0.03 (-0.54-0.47) |
| Republic of Moldova | 10 (4-19) | 0.26 (0.1-0.48) | 27 (10-51) | 0.43 (0.16-0.83) | 2.17 (1.6-2.73) |
| Romania | 210 (82-392) | 0.79 (0.3-1.49) | 417 (156-729) | 1.08 (0.4-1.89) | 3.09 (2.3-3.89) |
| Russian Federation | 1589 (603-2937) | 0.89 (0.33-1.59) | 3051 (1107-5235) | 1.23 (0.44-2.1) | 1.35 (0.76-1.93) |
| Rwanda | 159 (50-329) | 6.62 (2.24-12.91) | 262 (76-514) | 5.09 (1.51-9.76) | -1.5 (-3.03-0.06) |
| Saint Kitts and Nevis | 8 (3-14) | 22.53 (8.75-39.32) | 16 (6-30) | 24.49 (9.69-42.48) | 1.72 (1.35-2.09) |
| Saint Lucia | 12 (5-22) | 14.95 (5.72-25.94) | 37 (15-65) | 15.63 (6.07-27.18) | 1.91 (1.43-2.39) |
| Saint Vincent and the Grenadines | 8 (3-13) | 11.39 (4.47-19.66) | 22 (9-40) | 16.16 (6.57-28.65) | 2.96 (2.51-3.4) |
| Samoa | 21 (8-38) | 24.68 (9.36-44.36) | 56 (19-99) | 39.07 (13.43-68.89) | 2.14 (1.4-2.88) |
| San Marino | 1 (0-1) | 1.9 (0.74-3.37) | 1 (0-2) | 0.96 (0.35-1.84) | 0.12 (-1.08-1.34) |
| Sao Tome and Principe | 5 (2-10) | 8.82 (3.27-17.33) | 9 (3-18) | 8.61 (3.02-16.8) | -0.34 (-1.4-0.74) |
| Saudi Arabia | 1165 (449-2068) | 19.51 (7.86-34.14) | 6414 (2351-12022) | 27.94 (10.65-50.86) | 1.53 (0.43-2.64) |
| Senegal | 349 (129-662) | 11.16 (4.09-20.81) | 607 (241-1216) | 7.88 (3.1-15.66) | -1.2 (-2.29--0.09) |
| Serbia | 260 (98-464) | 2.62 (0.98-4.67) | 288 (114-512) | 1.69 (0.66-3.06) | -0.22 (-0.82-0.37) |
| Seychelles | 8 (3-15) | 14.56 (5.55-25.9) | 20 (7-35) | 17.28 (6.21-30.62) | 1.26 (1-1.52) |
| Sierra Leone | 127 (44-231) | 6.62 (2.29-11.88) | 166 (60-321) | 4.54 (1.66-8.47) | -1.62 (-2.75--0.48) |
| Singapore | 161 (58-276) | 8.01 (2.93-13.93) | 449 (178-785) | 5.36 (2.13-9.4) | 1.83 (1.2-2.47) |
| Slovakia | 136 (56-251) | 2.26 (0.91-4.19) | 135 (55-252) | 1.4 (0.58-2.6) | -0.64 (-1.12--0.16) |
| Slovenia | 23 (9-41) | 0.95 (0.39-1.67) | 37 (14-68) | 0.72 (0.27-1.34) | 0.89 (0.1-1.69) |
| Solomon Islands | 36 (11-71) | 26.39 (8.39-50.75) | 68 (24-121) | 18.07 (6.69-31.66) | -0.96 (-2.02-0.1) |
| Somalia | 190 (66-386) | 9.93 (3.66-19.97) | 503 (153-1057) | 9.88 (3.27-20.11) | -0.03 (-1.89-1.87) |
| South Africa | 1161 (414-2088) | 5.63 (2.02-9.98) | 2607 (1062-4719) | 5.63 (2.27-10.16) | 0.99 (0.37-1.6) |
| South Sudan | 206 (68-419) | 8.86 (3.01-17.77) | 400 (132-750) | 12.06 (3.9-23.26) | 0.54 (-1.02-2.12) |
| Spain | 1580 (599-2835) | 2.92 (1.1-5.24) | 2008 (748-3828) | 1.55 (0.57-2.82) | -0.88 (-1.83-0.07) |
| Sri Lanka | 449 (160-850) | 4.44 (1.61-8.22) | 1008 (350-2059) | 3.8 (1.35-7.69) | 0.51 (0.11-0.91) |
| Sudan | 772 (295-1525) | 7.98 (2.98-16.29) | 1540 (594-2960) | 7.08 (2.83-13.74) | -0.55 (-1.58-0.48) |
| Suriname | 53 (21-94) | 20.04 (8.16-35.3) | 142 (54-263) | 21.98 (8.26-40) | 1.36 (1-1.73) |
| Sweden | 176 (65-312) | 1.02 (0.38-1.81) | 470 (178-865) | 1.71 (0.65-3.08) | 2.66 (1.49-3.84) |
| Switzerland | 162 (65-286) | 1.45 (0.59-2.54) | 275 (106-510) | 1.18 (0.46-2.1) | 1 (-0.01-2.01) |
| Syrian Arab Republic | 797 (309-1425) | 15.3 (5.88-27.59) | 1529 (603-2916) | 11.33 (4.57-21.29) | -0.11 (-1.2-1) |
| Taiwan (Province of China) | 2957 (1185-5115) | 22.15 (8.84-37.83) | 5701 (2161-9833) | 12.98 (4.91-22.43) | 0.32 (-0.19-0.83) |
| Tajikistan | 8 (3-16) | 0.3 (0.11-0.6) | 18 (6-36) | 0.3 (0.11-0.59) | -0.45 (-1.45-0.56) |
| Thailand | 2837 (1067-5290) | 8.95 (3.46-16.31) | 9593 (3552-17761) | 8.71 (3.27-16.19) | 1.45 (0.98-1.92) |
| Timor-Leste | 13 (5-26) | 5.75 (2.06-10.84) | 37 (12-70) | 4.58 (1.46-8.57) | 0.55 (-0.58-1.7) |
| Togo | 63 (23-129) | 6 (2.18-11.51) | 185 (70-353) | 5.73 (2.16-10.64) | 0.14 (-1.4-1.71) |
| Tokelau | 0 (0-0) | 16.5 (5.82-31.44) | 0 (0-1) | 24.86 (9.05-44.23) | 2.15 (1.93-2.37) |
| Tonga | 7 (2-13) | 12.43 (4.27-22.29) | 14 (5-24) | 17.49 (6.41-29.81) | 1.15 (0.56-1.74) |
| Trinidad and Tobago | 99 (40-168) | 12.17 (4.96-20.66) | 445 (179-774) | 22.66 (9.07-39.67) | 3.96 (3.54-4.38) |
| Tunisia | 249 (94-460) | 5.33 (2.04-9.72) | 712 (253-1345) | 5.59 (2.06-10.49) | 1.48 (1.01-1.96) |
| Turkey | 4485 (1742-8205) | 13.85 (5.44-25.23) | 7994 (2909-14309) | 8.7 (3.16-15.45) | -0.17 (-0.64-0.29) |
| Turkmenistan | 38 (13-74) | 1.96 (0.68-3.76) | 129 (47-269) | 3.18 (1.17-6.52) | 2.07 (1.25-2.91) |
| Tuvalu | 2 (1-3) | 23.65 (8.96-44.41) | 3 (1-6) | 30.57 (10.85-57.22) | 1.17 (0.83-1.52) |
| Uganda | 339 (111-656) | 5.98 (2.02-11.38) | 891 (280-1763) | 7.01 (2.21-13.34) | -0.03 (-1.71-1.68) |
| Ukraine | 12 (5-23) | 0.02 (0.01-0.03) | 208 (71-414) | 0.26 (0.09-0.52) | 13.72 (12.16-15.3) |
| United Arab Emirates | 43 (16-77) | 8.46 (3.02-14.78) | 379 (134-712) | 13.1 (4.24-24.48) | 2.69 (0.58-4.85) |
| United Kingdom | 1280 (470-2306) | 1.33 (0.48-2.37) | 1567 (607-2732) | 1.04 (0.41-1.81) | 0.5 (-0.41-1.42) |
| United Republic of Tanzania | 333 (116-688) | 3.35 (1.16-6.64) | 687 (242-1453) | 3.02 (1.05-6.32) | -0.7 (-2-0.63) |
| United States of America | 13585 (5436-23318) | 4.18 (1.68-7.1) | 56243 (22916-91193) | 9.57 (3.9-15.58) | 3.43 (2.85-4) |
| United States Virgin Islands | 7 (3-12) | 8.22 (3.2-14.87) | 14 (5-25) | 7.78 (3.1-14.09) | 1.9 (1.38-2.41) |
| Uruguay | 173 (66-309) | 4.39 (1.66-7.83) | 211 (79-375) | 3.59 (1.35-6.38) | 0.44 (-0.12-1) |
| Uzbekistan | 103 (32-232) | 0.96 (0.29-2.18) | 352 (134-653) | 1.33 (0.49-2.4) | 0.22 (-0.67-1.12) |
| Vanuatu | 7 (2-13) | 12.17 (4.42-23.91) | 18 (6-34) | 11.28 (3.81-21.19) | 0.1 (-1.07-1.3) |
| Venezuela (Bolivarian Republic of) | 500 (182-915) | 5.44 (2-9.93) | 2751 (1072-5311) | 9.06 (3.55-17.31) | 2.64 (1.94-3.34) |
| Viet Nam | 2334 (796-4489) | 6.08 (2.1-11.52) | 5601 (1890-10630) | 5.89 (1.98-11.2) | 1 (0.43-1.58) |
| Yemen | 479 (176-950) | 10.09 (3.62-19.71) | 904 (351-1771) | 6.27 (2.45-12.01) | -1.47 (-2.78--0.14) |
| Zambia | 335 (121-651) | 12.85 (4.57-24.44) | 813 (279-1609) | 12.58 (4.54-23.87) | -0.61 (-2.15-0.95) |
| Zimbabwe | 182 (66-356) | 4.76 (1.77-9.12) | 341 (128-710) | 5.02 (1.87-10.08) | 0.42 (-0.88-1.73) |
